# Chiral Bifacial Indacenodithiophene‐Based Hole‐Transport Materials With Chirality‐Induced Spin Selectivity: Chirality‐Spin Polarity Correspondence and Perovskite Passivation

**DOI:** 10.1002/smll.75074

**Published:** 2026-08-08

**Authors:** Shuang Li, Fumitaka Ishiwari, Ryosuke Nishikubo, Akinori Saeki

**Affiliations:** ^1^ Department of Applied Chemistry Graduate School of Engineering The University of Osaka Suita Osaka Japan; ^2^ Innovative Catalysis Science Division Institute for Open and Transdisciplinary Research Initiatives (ICS‐OTRI) The University of Osaka Suita Osaka Japan; ^3^ Graduate School of Urban Environmental Sciences Tokyo Metropolitan University Hachioji Tokyo Japan; ^4^ PRESTO Japan Science and Technology Agency (JST) Kawaguchi Saitama Japan

**Keywords:** chiral bifacial molecules, chirality‐induced spin selectivity, hole transport materials, indacenodithiophene, perovskite solar cells

## Abstract

Chirality‐induced spin selectivity (CISS) is emerging as a key element for spin‐dependent functions in organic electronics. We previously developed a chiral bifacial indacenodithiophene (IDT) backbone that shows strong CISS in both *π*‐conjugated polymers and non‐fullerene acceptors (NFAs). Building on this platform, here we report triarylamine‐functionalized, chiral bifacial IDT‐based hole‐transport materials (HTMs). Spin‐coated films of (*R*,*R*)‐**1** and (*S*,*S*)‐**1** exhibit pronounced CISS with spin polarization (SP) ≈ 60%. Across our chiral‐bifacial IDT series, *π*‐conjugated polymers, NFAs, and these HTMs, we observe a consistent chirality‐spin polarity correspondence: (*S*,*S*) chirality yields negative SP, whereas (*R*,*R*) yields positive SP, representing a platform‐level demonstration across three material classes. In perovskite solar cells, these HTMs also act as effective surface passivators; devices passivated with homochiral (*R*,*R*)‐**1** gave higher efficiencies than *meso*‐ or racemic counterparts. Space–charge‐limited current measurements likewise indicate approximately threefold higher hole mobility for the homochiral film relative to the racemate. While a direct causal connection to the CISS effect remains to be demonstrated, these observations are consistent with a CISS‐assisted, efficient spin‐selective transport mechanism. Taken together, this work establishes a chirality‐spin polarity correspondence and demonstrates effective perovskite passivation, with higher hole mobility correlating with homochirality, highlighting new opportunities for homochiral materials in device science.

## Graphical abstract

Homochiral bifacial indacenodithiophene (IDT)‐based hole‐transport materials form amorphous spin‐coated films showing pronounced chirality‐induced spin selectivity (CISS) with spin polarization ≈ 60%, and reveal a robust chirality–spin‐polarity correspondence. Applied as a passivation layer in perovskite solar cells, they improve device performance over *meso* and racemic analogues, highlighting homochirality for interfacial engineering.

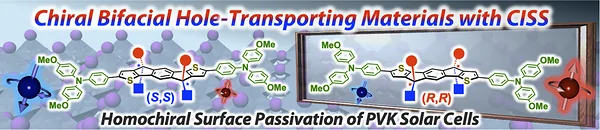

## 1 Introduction

Recently, stimulated by the emergence of the chirality‐induced spin selectivity (CISS) effect, chiral organic electronic materials have attracted increasing attention, opening new possibilities for exploring spin‐related phenomena in molecular systems [1, 2, 3, 4, 5]. The CISS effect is a phenomenon in which spin‐polarized currents exhibit different conductivities when transmitted through chiral materials [6, 7]. Although electron spin itself is not chiral, a flow of spin‐polarized electrons can be regarded as a chiral physical quantity, analogous to circularly polarized light showing different responses in homochiral media. Owing to the CISS effect, chiral molecular films can function as spin filters, enabling the generation of spin‐polarized currents even without ferromagnetic electrodes. Such spin‐polarized currents have been demonstrated to be effective in diverse applications, including circularly polarized organic light‐emitting diodes (CP‐OLEDs) [8], oxygen evolution [9], and oxygen reduction reactions [10], thereby opening new avenues for chiral materials in electronic and energy devices. For example, homochiral non‐fullerene acceptors (NFAs) exhibiting the CISS effect have been reported, and their incorporation has been shown to enhance the efficiency of photocatalytic water splitting [11].

Our group has also contributed to this field by developing a series of chiral bifacial π‐conjugated materials based on the indacenodithiophene (IDT) backbone [12, 13]. As illustrated in Figure 1a, conventional IDT derivatives are achiral when the two substituents on sp^3^‐bridged carbons are identical, whereas introducing different substituents on sp^3^‐bridged carbons generates a chiral IDT framework. This structural design endows the IDT core with chirality, polarity, a permanent dipole moment, and the ability to exhibit circular dichroism (CD) and CISS responses. For the first time, we demonstrated that bifacial IDT‐based conjugated polymers (Figure 1b) [12] exhibit pronounced CISS responses, with spin polarization (SP = (*I*_up_ − *I*_down_) / (*I*_up_ + *I*_down_) × 100%) values of nearly 70% in amorphous spin‐coated thin films. More recently, we extended this molecular design strategy NFAs (Figure 1c) [13] and similarly observed strong CISS effects with SP values of ∼60%–70%. Importantly, bulk heterojunction solar cells employing these homochiral bifacial NFAs exhibited higher power conversion efficiencies (PCEs) than those using their optically inactive counterparts. In this work, we have further extended this molecular design by developing chiral bifacial hole transport materials (HTMs) based on the IDT backbone (Figure 1d), in which electron‐rich triphenylamine (TPA) [14] units were introduced to enable efficient hole transport. These chiral bifacial hole transport materials, (*R*,*R*)‐**1** and (*S*,*S*)‐**1**, were found to form amorphous thin films by spin coating, exhibiting SP values of around 60%. In our previous studies, we had discovered that bifacial hole‐transporting indenofluorene derivatives [15, 16] could act as effective passivation molecules [17, 18, 19, 20, 21] for perovskite (PVK) layers; however, optical resolution was not achieved at that time, and thus the passivation properties of homochiral species remained unexplored. Recently, chiral molecules have attracted increasing attention as interfacial modifiers in perovskite solar cells [22, 23, 24, 25]. Notably, Ran and Chen et al. reported that perovskite solar cells (PSCs) modified with homochiral 2D perovskite interlayers, derived from (*R*)‐ or (*S*)‐*α*‐methylbenzylamine and exhibiting strong CISS, showed higher open‐circuit voltage (*V*_OC_) and fill factor (FF), along with reduced hysteresis [23]. Spin‐selective transport, which is likely attributable to CISS, facilitated hole extraction and contributed to these performance gains [23]. In addition, CISS may impede carrier backscattering through spin‐velocity locking, which can enhance effective charge transport in chiral films [5]. However, the integration of CISS‐active materials into photovoltaic devices remains underexplored [26], and examples of chiral charge‐transport materials that combine spin‐selective transport with interfacial passivation in photovoltaic devices are still limited. Motivated by these backgrounds, the present homochiral bifacial IDT‐based HTMs were applied to the top surfaces of PVK layers, where they functioned as passivation molecules and led to improved performance in PSCs.

**FIGURE 1:**
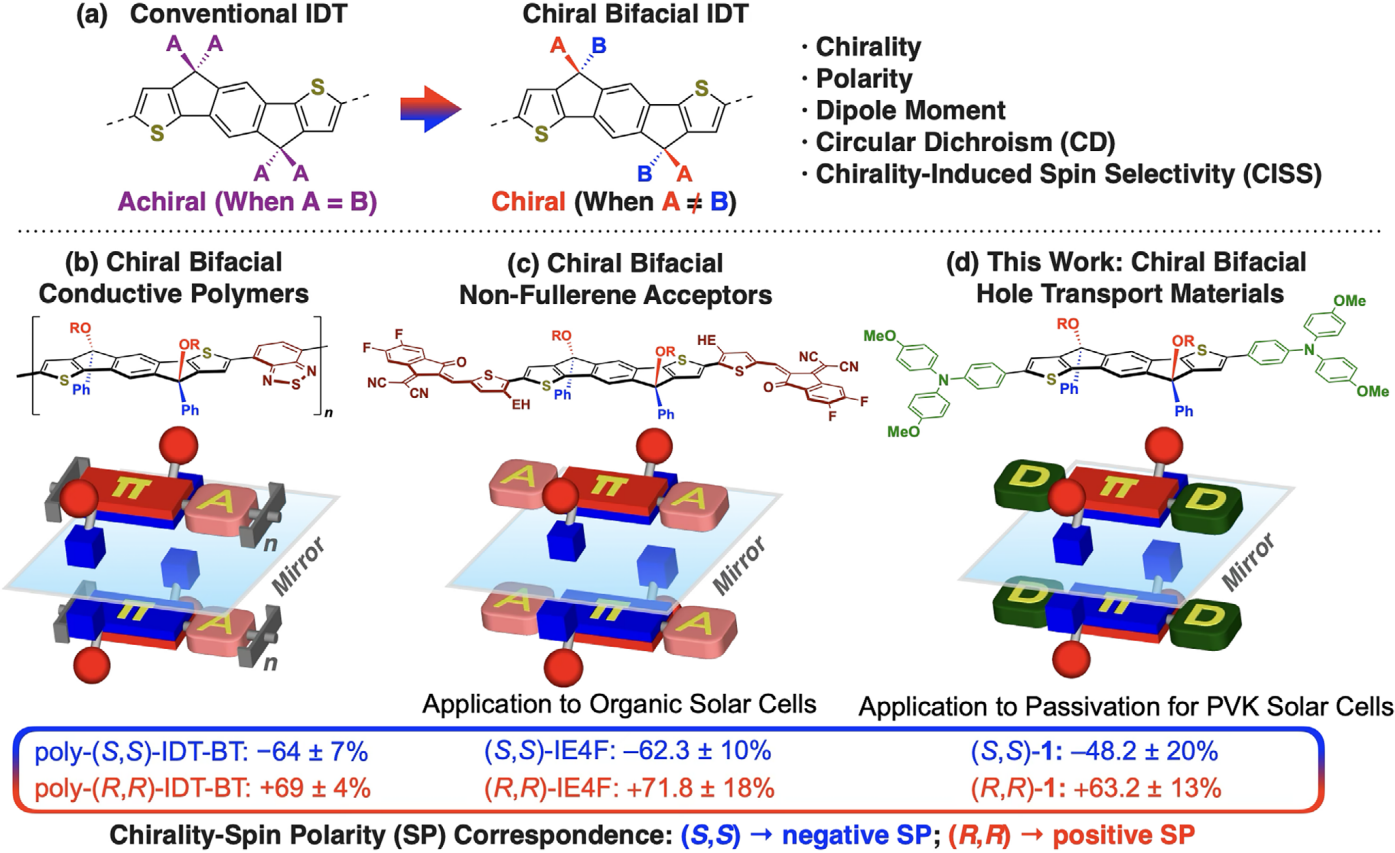
(a) Chemical structures of achiral conventional indacenodithiophene (IDT) and chiral bifacial IDT. Chemical and schematic structures of (b) chiral bifacial conductive polymers (previous work), (c) non‐fullerene acceptors (previous work), and (d) hole transport materials (this work) derived from chiral bifacial IDT skeleton.

## 2 Results and Discussion

### 2.1 Synthesis and Theoretical Calculations

As shown in Figure 1d, we designed the molecular structure based on a previously reported IDT‐cored HTM bearing methoxy‐substituted TPA units [14]. The *meso*‐**2**, *rac*‐**2**, (*S*,*S*)‐**2**, and (*R*,*R*)‐**2** molecules were prepared as reported in our previous work [13], in which *meso*‐**2** and *rac*‐**2** were separated by SiO_2_ column chromatography, followed by chiral HPLC resolution of *rac*‐**2** using a chiral stationary phase to afford (*S*,*S*)‐**2** and (*R*,*R*)‐**2**. As shown in Figure 2a, each IDT‐monomer was applied for Pd‐catalyzed Suzuki‐Miyaura cross coupling with the boronated TPA counterpart to give the HTMs, namely, *meso*‐**1**, *rac*‐**1**, (*S*,*S*)‐**1** and (*R*,*R*)‐**1** in ∼80% yield. The chemical structures of the newly obtained compounds were sufficiently characterized by NMR spectroscopy, fast Fourier transform infrared (FT‐IR) spectroscopy, as well as high‐resolution matrix‐assisted laser desorption/ionization time‐of‐flight (MALDI‐TOF) mass spectrometry (see Supporting Information for details). Thermogravimetric analysis (TGA, Figure S1) of *meso*‐**1**, r*ac*‐**1**, (*S*,*S*)‐**1** and (*R*,*R*)‐**1** indicates their enough thermal stability for PSCs application. Density functional theory (DFT) calculations of *meso*‐**1**, (*S*,*S*)‐**1,** and (*R*,*R*)‐**1** were performed to compute the molecular orbitals and electrostatic potential (ESP). The calculated derivatives, *meso*‐**1**, (*S*,*S*)‐**1**, and (*R*,*R*)‐**1**, were confirmed to be D–π–D‐type molecules based on their molecular orbital distributions (Figure S2). The highest occupied molecular orbital (HOMO) is spread over TPAs through the IDT π‐core, and the lowest unoccupied molecular orbital (LUMO) is partly extended to the flanked phenyl groups of TPAs. As shown in Figure S3, The hydrophilic oligo(ethylene glycol) side exhibits more negative electronic potential over hydrophobic phenyl group sides. Considering that oligo(ethylene glycol) side chains are soft, flexible, and impose minimal steric hindrance, they are expected to more readily establish contact with the underlying PVK films. This viewpoint is further supported by water contact angle (CA) measurements (Figure S4). In contrast to the chlorobenzene (CB)‐treated PVK film (CA ∼67°), the PVK films treated with *meso*‐**1**, *rac*‐**1**, and (*R*,*R*)‐**1** exhibit much larger CAs (∼80°), indicative of enhanced surface hydrophobicity. This can be attributed to the preferential exposure of phenyl groups on the film surface. Efficient surface passivation is expected due to the close contact between the electron‐rich oligo(ethylene glycol) side and the PVK film, complemented by the lone pairs on the nitrogen atoms of the TPA groups. Reflecting the difference in molecular symmetry, *rac*‐**1**, (*S*,*S*)‐**1**, and (*R*,*R*)‐**1** have higher polarity than *meso*‐**1**, resulting in lower retention factor (R_f_) values (0.53) in SiO_2_‐based thin‐layer chromatography (TLC) compared with the R_f_ value of *meso*‐**1** (0.68) (*n*‐hexane:EtOAc = 1:4, Table 1). This result suggests that the bifacial isomers show higher surface adhesion properties on solid surfaces than *meso*‐isomer [15].

**FIGURE 2:**
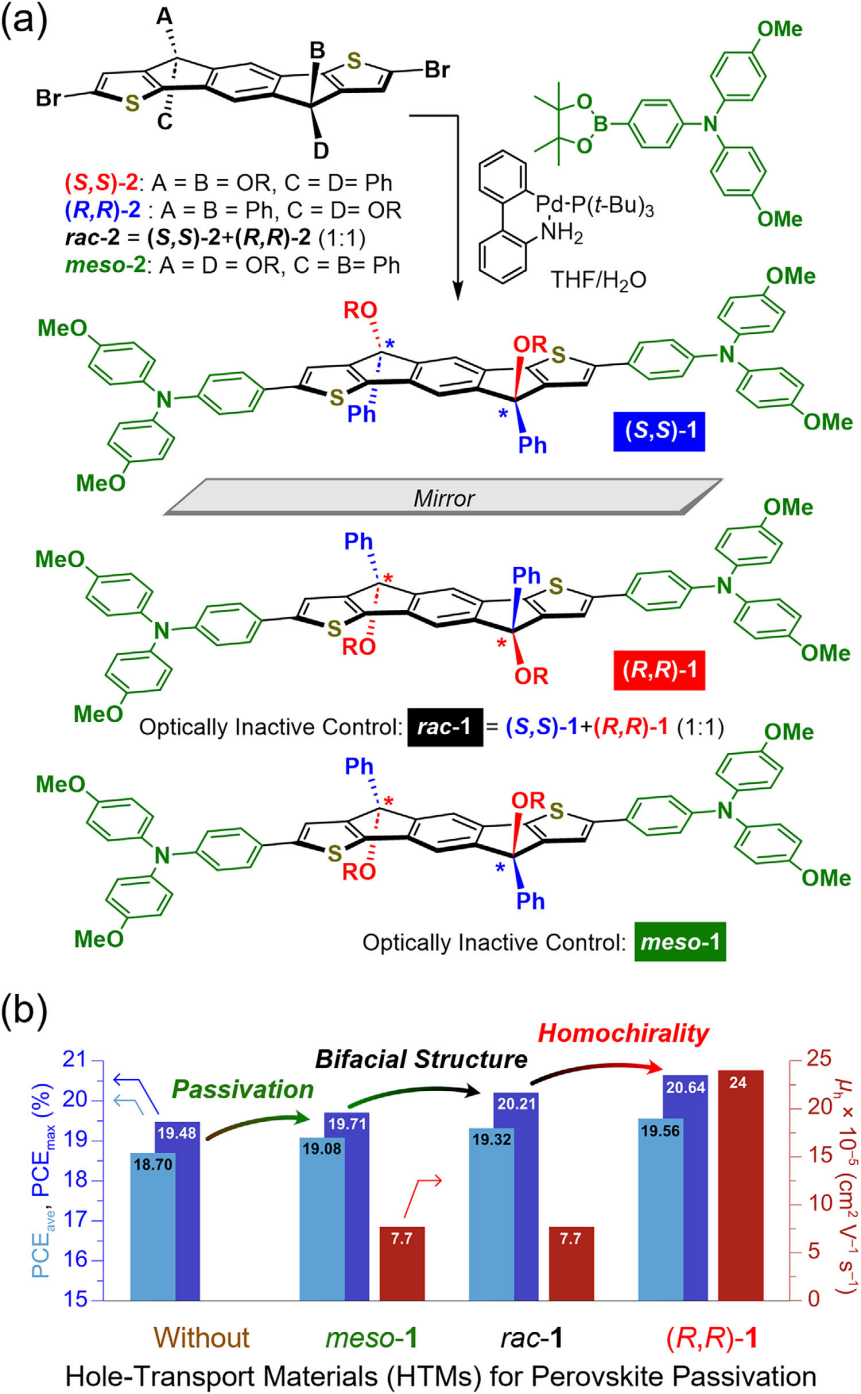
(a) Synthesis of (*S*,*S*)‐**1**, (*R*,*R*)‐**1**, *rac*‐**1** and *meso*‐**1**. (b) Summary of PCE values for PSCs with and without **1**s.

**TABLE 1:** Summary of the optical and electronic properties of *meso*‐**1**, *rac*‐**1**, and (*R*,*R*)‐**1** molecules.

	HOMO [eV]	*E*_g_ [eV]	LUMO [eV]	λ_max_ [nm]^a^	λ_max_ [nm]^b^ ^]^	R_f_ value^c^
*meso*‐**1**	−5.33	2.42	−2.91	449	461	0.68
*rac*‐**1**	−5.34	2.42	−2.92	449	459	0.53
(*R*,*R*)‐**1**	−5.31	2.42	−2.89	449	461	0.53

^a^ Photoabsorption maximum in THF

^b^ Photoabsorption maximum in the thin film states

^c^ SiO_2_‐based TLC, *n*‐hexane:EtOAc = 1:4.

### 2.2 Photophysical and Chiroptical Properties

We measured CD and UV‐Vis absorption for **1**s in both of solution and thin film states. (*S*,*S*)‐**1** and (*R*,*R*)‐**1** showed mirror symmetric CD spectra in both solution and thin film states (Figure 3a), demonstrating enantiomerically pure natures of (*S*,*S*)‐**1** and (*R*,*R*)‐**1**. No angle‐ and side‐dependence CD spectra (Figure S5) and the absence of XRD diffraction peaks (Figure S6) of the neat thin films support that the CD originates from the molecule rather than the high‐order structures. These HTMs exhibit identical UV‐Vis absorption spectra with the maximum absorption at 451 nm in 10 µm tetrahydrofuran (THF) solution, and the maximum absorption peaks are slightly red‐shifted to 459 nm in thin film states (Figure 3b). The HOMO (or valence band maximum) energy levels of PVK, *meso*‐**1**, *rac*‐**1** and (*R*,*R*)‐**1** were determined to be −5.83, −5.33, −5.34 and −5.31 eV, respectively, using photoelectron yield spectroscopy (PYS) (Figure S7). The bandgap energies (*E*_g_) of PVK (FA_0.85_MA_0.1_Cs_0.05_PbI_3_, FA: formamidinium, MA: methylammonium), *meso*‐**1**, *rac*‐**1** and (*R*,*R*)‐**1** were determined to be 1.57, 2.42, 2.42 and 2.42 eV, respectively from the onset of UV‐Vis absorption spectra in the film states (Figure S8), and the LUMO (or conduction band minimum) energy levels were calculated by HOMO + *E*_g_. The obtained energy diagram is presented in Figure 3c, and the specific values are summarized in Table 1. These HTMs possess well‐matched HOMO level alignments relative to the PVK and the hole transport layer (HTL: spiro‐OMeTAD), thereby they are expected to facilitate efficient hole extraction and transport.

**FIGURE 3:**
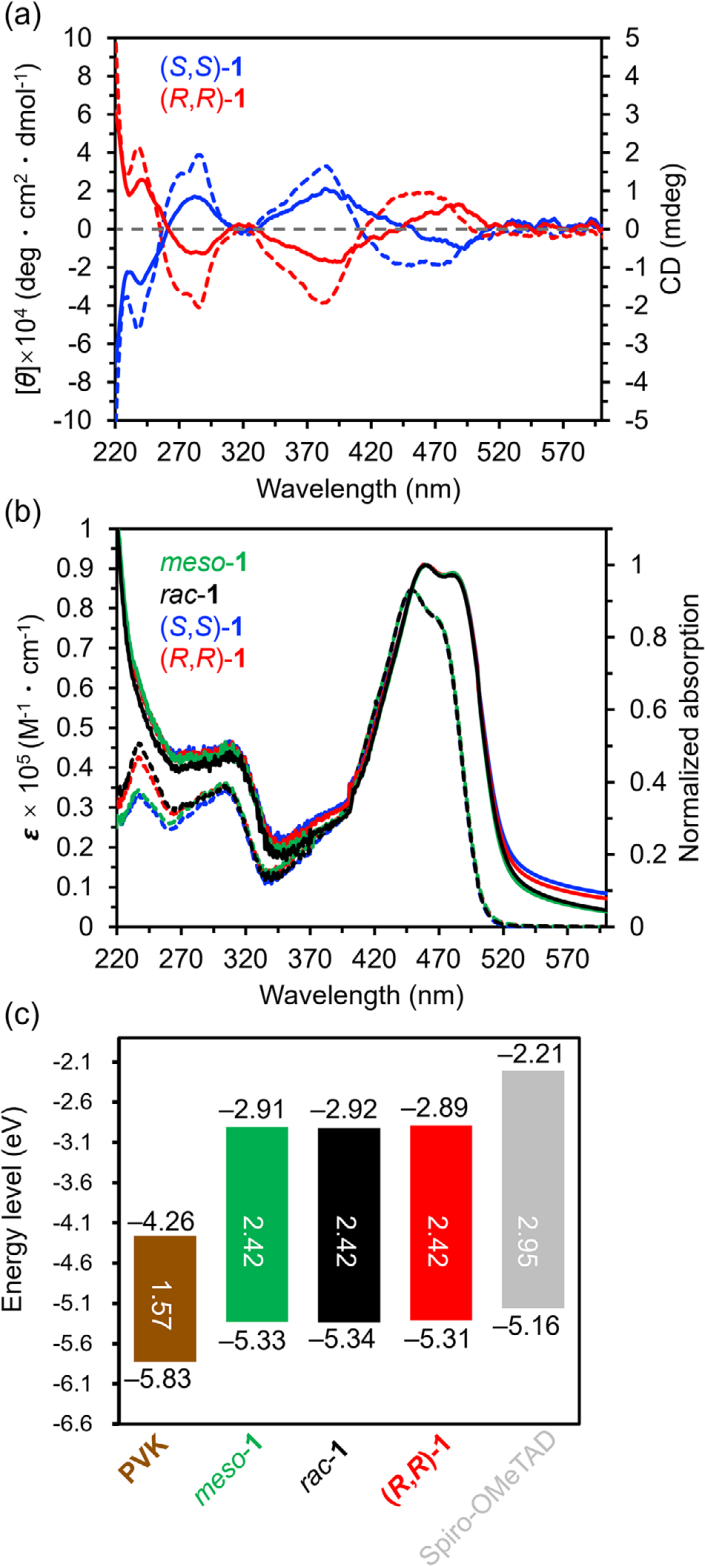
(a) CD spectra of (*S*,*S*)‐**1** and (*R*,*R*)‐**1** in THF (10 µm) (dotted lines) and in thin film states (solid lines). (b) UV–vis absorption of *meso*‐**1**, *rac*‐**1**, (*S*,*S*)‐**1**, and (*R*,*R*)‐**1** in THF (10 µM) (dotted lines) and in thin film states (solid lines). (c) Energy diagram of PVK (FA_0.85_MA_0.1_Cs_0.05_PbI_3_), *meso*‐**1**, *rac*‐**1**, (*R*,*R*)‐**1** and spiro‐OMeTAD.

### 2.3 CISS Properties and Chirality‐Spin Polarity Correspondence

CISS is a phenomenon in which up or down‐spin‐polarized electrons preferentially pass through a chiral substance to generate spin‐polarized current. The CISS effect has attracted significant attention as a novel approach for spin manipulation. In our previous research [12], chiral bifacial IDT‐benzothiadiazole (BT) polymers achieved the SP value of nearly 70% in simply prepared spin‐coat thin films, possibly owing to the proximity of the chiral centers to the π‐conjugated backbone. Inspired by this, CISS characterization of optically active (*S*,*S*)‐**1** and (*R*,*R*)‐**1** was investigated using spin polarized conductive atomic force microscopy (AFM). (*S*,*S*)‐**1** and (*R*,*R*)‐**1** thin films were fabricated by spin coating their 5 mg mL^−1^ solution in CB at 1500 rpm onto highly oriented pyrolytic graphite (HOPG). Tapping mode AFM was applied to observe the film morphology of (*S*,*S*)‐**1** and (*R*,*R*)‐**1** thin films on HOPG (Figure S9a,b). We confirmed the film thickness (*L*) is 24.0 ± 6.0 nm with root‐mean‐square surface roughness (*R*_a_) of 0.42 nm for (*S*,*S*)‐**1** neat film, and *L* is 20.6 ± 5.1 nm with *R*_a_ of 0.33 nm for (*R*,*R*)‐**1** neat film. Nd magnet was set on the top of the paramagnetic Co‐Cr cantilever during the measurement with “S” or “N” pole toward to the cantilever to maintain “down” or “up” spin polarized electrons, respectively. More than 100 sets of current‐voltage (*I*–*V*) curves under either up or down polarized condition at different locations on samples were collected to obtain the averaged up (*I*_up_) or down (*I*_down_)‐spin polarized current. Consequently, down‐spin polarized electrons preferentially pass through the (*S*,*S*)‐**1** thin film, resulting in *I*_down_ exceeding *I*_up_, whereas (*R*,*R*)‐**1** thin films selectively transport the up‐spin polarized current, yielding a larger *I*_up_ than *I*_down_ (Figure 4a,b and Figure S9c,d). The results indicate that the CISS effect has been observed in chiral bifacial HTMs thin films. According to the averaged spin‐polarized currents, the SP values were calculated to be −48.2 ± 19.6 and + 63.2 ± 12.5% (Figure 4c) for (*S*,*S*)‐**1** and (*R*,*R*)‐**1** thin films, respectively.

**FIGURE 4:**
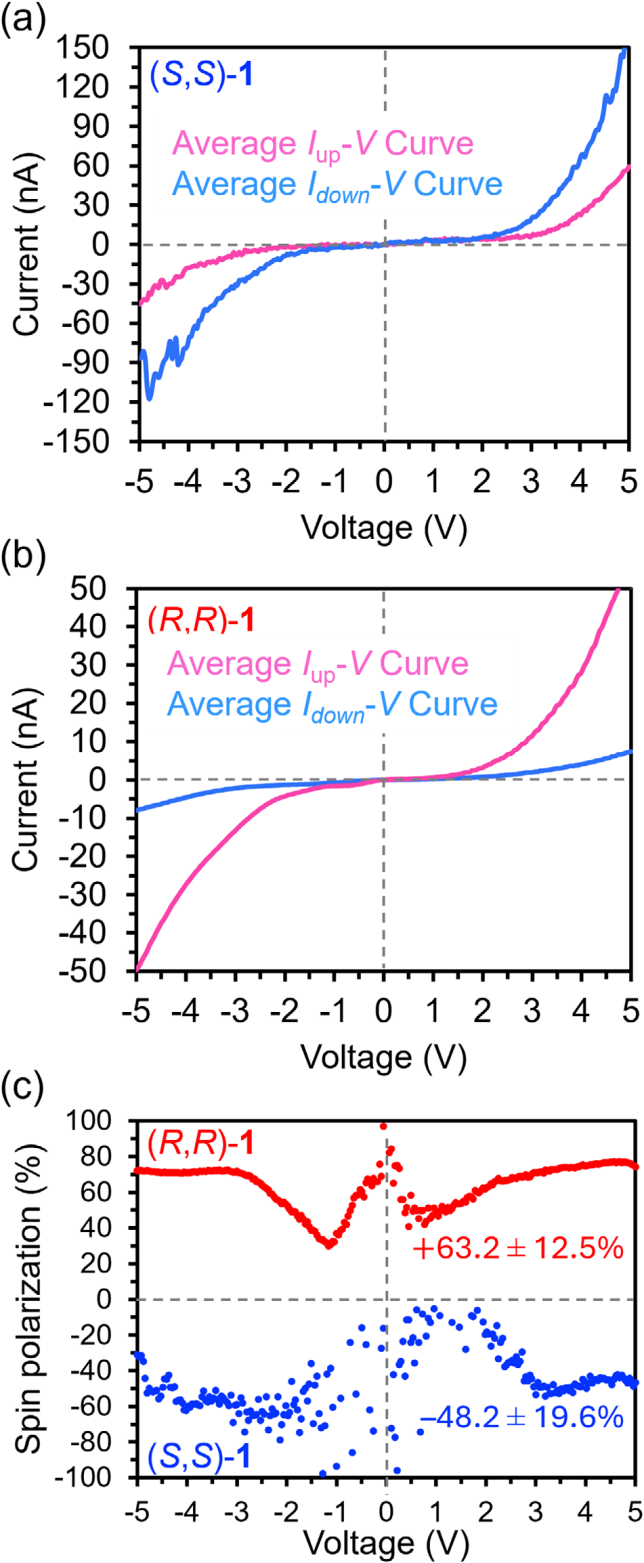
Up (pink) or down (sky blue) spin‐polarized current‐voltage curves of the thin films of (a) (*S*,*S*)‐**1** and (b) (*R*,*R*)‐**1** on HOPG. (c) Spin polarization as a function of the applied voltage for (*S*,*S*)‐**1** and (*R*,*R*)‐**1** thin films.

To put these findings into perspective, we compared the sign and magnitude of SP with our earlier chiral bifacial IDT‐based derivatives, *i.e*., π‐conjugated polymers [12] and NFAs [13]. Notably, the SP polarity is consistently tied to the absolute configuration of the IDT core: (*S*,*S*) enantiomers yield negative SP (*I*_down_ > *I*_up_), whereas (*R*,*R*) enantiomers yield positive SP (*I*_up_ > *I*_down_). This correspondence persists across amorphous or near‐amorphous thin films of π‐conjugated polymers, NFAs, and HTMs (Figure 1b–d), suggesting that the intrinsic handedness of the bifacial IDT framework biases spin‐transport polarity. While CISS has been measured across diverse molecular families, the sign of the spin polarization typically correlates with the helicity of the molecular or supramolecular architecture [27, 28, 29, 30, 31, 32]. By contrast, point chirality alone, when not coupled to a helical motif, appears to exert only a minor influence on CISS. Yamamoto and Suda measured CISS in chiral rotary molecular motors and found that, despite the presence of point chirality, the sign of the spin polarization (positive ↔ negative) switched only upon inversion of the helical sense (*M* ↔ *P*), indicating that helicity, rather than point chirality, governs the sign of spin selectivity [29, 30]. Meijer and Naaman showed that a coronene bisimide bearing (*S*)‐configured, point‐chiral side chains forms supramolecular assemblies with *P*‐helicity at 20°C and *M*‐helicity at −10°C, exhibiting up‐ and down‐spin preference, respectively [31]. This temperature‐induced helicity inversion, despite unchanged point chirality, underscores that helicity—rather than point chirality alone—controls the CISS sign. By comparison, our chiral bifacial IDT platform, which embeds point chirality in the core, shows a robust correspondence between absolute configuration and SP sign [(*S*,*S*) → negative; (*R*,*R*) → positive] across polymers, NFAs, and HTMs (Figure 1b–d), in amorphous/near‐amorphous films. We show that when point chirality is the sole chiral source, amorphous films lacking supramolecular helicity and extrinsic perturbations exhibit a CISS spin‐polarization sign that follows the molecule's absolute configuration. Recent mechanistic discussions of the CISS effect have suggested that electronic polarizability may play an important role in spin‐selective transport through chiral systems [33]. In this context, our previously reported D–A polymers [12] and A–D–A NFAs [13] possess pronounced push–pull electronic structures with strong intramolecular charge‐transfer character, which may give rise to more polarizable electronic structures. By contrast, the present HTMs can be regarded as donor‐rich D–D–D‐type molecules composed of electron‐rich TPA and IDT units. Such differences in the polarizable electronic environment may be related to the relatively moderate CISS response of the present HTMs (Figure 1b–d).

### 2.4 Photovoltaic Performance

To evaluate the photovoltaic performance, PSCs were fabricated using a 1.5 m precursor of FA_0.85_MA_0.1_Cs_0.05_PbI_3_ as the active layer in a conventional architecture of glass/FTO/compact TiO_2_/mpTiO_2_/PVK (w/wo) **1**/doped spiro‐OMeTAD/Au, as detailed in the experimental section. The **1**s layers were prepared by spin coating their 3 mg mL^−1^ CB solution at 4500 rpm. The current density‐voltage (*J*–*V*) curves (dotted lines are forward scans and solid lines are reverse scans) of the best‐performing PSCs are shown in Figure 5a, and the averaged values of multiple devices are listed in Table 2. Chiral bifacial (*R*,*R*)‐**1**‐passivated PSCs exhibited the highest short‐circuit current density (*J*_SC_) values, as confirmed by the external quantum efficiency (EQE) spectra (Figure 5b). This trend is also supported by the device resistance analysis (Table S1), in which the (*R*,*R*)‐**1**‐passivated devices showed a lower series resistance and a higher shunt resistance than the control devices. These results are consistent with improved charge extraction/transport and suppressed leakage pathways, in agreement with the hole‐extraction behavior discussed below. It should be noted that recent studies [34, 35] suggest that CISS can act as a spin‐polarizing process rather than a simple lossy spin filter, and therefore the SP value does not directly represent carrier loss in operating PSCs. PSCs passivated with **1**s exhibit an enhanced *V*_OC_, which can be reasonably attributed to the effective suppression of defect‐assisted recombination at the PVK surface. Notably, the homochiral bifacial (*R*,*R*)‐**1**, deliver the most significant improvement in *V*_OC_ (by 0.023 V compared to the control), indicating that homochiral bifacial **1**s act as a more efficient passivator. This observation is in line with the preceding discussion (theoretical calculation session), reinforcing the conclusion that chiral bifacial **1**s provide superior passivation effectiveness. Owing to the effective passivation of surface defects and the facilitated hole transport from the PVK layer to the HTL, PSCs treated with **1**s exhibited higher FF values compared to those just treated with CB. The overall enhancements in *J*_SC_, *V*_OC_, and FF ultimately yield the highest PCE of 20.64% in PSCs passivated with the chiral bifacial (*R*,*R*)‐**1**. In contrast, devices passivated by CB, *meso*‐**1**, and *rac*‐**1** exhibit lower PCEs of 19.48%, 19.71%, and 20.21%, respectively. The comparable performance of (*S*,*S*)‐**1**‐ and (*R*,*R*)‐**1**‐treated devices (Figure S11 and Table S2) indicates that the beneficial effect of homochiral bifacial passivation is not limited to a specific enantiomer. Given the nearly identical optical absorption spectra and energy levels of *meso*‐**1**, *rac*‐**1**, and (*R*,*R*)‐**1**, the performance differences are mainly attributed to interfacial passivation and hole extraction rather than intrinsic optical properties. The stability of the PSCs was also measured at ambient conditions, but showed no distinct difference among them (a decrease to ∼70% of the initial PCEs after 45 days, Figure S10 and Table S3). This is likely because of the hydrophilic nature of oligo(ethylene glycol) side chains, which facilitates the absorption of ambient moisture. Nonetheless, the superior performance of (*R*,*R*)‐**1**‐processed device provides strong evidence that the chiral bifacial molecular design strategy is an effective approach for developing passivation molecules in PSCs. The concurrent increases in *V*_OC_, FF, and *J*_SC_ and reduced hysteresis are consistent with suppressed interfacial recombination and more efficient hole extraction by the homochiral passivation layer; as reported by Ran and Chen et al. [23], a CISS‐derived spin‐selective transport may also contribute.

**FIGURE 5:**
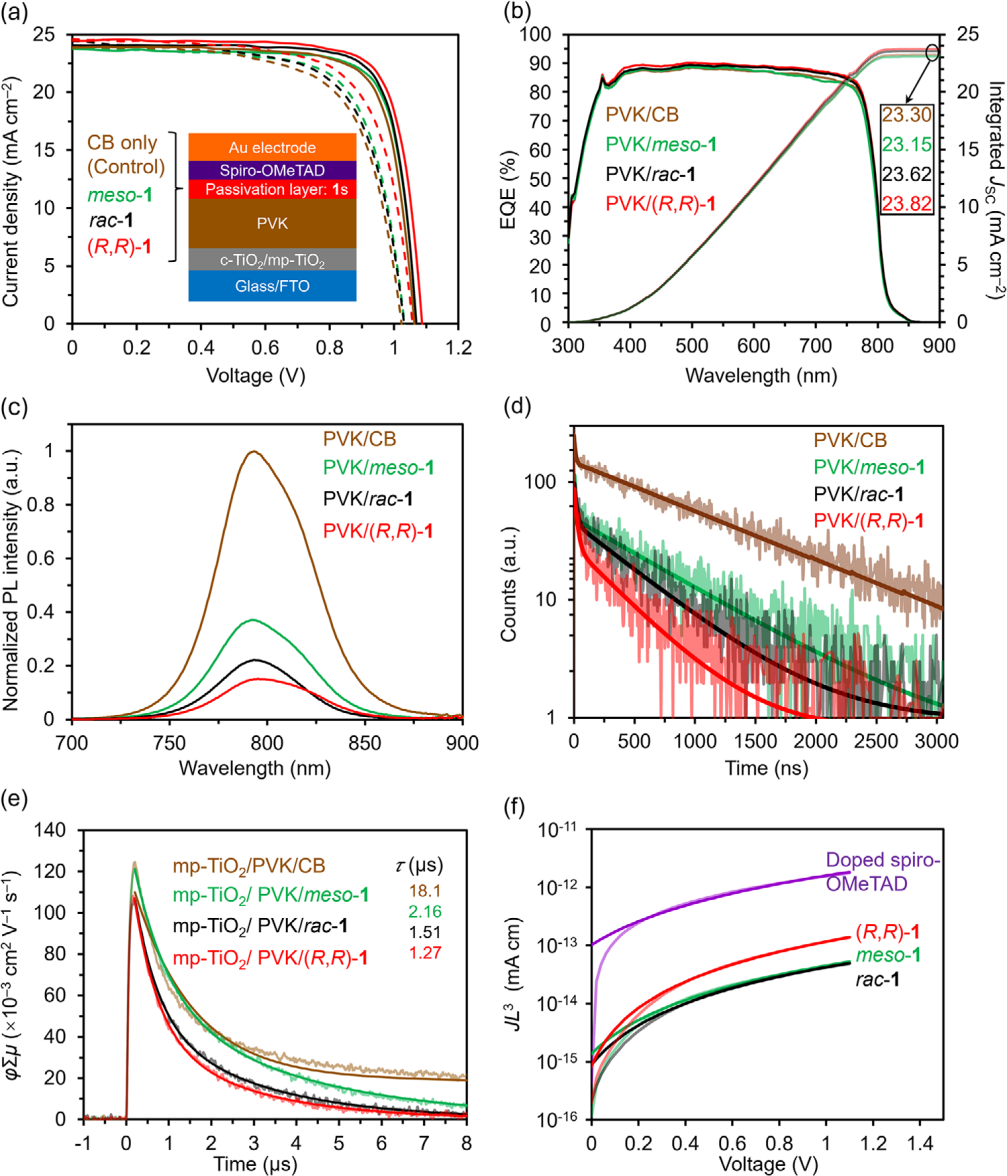
(a) Best‐performing *J*‐*V* curves of control (brown), *meso*‐**1**‐treated (green), *rac*‐**1**‐treated (black), and (*R*,*R*)‐**1**‐treated (red) PSCs (FTO/cTiO_2_/mpTiO_2_/PVK (FA_0.85_MA_0.1_Cs_0.05_PbI_3_) w/wo **1** treatment/doped spiro‐OMeTAD/Au). The dotted and solid lines are forward and reverse scans, respectively. (b) Corresponding EQE spectra of best‐performing PSCs devices. (c) PL spectra (*λ*_ex_ = 485 nm). (d) TRPL decays of PVK with/without *meso*‐**1**, *rac*‐**1** or (*R*,*R*)‐**1** passivation. (*λ*_ex_ = 450 nm, *λ*_em_ = 790 nm). (e) TRMC transients (*λ*_ex_ = 500 nm). The solid thick lines are double‐exponential fitting curves. (f) SCLC curves of doped spiro‐OMeTAD, *meso*‐**1**, *rac*‐**1** and (*R*,*R*)‐**1** in hole‐only devices (ITO/PEDOT:PSS/molecules/MoO_x_/Ag). The thick lines are fitting curves.

**TABLE 2:** Summary of PSC performances.

Active layers^a^	*J*_SC_ [mA cm^−2^]	*J*_SC_ (EQE)^b^ [mA cm^−2^]	*V*_OC_ [V]	FF [%]	HI^c^	PCE [%]	*n* of devices
Control^d^	23.72 ± 0.34 (23.85)	23.30	1.029 ± 0.01 (1.062)	69.6 ± 2 (76.9)	0.18 ± 0.02 (0.16)	18.70 ± 0.57 (19.48)	10
*meso*‐**1**	23.62 ± 0.24 (23.78)	23.15	1.042 ± 0.01 (1.067)	71.6 ± 2 (77.7)	0.15 ± 0.02 (0.12)	19.08 ± 0.58 (19.71)	12
*rac*‐**1**	23.73 ± 0.64 (24.08)	23.62	1.035 ± 0.02 (1.067)	71.9 ± 2 (78.7)	0.17 ± 0.03 (0.17)	19.32 ± 0.78 (20.21)	25
(*R*,*R*)‐**1**	23.89 ± 0.33 (24.45)	23.82	1.052 ± 0.01 (1.084)	71.5 ± 1 (77.9)	0.16 ± 0.02 (0.13)	19.56 ± 0.57 (20.64)	17

^a^ FTO/cTiO_2_/mpTiO_2_/PVK/(passivation)/doped Spiro‐OMeTAD/Au. The values in parentheses are values of the best‐performing cell in the reverse scan. The average *J*_SC_, *V*_OC_, and FF values were calculated over the forward and reverse scans of the multiple devices. The average PCE values were calculated over the reverse scans of the multiple devices

^b^ Integrated *J*_SC_ values from the EQE spectra

^c^ Hysteresis index = (reverse PCE − forward PCE)/(reverse PCE)

^d^ A control sample. Note that neat CB treatment was applied.

### 2.5 Hole Extraction and Transport Properties

Besides the function of surface passivation, hole transporting properties of **1**s are also crucial for improving PSCs performance. With this concern in mind, we systematically investigated the hole extraction and hole mobility of *meso*‐**1**, *rac*‐**1,** and (*R*,*R*)‐**1** by steady‐state photoluminescence (PL), time‐resolved photoluminescence (TRPL), time‐resolved microwave conductivity (TRMC), and space‐charge‐limited‐current (SCLC).

In contrast to typical wide‐bandgap molecular passivation, which generally increases the PL intensity by passivating trap states, *meso*‐**1**, *rac*‐**1**, and (*R*,*R*)‐**1** led to reductions in the PL intensity of PVK to 37%, 22%, and 15%, respectively, with (*R*,*R*)‐**1** exhibiting the most pronounced quenching effect (Figure 5c). Given the energy diagram of the PVK and **1**s (Figure 3c), the observed PL quenching is rationally attributed to the major effect of hole transfer from PVK to **1**s layers. From this perspective, homochiral bifacial (*R*,*R*)‐**1** is the best facilitator of hole transfer, which is possibly due to the superior steric interaction, CISS effect [23], and hole mobility (vide infra), rather than the energy‐level matching (HOMOs of **1**s are very similar). The same quenching behavior was confirmed by TRPL spectroscopy (Figure 5d), where the decay of PVK (1027 ns) was accelerated in PVK/*meso*‐**1** (696 ns), PVK/*rac*‐**1** (498 ns), and PVK/(*R*,*R*)‐**1** (352 ns) (Table 3). The decreased photoluminescence quantum yield (PLQY) (Figure S12) further emphasizes the PL quenching effect of **1**s.

**TABLE 3:** Averaged lifetimes (*T*_ave_) of TRPL analysis of HTM‐passivated PVK and hole mobilities of HTMs by SCLC.

TRPL Samples	*T*_ave_ [ns]^a^		SCLC Samples	*µ*_h_ [cm^2^ V^−1^ s^−1^]^b^
Control	1027		Doped spiro‐OMeTAD	2.2 × 10^−3^
*meso*‐**1**	696		*meso*‐**1**	7.7 × 10^−5^
*rac*‐**1**	498		*rac*‐**1**	7.7 × 10^−5^
(*R*,*R*)‐**1**	352		(*R*,*R*)‐**1**	2.4 × 10^−4^

^a^ See the Table S4 for details of fitting

^b^ Hole only device structure: glass/ITO/PEDOT:PSS/HTMs/MoO_3_/Ag.

We performed TRMC [36, 37] measurements for electron transport layer (ETL: mpTiO_2_)/PVK w/wo **1**s (Figure 5e). The long‐lived TRMC decays (*φ* is the charge carrier generation yield, and ∑*µ* is the sum of the hole and electron local mobilities), originating from holes in PVK owing to the prompt electron transfer to ETL, were progressively accelerated by the treatment of *meso*‐**1**, *rac*‐**1**, and (*R*,*R*)‐**1**. The effective holes lifetime (*τ*) decreased from 18.1 to 2.16 µs, 1.51 µs, and 1.27 µs for the *meso*‐**1**, *rac*‐**1**, and (*R*,*R*)‐**1** treated samples, respectively. These results clearly demonstrate that hole transfer from PVK to the **1** layer is more efficient in the homochiral (*R*,*R*)‐**1** than in the *meso*‐**1** and *rac*‐**1** cases. To clarify the universality of the hole‐extraction ability of *meso*‐**1**, *rac*‐**1**, and (*R*,*R*)‐**1**, PL and TRMC measurements were additionally performed using another PVK composition of MA_0.13_FA_0.87_PbI_2.61_Br_0.39_. As shown in Figure S13, these results are consistent with the conclusions obtained above.

The hole mobilities of *meso*‐**1**, *rac*‐**1**, and homochiral (*R*,*R*)‐**1** films were evaluated using hole‐only SCLC devices (ITO/PEDOT:PSS/**1**s layer (116–138 nm)/MoO_3_/Ag). The measured values for *meso*‐**1** and *rac*‐**1** are 7.7 × 10^−^^5^ and 7.7 × 10^−^^5^ cm^2^ V^−^^1^ s^−^^1^, respectively, whereas the (*R*,*R*)‐**1** film exhibited a much higher hole mobility of 2.4 × 10^−^^4^ cm^2^ V^−^^1^ s^−^^1^, nearly three times greater than those of its achiral and racemic counterparts (Figure 5f and Table 3). This enhanced mobility in the chiral bifacial (*R*,*R*)‐**1** film is consistent with its stronger PL quenching, shorter PL lifetime, and faster TRMC decay. Although not conclusive, this mobility increase may reflect a CISS‐assisted transport mechanism [5]. In chiral media, coupling between carrier spin and velocity is expected to impede backscattering and thereby increase the efficiency of electron/hole transfer, whereas racemic mixtures can partially cancel spin selectivity. Thus, while this interpretation is plausible and aligns with recent reviews on CISS‐driven transport, we refrain from a definitive claim because macroscopic mobility can also be influenced by film packing and interfacial states [5]. Nevertheless, in actual PSC devices, the **1** layer is ultra‐thin, and its hole mobilities are still considerably lower than that of doped spiro‐OMeTAD (2.2 × 10^−^^3^ cm^2^ V^−^^1^ s^−^^1^). This likely explains the relatively minor differences in *J*_SC_ between PSCs treated with chiral bifacial (*R*,*R*)‐**1** and those treated with *meso*‐ and *rac*‐**1**s.

The homochiral (*R*,*R*)‐**1**‐based system exhibits pronounced PL quenching, shortened PL lifetimes, accelerated TRMC decay, and increased SCLC hole mobility. These observations indicate more efficient hole extraction and are consistent with the findings of Ran and Chen [23]. Taken together, these results suggest that homochiral passivation layers can support more efficient interfacial hole extraction in PSCs, while CISS‐assisted transport may also contribute, underscoring homochirality as a practical design lever for interfacial engineering [22].

The interactions between **1**s and the PVK surface were first investigated by X‐ray photoelectron spectroscopy (XPS), as shown in Figure 6. Upon passivation, the Pb 4f and I 3d peaks of **1**‐treated PVK films shifted toward higher binding energies compared with those of the CB‐treated film, which indicates the change of chemical environments of Pb^2+^ and I^−^ [38, 39]. Chemical‐state deconvolution of the Pb 4f spectra showed that the signals were dominated by Pb^2^^+^ components with a minor metallic Pb^0^ contribution. Notably, quantitative deconvolution of Pb 4f revealed that the Pb^0^ content was suppressed from 0.7% in the control and *meso*‐**1**‐treated samples to 0.2% in the *rac*‐**1**‐ and (*R*,*R*)‐**1**‐treated films, supporting the efficacy of these bifacial **1**s [*rac*‐**1** and (*R*,*R*)‐**1**] in inhibiting Pb^0^‐related defect formation. Given the XPS profiles of N 1s and O 1s in Figure S14a,b, the binding energies of N1s and O1s are shifted to higher values, indicating the O atoms of oligo(ethylene glycol) side chains contribute to PVK surface passivation in addition to N atoms of TPA groups. This is likely responsible for more efficient passivation of bifacial **1**s [*rac*‐**1** and (*R*,*R*)‐**1**] than *meso*‐**1** because two oligo(ethylene glycol) side chains of bifacial **1**s can contact PVK surface simultaneously. The N/O→Pb coordination is further supported by ^1^H NMR characterization. Upon addition of 4 equivalents of PbI_2_ to solutions of the **1**s in DMSO‐*d*_6_, the signals corresponding to the aromatic protons near the N atoms of the TPA groups and the methylene protons on the oligo(ethylene glycol) side chains showed upfield shifts (Figure S15). Transient photovoltage (TPV) (Figure S16a) and light‐intensity‐dependent *V*_OC_ (Figure S16b) measurements were performed to evaluate the practical passivation efficacy. The prolonged charge carrier lifetime in TPV and the reduced ideality factor (*n*) extracted from *V*_OC_ vs. light intensity plots collectively confirm that *meso*‐**1**, *rac*‐**1**, and (*R*,*R*)‐**1** treatments effectively suppress non‐radiative recombination at the perovskite interface.

**FIGURE 6:**
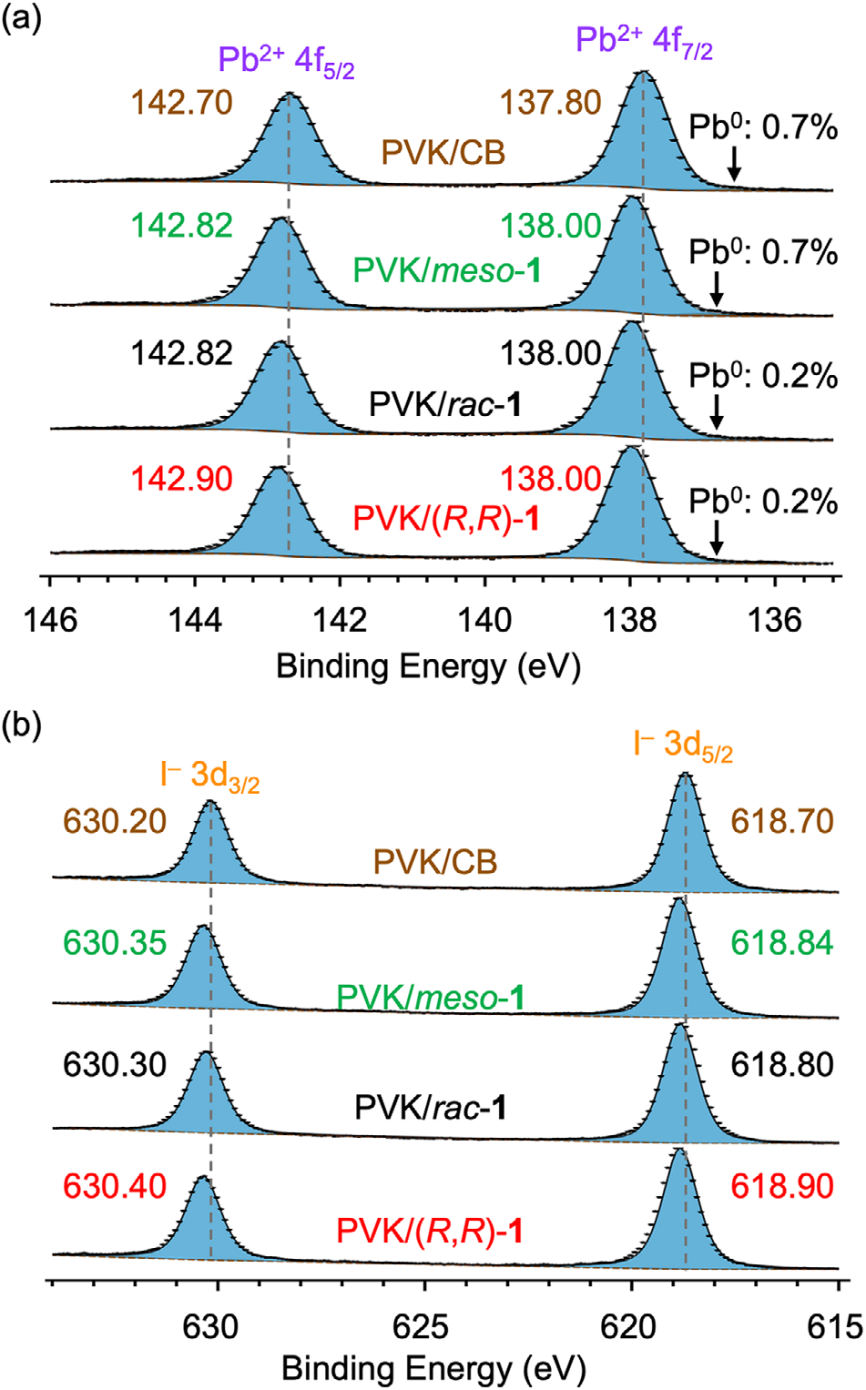
X‐ray photoelectron spectra at (a) Pb 4f and (b) I 3d with/without *meso*‐**1**, *rac*‐**1** or (*R,R*)‐**1** passivation.

The orientation and multicrystalline structures of PVK with and without **1**s were investigated by two‐dimensional grazing‐incidence X‐ray diffraction (2D‐GIXRD). As shown in Figure 7a–d, all the samples yield arc‐shaped diffractions. The clear diffraction at *q*_z_ = 9.1 nm^−1^ in the out‐of‐plane (OOP) direction is attributed to PbI_2_ due to an excessive PbI_2_ component in the PVK precursor, which has a positive effect on the PSCs performance [40]. The presence of PbI_2_ on the PVK surface can also be intuitively observed in scanning electron microscope (SEM) images (a long shape with a less conductive bright color in Figure S17) and suggested by the 2D‐GIXRD profile (Figure S18) and powder XRD patterns (Figure S19). The other diffractions in the OOP direction of *q*_z_ = 10.2, 14.1, 17.2, 20.1, and 22.1 nm^−1^ are assigned to (001), (011), (111), (002), and (012) of the cubic phase (α‐phase) of FAPbI_3_ [41]. After **1**s treatments, the amount of residual PbI_2_ on the PVK surface slightly decreases, as evidenced by the reduced ratio of PbI_2_ to perovskite (001) diffraction intensities in the OOP direction. However, no obvious difference was observed among *meso*‐**1**, *rac*‐**1**, and chiral (*R*,*R*)‐**1**‐treated PVK surfaces, which is different from the previous reports [15]. Notably, this is a surface phenomenon because it can only be observed in surface‐sensitive 2D‐GIXRD measurements, but not in bulk‐sensitive normal XRD measurements.

**FIGURE 7:**
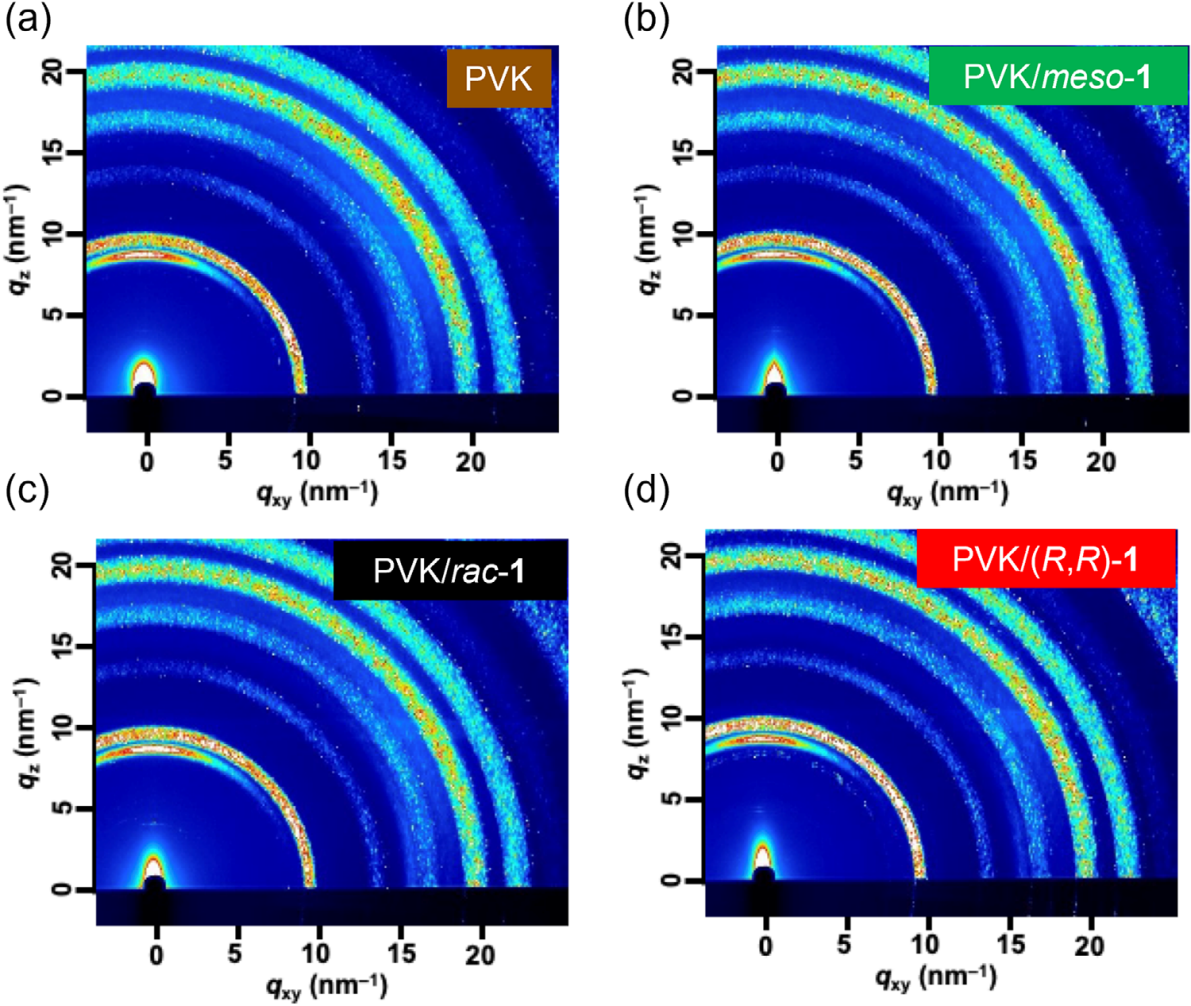
2D‐GIXRD images of (a) CB‐treated PVK, (b) *meso*‐**1**‐treated PVK, (c) *rac*‐**1**‐treated PVK and (d) (*R*,*R*)‐**1**‐treated PVK.

## 3 Conclusion

We designed and synthesized a family of chiral bifacial HTMs [(*S*,*S*)‐ and (*R*,*R*)‐**1**] built on a bifacial IDT core bearing hydrophilic oligo(ethylene glycol) and hydrophobic phenyl substituents. Spin‐polarized conductive AFM revealed pronounced chirality‐induced spin selectivity (CISS) in amorphous films of the enantiomers, with spin polarization (SP) ≈60%. Importantly, across our broader chiral‐bifacial IDT platform—π‐conjugated polymers, NFAs, and the present HTMs—we observe a consistent chirality–spin polarity correspondence: (*S*,*S*) yields negative SP whereas (*R*,*R*) yields positive SP, even in amorphous/near‐amorphous films. To our knowledge, this constitutes the first systematic demonstration of a configuration‐defined spin‐polarity map on a unified molecular backbone spanning three molecular classes.

In perovskite solar cells, ultrathin layers of these HTMs served as effective passivation agents. Devices passivated with homochiral (*R*,*R*)‐**1** exhibited higher *V*_OC_, improved FF, and increased PCE relative to *meso* and racemic controls, consistent with suppressed interfacial recombination and more efficient hole extraction. In addition, SCLC measurements indicated ∼3× higher hole mobility for the homochiral film relative to the racemate; although not conclusive, this trend is consistent with a CISS‐assisted transport contribution [5]. While a direct causal link to interfacial CISS remains to be fully established, the convergence of photophysical (PL/TRPL), transport (TRMC/SCLC), and photovoltaic metrics is consistent with a scenario in which homochiral passivation promotes favorable interfacial and CISS‐assisted, spin‐selective transport [23], thereby aiding hole extraction and suppressing interfacial recombination. Taken together, these results establish a generalizable guideline—embedding point chirality in a bifacial IDT framework to program spin‐transport polarity—and highlight homochiral, CISS‐active passivation as a practical lever for advancing perovskite optoelectronics [22].

## Conflicts of Interest

The authors declare no conflicts of interest.

## Supporting information




**Supporting File**: smll75074‐sup‐0001‐SuppMat.pdf.

## Data Availability

The data that support the findings of this study are available in the supplementary material of this article.
