# Supplementary material for: Chiral Bifacial Indacenodithiophene‐Based Hole‐Transport Materials With Chirality‐Induced Spin Selectivity: Chirality‐Spin Polarity Correspondence and Perovskite Passivation
Source: Small. 2026 Aug 8;22(57):e75074. doi: 10.1002/smll.75074 (PMC13659782; doi:10.1002/smll.75074)
Supplement: Supplementary file 1 [file SMLL-22-e75074-s001.pdf]

Supporting Information

Chiral Bifacial Indacenodithiophene-Based Hole-Transport Materials with Chirality-Induced Spin Selectivity: Chirality-Spin Polarity Correspondence and Perovskite Passivation

Shuang Li,[a] Fumitaka Ishiwari,*[a,b,c,d] Ryosuke Nishikubo,[a, b] and Akinori Saeki*[a,b]

[a] Department of Applied Chemistry, Graduate School of Engineering, Osaka University, 2-1 Yamadaoka, Suita, Osaka 565-0871, Japan.

[b] Innovative Catalysis Science Division, Institute for Open and Transdisciplinary Research Initiatives (ICS-OTRI), Osaka University, 1-1 Yamadaoka, Suita, Osaka 565-0871, Japan.

[c] PRESTO, Japan Science and Technology Agency (JST), 4-1-8 Honcho, Kawaguchi, Saitama 332-0012, Japan.

[d] Graduate School of Urban Environmental Sciences, Tokyo Metropolitan University, 1-1 Minamiosawa, Hachioji, Tokyo 192-0297, Japan.

*To whom correspondence should be addressed.
E-mail: ishiwari@chem.eng.osaka-u.ac.jp (F.I.)
saeki@chem.eng.osaka-u.ac.jp (A.S.)

Table of Contents

1. Materials...S2
2. General...S2
3. Theoretical Calculations..S3
4. CISS Measurement..S3
5. PSC and SCLC Device Fabrication and Characterization............................S3
6. Synthesis...S4
7. Supporting Figures (Figures S1–S19)...S7
8. Analytical Data (Figures S20–S35)...S18
9. Supporting Tables (Tables S1–S7)..S26
10. Supporting References..S37

1. Materials.

Unless otherwise stated, all commercial reagents were used as received. Compounds *rac*-**2**[S1], (*S*,*S*)-**2**[S1], (*R*,*R*)-**2**[S1] and *meso*-**2**[S2] were prepared according to previously reported procedures[S1] and unambiguously characterized by NMR spectroscopy and matrix assisted laser desorption/ionization time of flight (MALDI-TOF) mass spectrometry.

2. General.

NMR spectroscopy measurements in solution were carried out on a JEOL JNM-ECS400 spectrometer (400 MHz for ^{1}H and 100 MHz for ^{13}C). Chemical shifts (δ) are expressed relative to the resonances of the residual non-deuterated solvents for ^{1}H [DMSO-d_6: ^{1}H(δ) = 2.50 ppm] and ^{13}C [DMSO-d_6: ^{13}C(δ) = 39.52 ppm]. Absolute values of the coupling constants are given in Hertz (Hz), regardless of their sign. Multiplicities are abbreviated as singlet (s), doublet (d), triplet (t) and multiplet (m). Infrared (IR) spectra were recorded at 25 °C on a JASCO model FT/IR-4700AC Fourier-transform infrared spectrometer. High-resolution matrix assisted laser desorption/ionization time of flight (MALDI-TOF) mass spectrometry measurements were performed on a JEOL model JMS-S3000 mass spectrometer. UV-vis absorption spectra were recorded at 25 °C on a JASCO model Jasco V-730 UV-vis spectrophotometer. CD spectra were recorded on a JASCO J-820 spectropolarimeter. Thermogravimetric analysis (TGA) was performed on a SHIMADZU model TGA-50 analyzer. Atomic force microscopy (AFM) measurements of the thin-films of the (*S*,*S*)-**1** and (*R*,*R*)-**1** were performed on a Bruker Innova atomic force microscope operated in tapping mode using a silicon cantilever tip (a Bruker OLTESPA-R3). 2D GI-XRD on the BL13XU beamline at SPring-8 (Japan Synchrotron Radiation Research Institute, JASRI), using 12.39 keV (λ = 1 Å) X-rays were performed at the grazing incidence angle of 0.12°. 2D diffraction patterns were recorded with a 2D detector, Dectris PILATUS 300K. X-ray diffraction (XRD) measurements were performed using a Rigaku Corp. MiniFlex-600 instrument (Cu Kα radiation: λ = 1.54 Å). SEM measurements were performed on a JEOL JSM-IT700HR (5 keV, 50 µA). Photoelectron yield spectroscopy (PYS) measurement was carried out using a Bunko Keiki BIP-KV202GD measurement system. The samples were prepared by spin coating the chlorobenzene solution of the molecule onto a glass/ITO substrate. The measurement energy range was 4.0–8.0 eV. PL measurement was carried out using a spectrofluorometer (JASCO FP-8300). The excitation wavelength was 485 nm. Time-resolved photoluminescence lifetime (TRPL) measurements were conducted on an Edinburgh Instruments FS5 v2 spectrofluorometer. The excitation wavelength was 450 nm. Photoluminescence quantum yield (PLQY) measurements were performed on a Hamamatsu Photonics Quantaurus-QY Plus C13534-21 using a continuous wave laser at 375 nm (Spectra Physics Corp. EXLSR-375C-70S-W). Flash-photolysis time-resolved microwave conductivity (TRMC) measurements were performed for the samples on a quartz plate. The excitation laser from an optical parametric oscillator (OPO, Continuum Inc., Panther) seeded by the third harmonic generation (355 nm) of a Nd:YAG laser (Continuum Inc., Surelite II, 5–8 ns pulse duration, 10 Hz) was set to 500 nm at $I_0 = 3.8 \times 10^{10}$ photons cm^{-2} pulse^{-1}. The laser pulse was exposed from the top side (not from the quartz side). The photoconductivity transient $\Delta\sigma$ was converted to the product of the quantum efficiency (φ) and the sum of the charge carrier mobilities, $\Sigma\mu$ (= $\mu_h + \mu_e$) by $\varphi\Sigma\mu = \Delta\sigma (eI_0F_{light})^{-1}$, where e and F_{light} are the unit charge of a single electron and a correction (or filling) factor, respectively. Transient photovoltage (TPV)

and transient photocurrent (TPC) measurements were conducted by using a second harmonic generation (SHG) at 532 nm from a Spectra Physics Inc., GCR-100 (5–8 ns pulse duration, 10 Hz). TPV and TPC were carried out at open-circuit (1 MΩ termination) and short-circuit (50 Ω termination) under white light LED illumination. All the experiments were conducted at room temperature in the air.

3. Theoretical Calculation.

Theoretical calculations were conducted using DFT and TD-DFT methods, respectively with B3LYP/6-31G(d,p) level of theory at S1 states, using Gaussian 16 program package.[S3] To simplify the calculations, we calculated simplified molecules of *meso*-**1**, (*S*,*S*)-**1** and (*R*,*R*)-**1**, where their tetraethylene glycoloxy moieties were replaced by methoxy. (Tables S3–5). The optimized structure of (*R*,*R*)-**1** molecule was obtained from mirror inversion of optimized (*S*,*S*)-**1** molecule. Optimized geometries were verified by frequency computations as minima (no imaginary frequencies). The theoretically calculated results were visualized using Multiwfn [S4] and VMD softwares.

4. Chirality induced spin selectivity (CISS).

CISS measurements were performed using the same methodology as described in our previous study.[S1] Samples were fabricated by spin coating 5 mg mL^{-1} (*S*,*S*)-**1** and (*R*,*R*)-**1** chlorobenzene solution onto highly oriented pyrolytic graphite (HOPG) at 1500 rpm, followed by annealing at 100 °C for 10 min. Spin-polarized conductive-AFM measurements were performed on a Bruker Innova atomic force microscope equipped with a nonmagnetic sample holder (a Bruker APSH-0020) and a CoCr-cantilever (a Bruker MESP-V2). Disc-shaped Nd magnet was put with up or down magnetization directions on the CoCr-cantilever at a distance of ca. 6 mm and measured the current-voltage (*I*-*V*) curve in contact with the surface. We measured more than 100 *I*-*V* curves at different locations for each sample. HOPG substrates were purchased from Bruker (ZYB grade) and freshly cleaved just before use. The cantilever was replaced when changing thin films to keep using a fresh cantilever. The film thicknesses on HOPG were determined using the same AFM facility operated in tapping mode with a silicon cantilever tip (Bruker OLTESPA-R3), by measuring the step height of partially scratched films.

5. PSC and SCLC Device Fabrication and Characterization.

An FTO layer on a glass substrate was etched with 6 mol dm^{-3} HCl and Zn using masking tape. After sequential sonication in detergent, deionized water, acetone, and isopropyl alcohol, followed by O_3/UV treatment for 15 min at each step, a compact TiO_2 (c-TiO_2) layer was deposited onto the FTO/glass substrate by spray pyrolysis using a solution of titaniumdiisopropoxide bis(acetylacetonate) (Tokyo Chemical Industry Co.Ltd., TCI) in ethanol (1:14 v/v) at 450 °C. A 200 nm thick mesoporous TiO_2 (mpTiO_2) layer (average particle size: 30 nm, anatase) was deposited onto the cTiO_2 layer by spin-coating (slope 3s, 5000 rpm for 15 s, slope 2 s) of a diluted TiO_2 paste (PST−30NR-D, GreatCellSolar Ltd.) in ethanol (paste:ethanol = 1:7 w/w), followed by sintering at 500 °C for 20 min. A 1.5 M *N*,*N′*-dimethylformamide (DMF, super dehydrated, Wako):dimethylsulfoxide (DMSO, super dehydrated, Wako) = 4:1 (v/v%) solution at the stoichiometry of $FA_{0.85}MA_{0.1}Cs_{0.05}PbI_3$ with 10

mol% exccess PbI_2 was prepared. MACl (GreatCellSolar Ltd.) 12.66 mg (15 mol%), FAI (GreatCellSolar Ltd.) 219.26 mg (85 mol%), MAI (GreatCellSolar Ltd.) 23.85 mg (10 mol%), PbI_2 (Tokyo Chemical Industry Co.Ltd., TCI) 760.67 mg (110 mol%), CsI (GreatCellSolar Ltd.) 19.49 mg (5 mol%), and $PbCl_2$ (Tokyo Chemical Industry Co.Ltd., TCI) 20.86 mg (5 mol%) were dissolved in a 1 mL mixture solution in an N_2-filled glovebox. Subsequently, a perovskite layer was formed by spin-coating the solution (slope 2 s, 1000 rpm for 10 s, slope of 2 s, 5000 rpm for 40 s, slope of 2 s). At 51s, poor-solvent treatment (chlorobenzene, anhydrous 99.8%, Sigma-Aldrich) was applied by dropping 200 μL onto the rotating substrate. The resultant transparent film was annealed at 100°C for 20 min, The passivation layer was spun-cast at 4500 rpm for 30 s from a super anhydrous CB solution (3 $mg \cdot mL^{-1}$). A Spiro-OMeTAD (Tokyo Chemical Industry Co.Ltd., TCI) was deposited by spin-coating of 78.2 $mg \cdot mL^{-1}$ chlorobenzene (Sigma-Aldrich, anhydrous, 99.8%) with 52 mol% LiTFSI (TCI, TFSI is bis(trifluoromethanesulfonyl)imide) relative to Spiro-OMeTAD dissolved in acetonitrile (Sigma-Aldrich, anhydrous, 99.8%) and 2.9 vol % 4-tert-butylpyridine (TBP, Sigma-Aldrich, 98%) relative to the solution volume. Subsequently, a 70 nm thick stripe-shaped gold electrode was thermally deposited in a vacuum chamber. To promote oxygen doping of HTL, devices were stored in an oxygen-substituted desiccator in the dark for 3 days.

Current−voltage curves were measured using a source meter unit (ADCMT Corp., 6241A) under AM1.5G solar illumination at 100 $mW \cdot cm^{-2}$ (1 sun, monitored by a calibrated standard cell, Bunko Keiki BS-520BK) from a 300 W solar simulator (SAN-EI Corp., XES-301S). The size of the active area was defined by a black metal mask with a square hole (2 × 2 mm^2). External quantum efficiency (EQE) spectra were measured by a Bunko Keiki model SM-250KD equipped with a Keithley model 2401 source meter. The monochromated light power was calibrated using a silicon photovoltaic cell (Bunko Keiki model S1337-1010BQ). All the experiments were conducted at room temperature in the air.

Hole-only space-charge-limited current (SCLC) devices were fabricated. An glass/ITO substrate was washed by sonication with detergent, acetone and isopropanol for 15 min at each step and treated by O_3/ultraviolet for 15 min. Then, a PEDOT:PSS solution (300 μL) was spin-coated at 5000 rpm for 60 s and thermally annealed at 140 °C for 30 min. After that, a 50 μL chlorobenzene solution of (60 $mg \cdot mL^{-1}$ for passivation molecules or 78.4 $mg \cdot mL^{-1}$ for spiro-OMeTAD that was doped by Li-TFSI and 4-tert-butylpyridine (tBP)) was spin-coated at 4000 rpm for 15 s, followed by a thermal evaporation of 10 nm MoO_x. Finally, 80 nm Ag layer was deposited by vacuum evaporation through a shadow mask (area: 7.07 mm^2). Current−voltage curves were measured using a source meter unit (ADCMT Corp., 6241A) in darkness. The thicknesses of active layer were determined to be 164.3 nm (spiro-OMeTAD), 115.9 nm (*meso*-**1**), 129.7 nm (*rac*-**1**) and 138.0 nm ((*R*,*R*)-**1**), respectively, using a Bruker Dektak XT profilometer by measuring the step height of partially scratched films.

6. Synthesis

Synthesis of *meso*-1, *rac*-1, (*S*,*S*)-1 and (*R*,*R*)-1. Under nitrogen, *meso*-**2** (60.0 mg, 60.7 μmol, 1.0 eq), [4-[bis(4-methoxyphenyl)amino]phenyl]boronic acid (50.9 mg, 145.7 μmol, 2.4 eq), K_3PO_4 (258.6 mg, 1213 μmol, 20 eq), P(*t*-Bu)$_3$Pd G2 (6.2 mg, 12 μmol, 0.2 eq) was dissolved in degassed THF (12.0 mL) and H_2O (3.0 mL). After stirring at 25 °C for 12 h, the reaction mixture was poured into water. The organic phase was extracted using $CHCl_3$. A combined

organic extract was washed with brine, dried over anhydrous sodium sulfate, and then evaporated to dryness under reduced pressure. The residue was subjected to column chromatography on silica (ethyl acetate: hexane = 8: 2) to allow isolation of *meso*-**1** as orange solid (70.5 mg, 49.0 μmol) in 80.8% yield. By the same procedure, *rac*-**1** was synthesized using *rac*-**2** and isolated by column chromatography on silica (ethyl acetate: hexane = 8:2) as orange solid (65.5mg, 45.6 μmol) in 75.1% yield. By the same procedure, (*S*,*S*)-**1** was synthesized using (*S*,*S*)-**2** and isolated by column chromatography on silica (ethyl acetate: hexane = 8: 2) as orange solid (72 mg, 50 μmol) in 82.5% yield. By the same procedure, (*R*,*R*)-**1** was synthesized using (*R*,*R*)-**2** and isolated by column chromatography on silica (ethyl acetate: hexane = 8: 2) as orange solid (68.6 mg, 47.7 μmol) in 78.6% yield.

***meso*-1**: ^{1}H NMR (400MHz, 298 K, DMSO-d$_6$): δ (ppm) 7.48 (d, J = 7.72 Hz, 2H), 7.42 (d, J = 7.40 Hz, 2H), 7.28-7.36 (m, 5H), 7.04 (d, J = 7.08 Hz, 4H), 6.92 (d, J = 7.04 Hz, 4H), 6.75 (d, J = 7.68 Hz, 2H), 3.74 (s, 6H), 3.42-3.53 (m, 12H), 3.20-3.40 (m, 2H), 3.15 (s, 3H). ^{13}C{^{1}H} NMR (100 MHz, DMSO-d$_6$): δ (ppm) 156.14, 152.22, 151.29, 148.35, 147.98, 141.48, 140.56, 139.80, 134.96, 128.70, 127.92, 127.04, 126.13, 125.72, 125.61, 119.35, 117.59, 115.52, 115.45, 115.20, 86.17, 71.36, 69.94, 69.90, 69.78, 69.68, 63.85, 58.14, 55.42. FT-IR (KBr): v (cm^{-1}) FT-IR (KBr): v (cm^{-1}) 2931, 2877, 1649, 1506, 1456, 1240, 1116, 1097, 1031, 821. MALDI-TOF-mass: calcd. for $C_{86}H_{88}$ N_2 $O_{14}S_2$ [M]$^+$; m/z = 1436.5671; found:1436.5703. ^{1}H NMR, ^{13}C{^{1}H} NMR, FT-IR, and high-resolution MALDI-TOF mass spectra of ***meso*-1** are shown in Figures S16, S17, S18, and S19, respectively.

***rac*-1**: ^{1}H NMR (400 MHz, 298 K, DMSO-d$_6$): δ (ppm) 7.49 (d, J = 8.76 Hz, 2H), 7.37-7.39 (m, 2H), 7.26-7.32 (m, 5H), 7.01-7.05 (m, 4H), 6.91-6.94 (m, 4H), 6.75 (d, J = 8.84 Hz, 2H), 3.74 (s, 6H), 3.44-3.57 (m, 12H), 3.30-3.37 (m, 2H), 3.17 (s, 3H).
^{13}C{^{1}H} NMR (100 MHz, DMSO-d$_6$): δ (ppm) 156.00, 152.02, 151.14, 148.20, 147.84, 141.30, 140.54, 139.66, 134.73, 128.53, 127.69, 126.88, 125.99, 125.56, 119.21, 117.48, 115.04, 85.97, 71.25, 69.83, 69.68, 69.58, 63.70, 58.02, 55.27. FT-IR (KBr): v (cm^{-1}) FT-IR (KBr): v (cm^{-1}) 2912, 2888, 1637, 1608, 1504, 1456, 1240, 1135, 1110, 1032, 821. MALDI-TOF-mass: calcd. for $C_{86}H_{88}$ N_2 $O_{14}S_2$ [M]$^+$; m/z = 1436.5671; found:1436.5677. ^{1}H NMR, ^{13}C{^{1}H} NMR, FT-IR, and high-resolution MALDI-TOF mass spectra of ***rac*-1** are shown in Figures S20, S21, S22, and S23, respectively.

(*S*,*S*)-1: ^{1}H NMR (400 MHz, 298 K, DMSO-d$_6$): 7.49 (d, J = 8.52 Hz, 2H), 7.38 (d, J = 6.88 Hz, 2H), 7.26-7.32 (m, 5H), 7.03 (d, J = 8.84 Hz, 4H), 6.92 (d, J = 8.76 Hz, 4H), 6.75 (d, J = 8.60 Hz, 2H), 3.74 (s, 6H), 3.42-3.56 (m, 12H), 3.31-3.38 (m, 2H), 3.16 (s, 3H). ^{13}C{^{1}H} NMR (100 MHz, DMSO-d$_6$): δ (ppm) 156.03, 152.05, 151.18, 148.23, 147.87, 141.32, 140.57, 139.69, 134.75, 128.56, 127.74, 126.91, 126.02, 125.59, 125.52, 119.24, 117.52, 115.32, 115.07, 86.00, 71.28, 69.86, 69.71, 69.60, 63.72, 58.04, 55.30. FT-IR (KBr): v (cm^{-1}) 2912, 2860, 1650, 1604, 1504, 1458, 1240, 1117, 1097, 1036, 821. MALDI-TOF-mass: calcd. for $C_{86}H_{88}$ N_2 $O_{14}S_2$ [M]$^+$; m/z = 1436.5671; found:1436.5675. ^{1}H NMR, ^{13}C{^{1}H} NMR, FT-IR, and high-resolution MALDI-TOF mass spectra of **(*S*,*S*)-1** are shown in Figures S24, S25, S26, and S27, respectively.

(*R*,*R*)-1: ^{1}H NMR (400 MHz, 298 K, DMSO-d$_6$): 7.49 (d, J = 8.64 Hz, 2H), 7.38 (d, J = 7.20 Hz, 2H), 7.28-7.36 (m, 5H), 7.03 (d, J = 8.68 Hz, 4H), 6.92 (d, J = 8.64 Hz, 4H), 6.75 (d, J = 8.52 Hz, 2H), 3.74 (s, 6H), 3.44-3.56 (m, 12H), 3.3-3.36 (m, 2H), 3.16 (s, 3H). ^{13}C{^{1}H} NMR (100 MHz, DMSO-d$_6$): δ (ppm) 156.14, 152.22, 151.29, 148.36, 147.98, 141.48, 140.56,

139.80, 134.96, 128.70, 127.92, 127.04, 126.13, 125.72, 125.61, 119.35, 117.59, 115.51, 115.20, 86.17, 71.36, 69.94, 69.78, 69.69, 63.86, 58.14, 55.42. FT-IR (KBr): ν (cm^{-1}) 2910, 2862, 1648, 1506, 1458, 1240, 1098, 1024, 821. MALDI-TOF-mass: calcd. for $C_{86}H_{88}N_2O_{14}S_2$ $[M]^+$; *m/z* = 1436.5671; found:1436.5693. ^1H NMR, $^{13}C\{^1H\}$ NMR, FT-IR, and high-resolution MALDI-TOF mass spectra of **(*R*,*R*)-1** are shown in Figures S28, S29, S30, and S31, respectively.

7. Supporting Figures

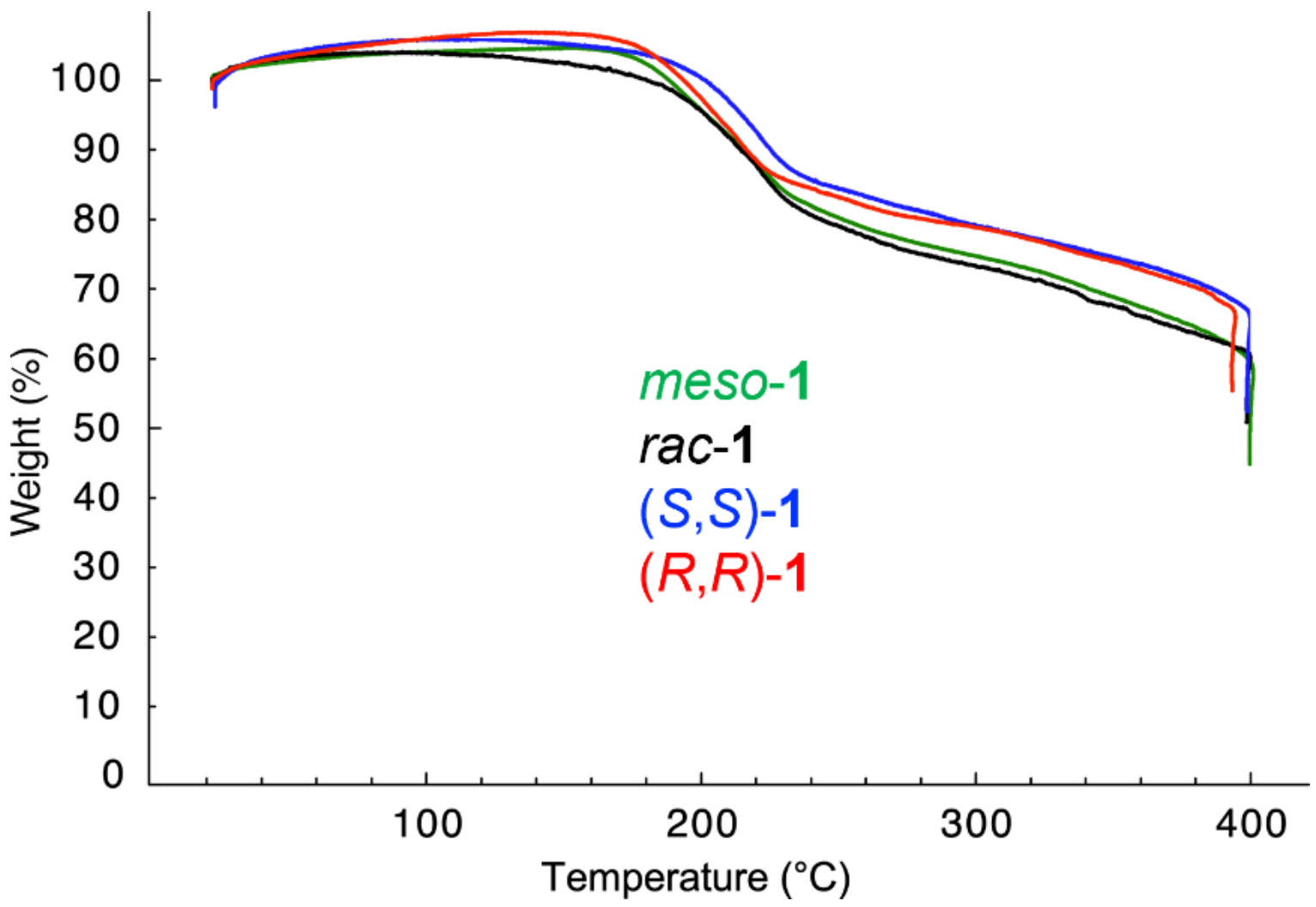

Figure S1. TGA profiles of *meso*-**1**, *rac*-**1**, (*S*,*S*)-**1**, and (*R*,*R*)-**1** measured at a scan rate of 10 °C min^{-1} under N_2 flow (50 mL min^{-1}).

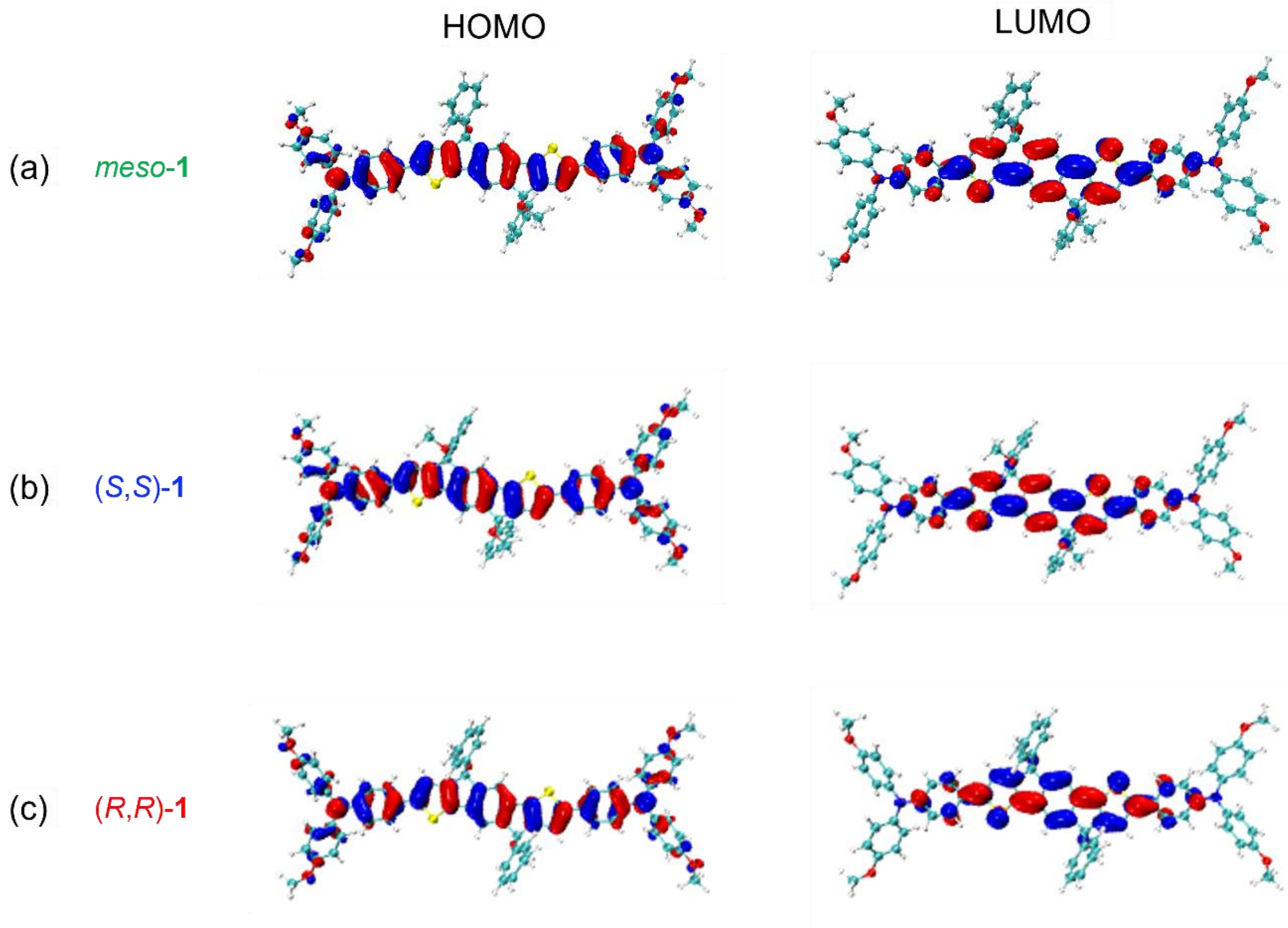

Figure S2. HOMO (left) and LUMO (right) lobes of (a) *meso*-**1**, (b) (*S*,*S*)-**1** and (c) (*R*,*R*)-**1** calculated by DFT at B3LYP/6-31G(d,p) level. To simplify the calculations, tetraethylene glycoloxy was replaced by methoxy.

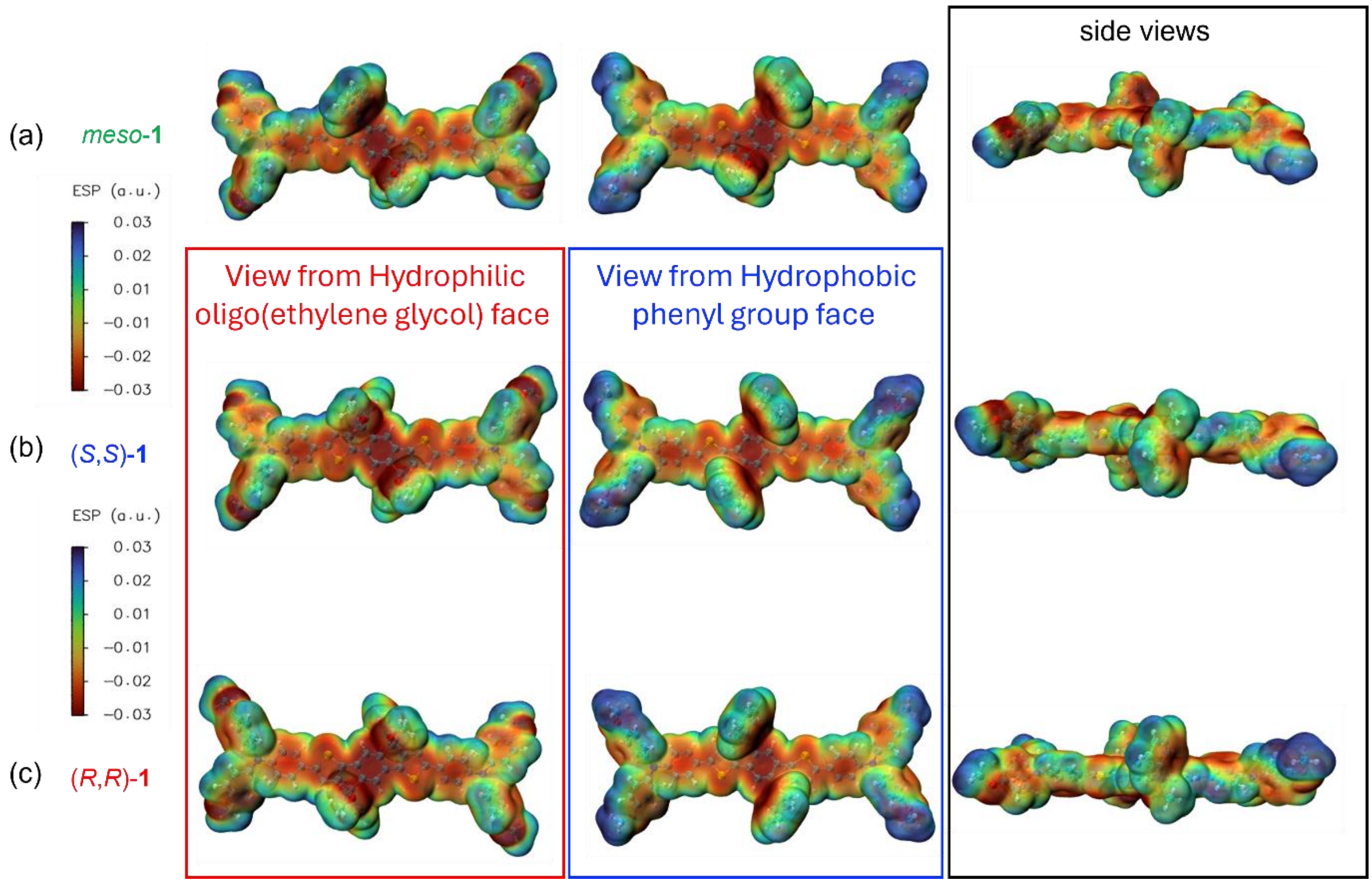

Figure S3. Electrostatic potential (ESP) maps of (a) *meso*-**1**, (b) (*S*,*S*)-**1** and (c) (*R*,*R*)-**1** calculated by DFT at B3LYP/6-31G(d,p) level. To simplify the calculations, tetraethylene glycoloxy was replaced by methoxy. In the cases of (*S*,*S*)-**1** and (*R*,*R*)-**1**, the hydrophilic oligo(ethylene glycol) face exhibits a more electron-rich (more electron-donating) character compared to the phenyl group face, owing to the asymmetric molecular structures.

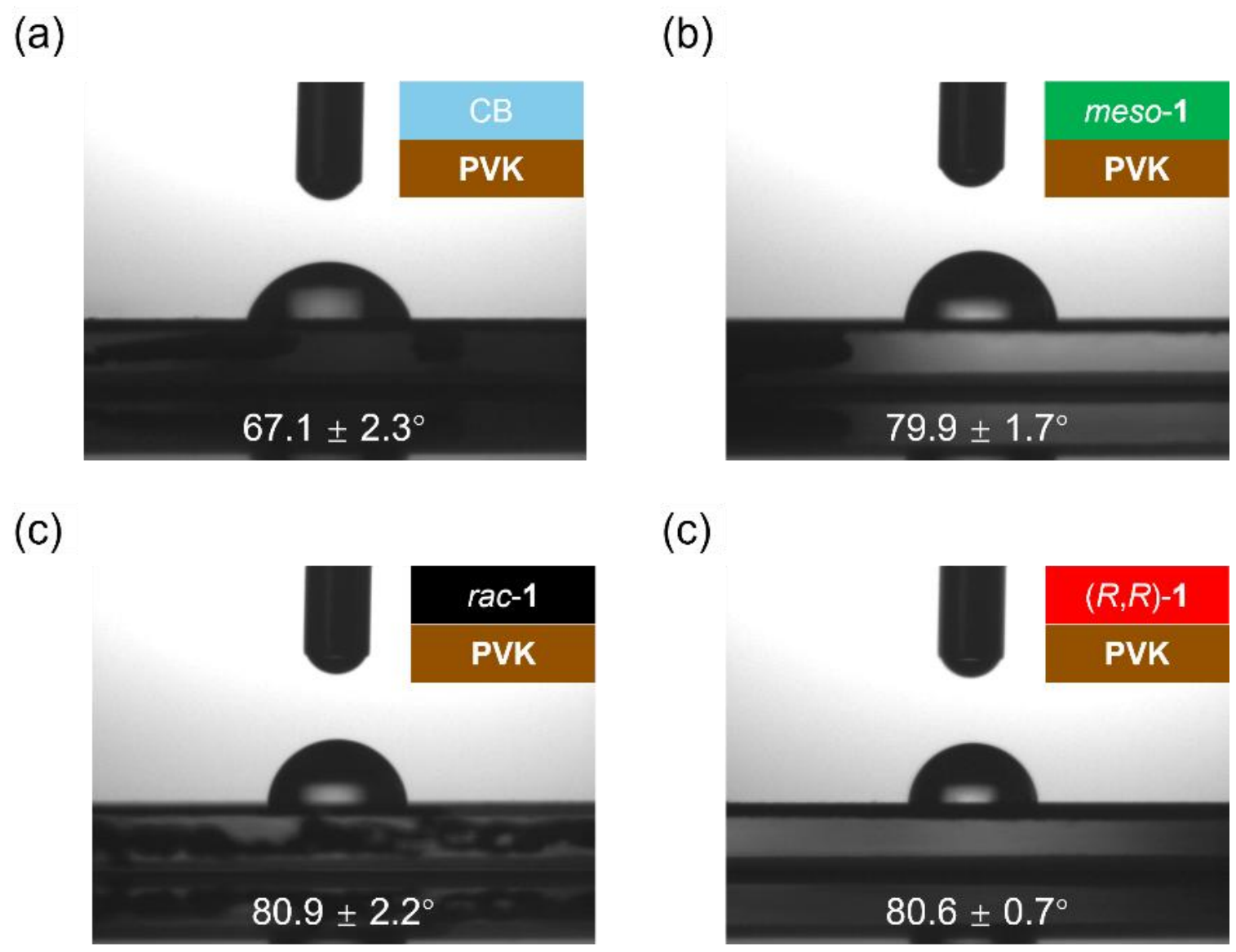

Figure S4. Contact angle (CA) measurements (water droplet) of (a) PVK/CB, (b) PVK/*meso*-**1**, (c) PVK/*rac*-**1**, (d) PVK/(*R*,*R*)-**1**, respectively.

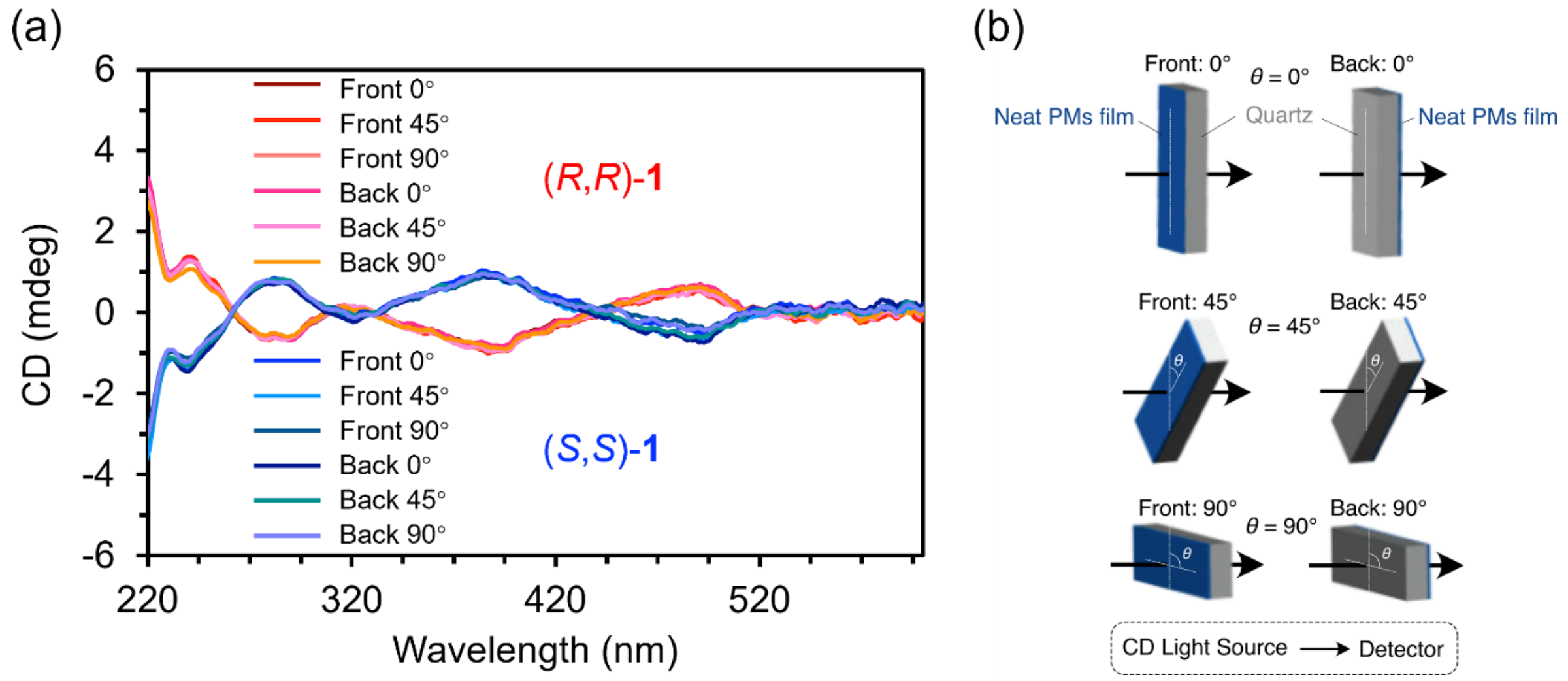

Figure S5. (a) Incident angle and side dependency of CD spectra of (*S*,*S*)-**1** and (*R*,*R*)-**1** thin films on quartz and (b) their schematic illustrations.

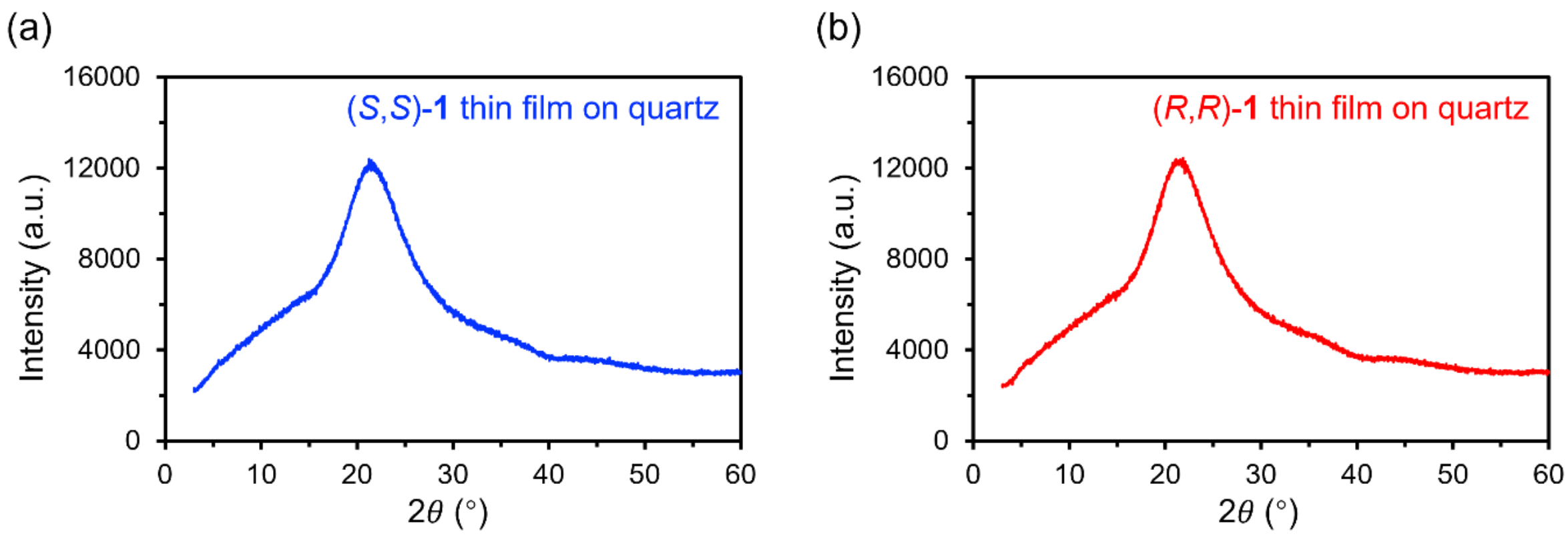

Figure S6. XRD patterns of (a) (*S*,*S*)-**1** and (b) (*R*,*R*)-**1** thin films on quartz.

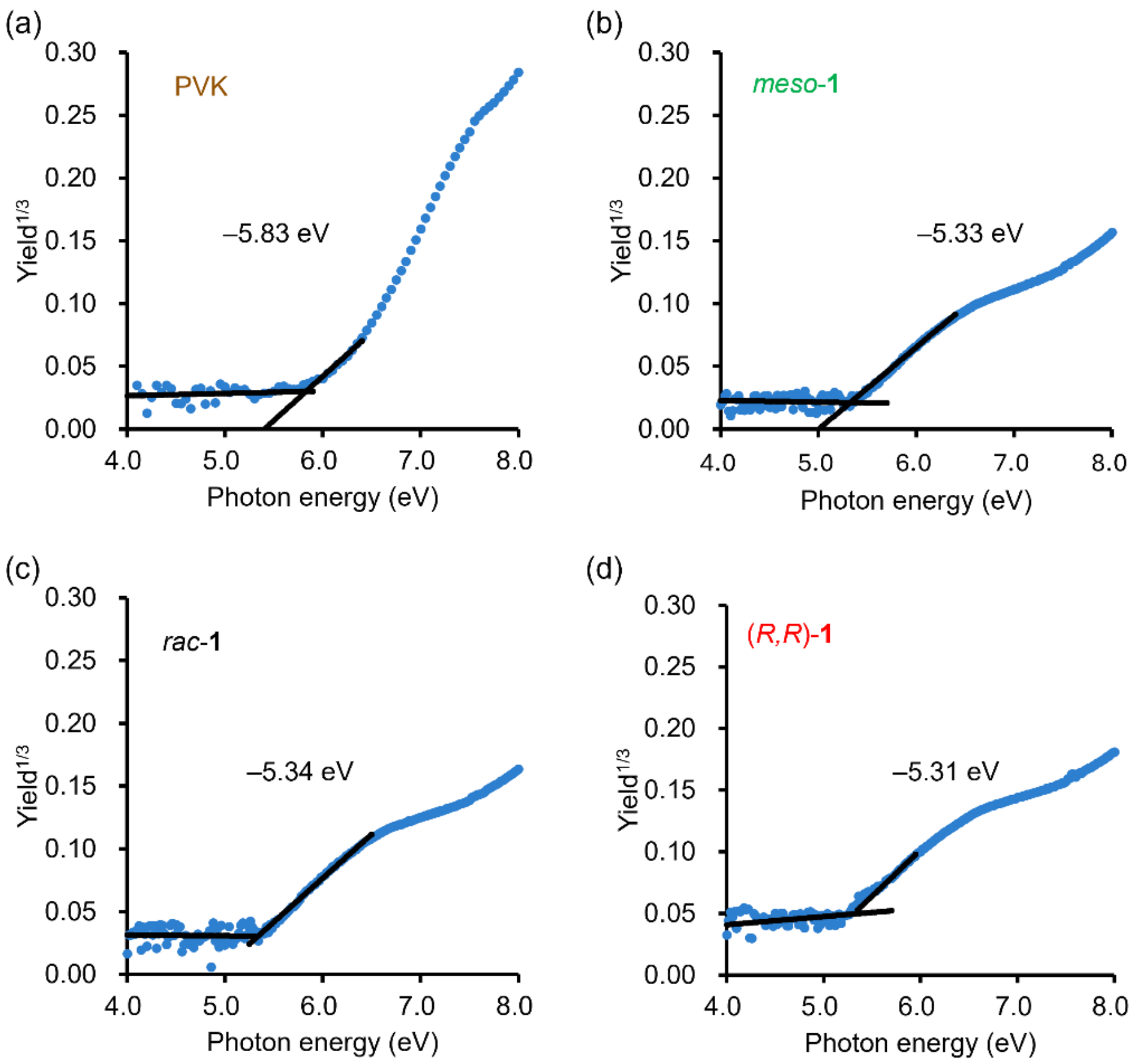

Figure S7. PYS profiles of (a) PVK, (b) *meso*-**1**, (c) *rac*-**1** and (d) (*R*,*R*)-**1** in vacuum. The onset energy was calculated by least square fitting (the solid lines).

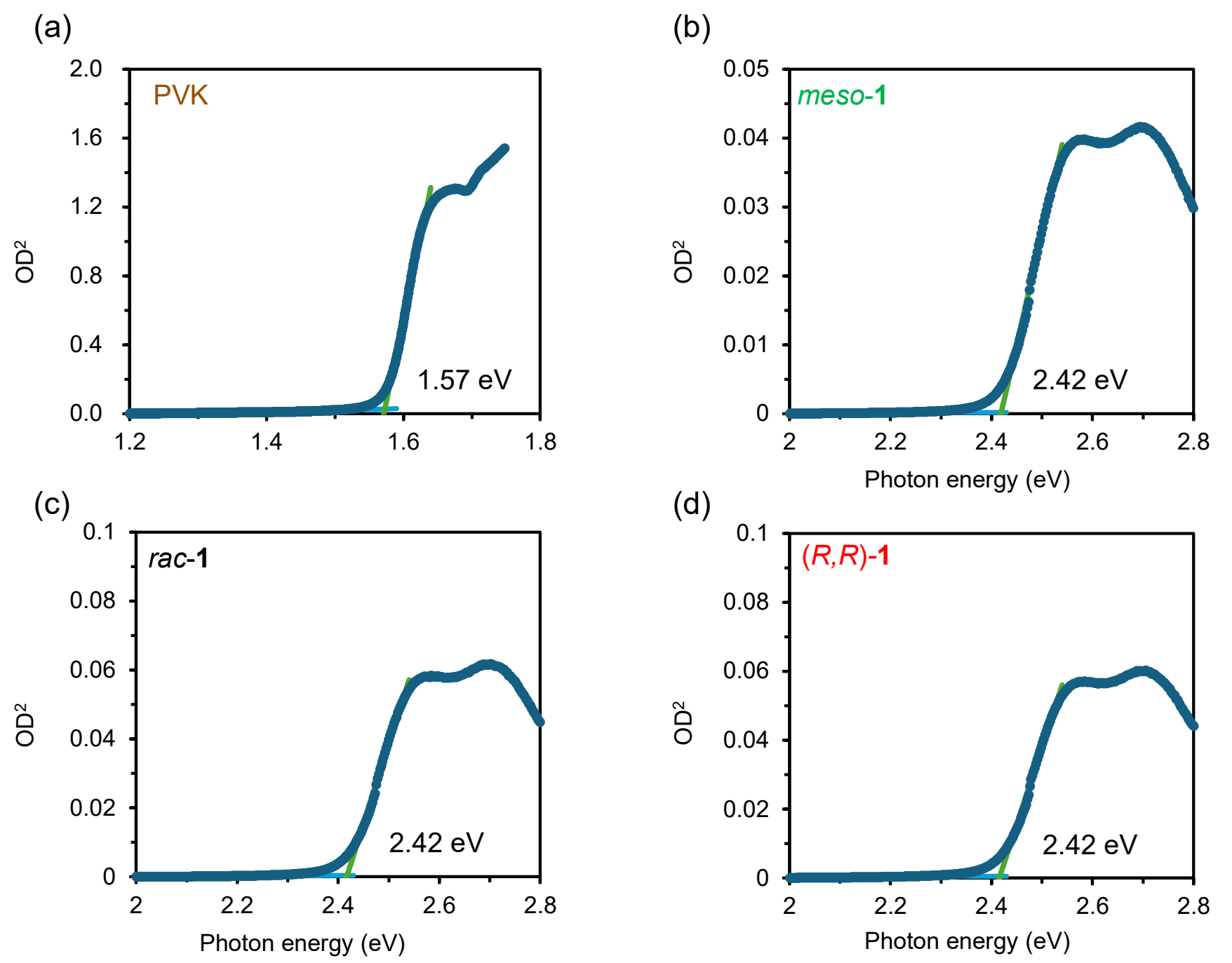

Figure S8. Bandgap energy calculations of (a) PVK, (b) *meso*-**1**, (c) *rac*-**1** and (d) (*R*,*R*)-**1** films on quartz.

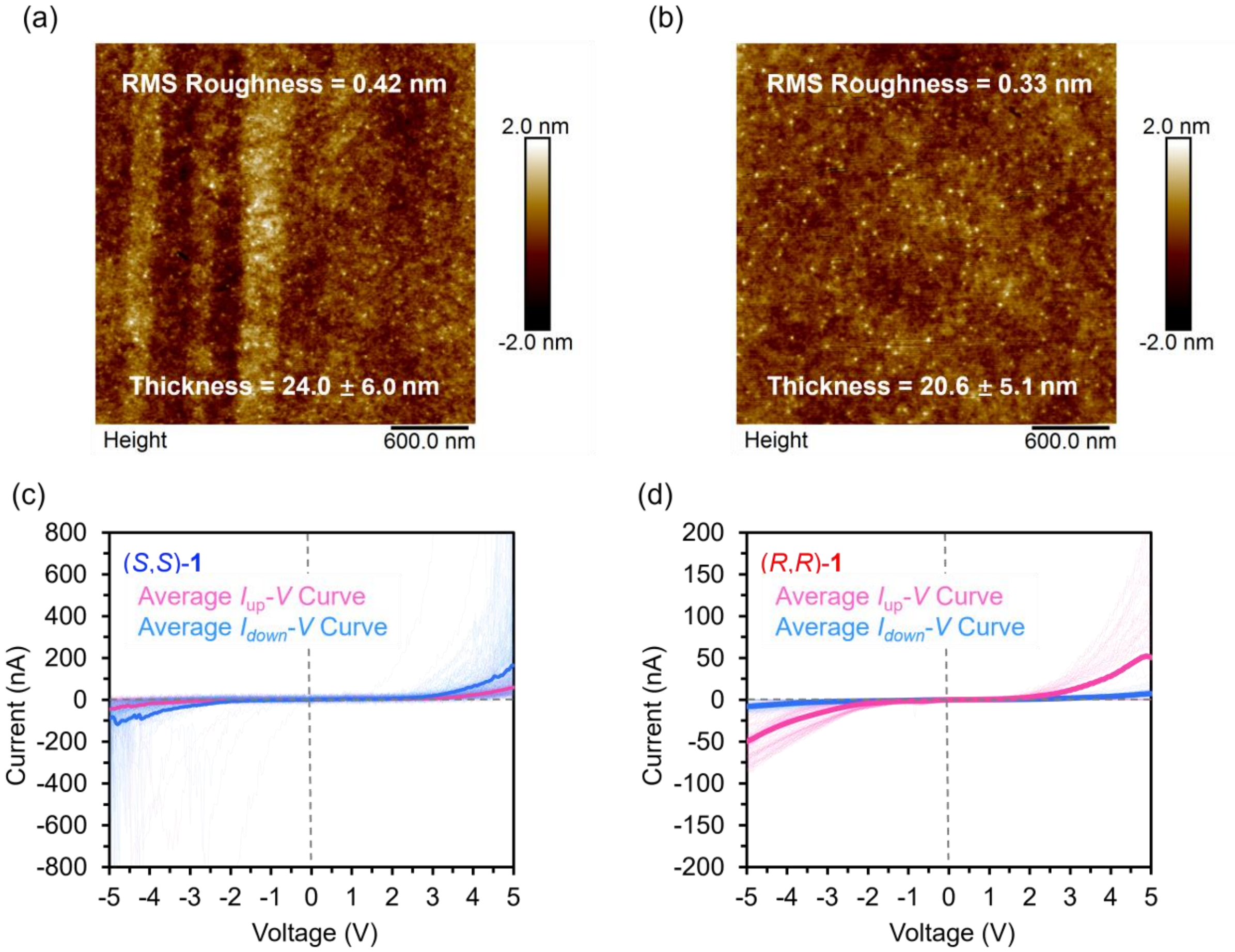

Figure S9. AFM height images of spin-coat thin films of (a) (*S*,*S*)-**1** and (b) (*R*,*R*)-**1** on HOPG. The film thicknesses were 24.0 ± 6.0 and 20.6 ± 5.1 nm, respectively. The root-mean-square roughness values are superimposed. Full range views of up (pink) or down (sky blue) spin-polarized current-voltage curves of the thin films of (c) (*S*,*S*)-**1** and (d) (*R*,*R*)-**1** on HOPG. Unaveraged curves are shown as thin pink and blue lines.

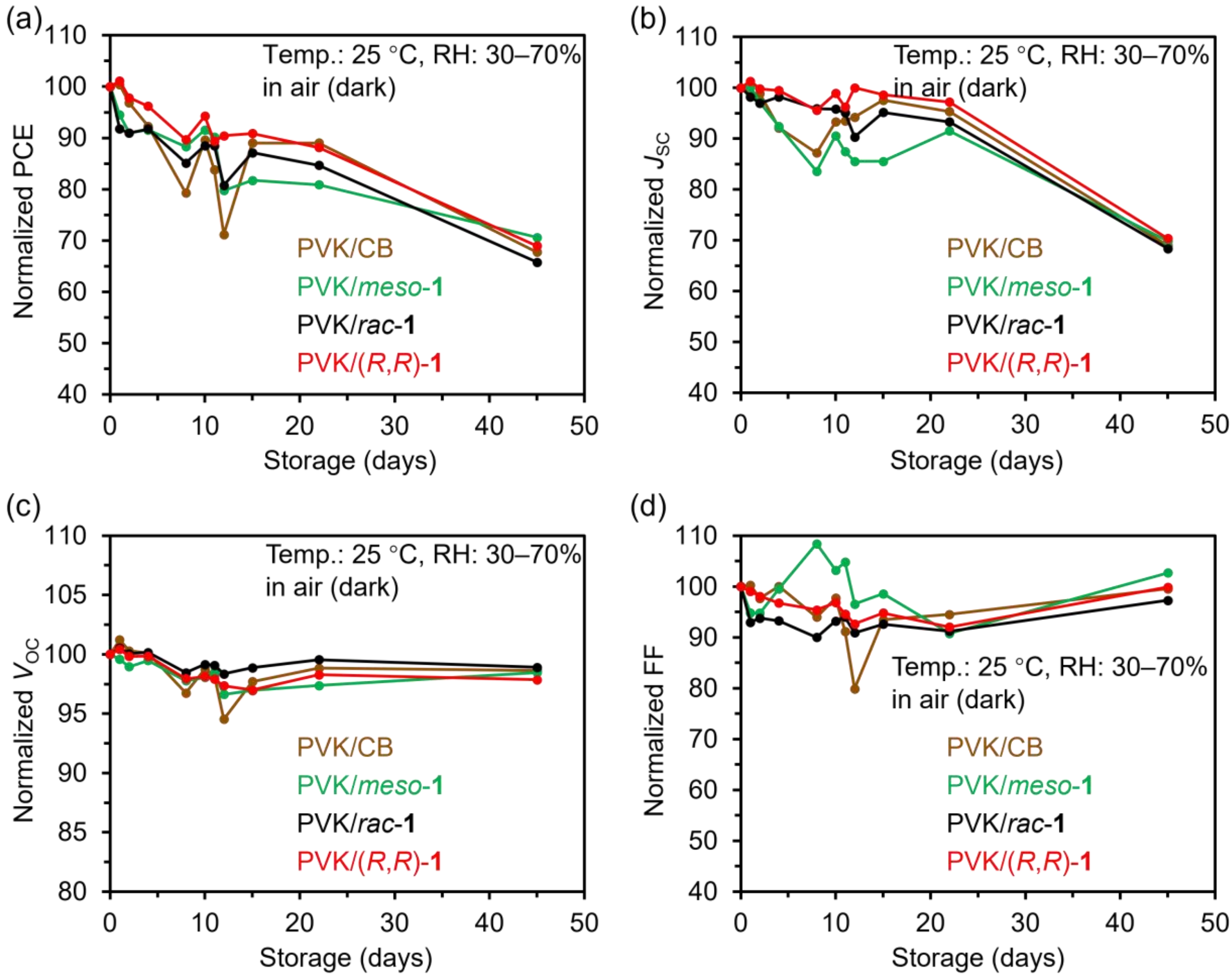

Figure S10. Normalized (a) PCE, (b) J_{SC}, (c) V_{OC}, and (d) FF during storage. Temperature: 25 °C and RH: 30–70% in the air and darkness.

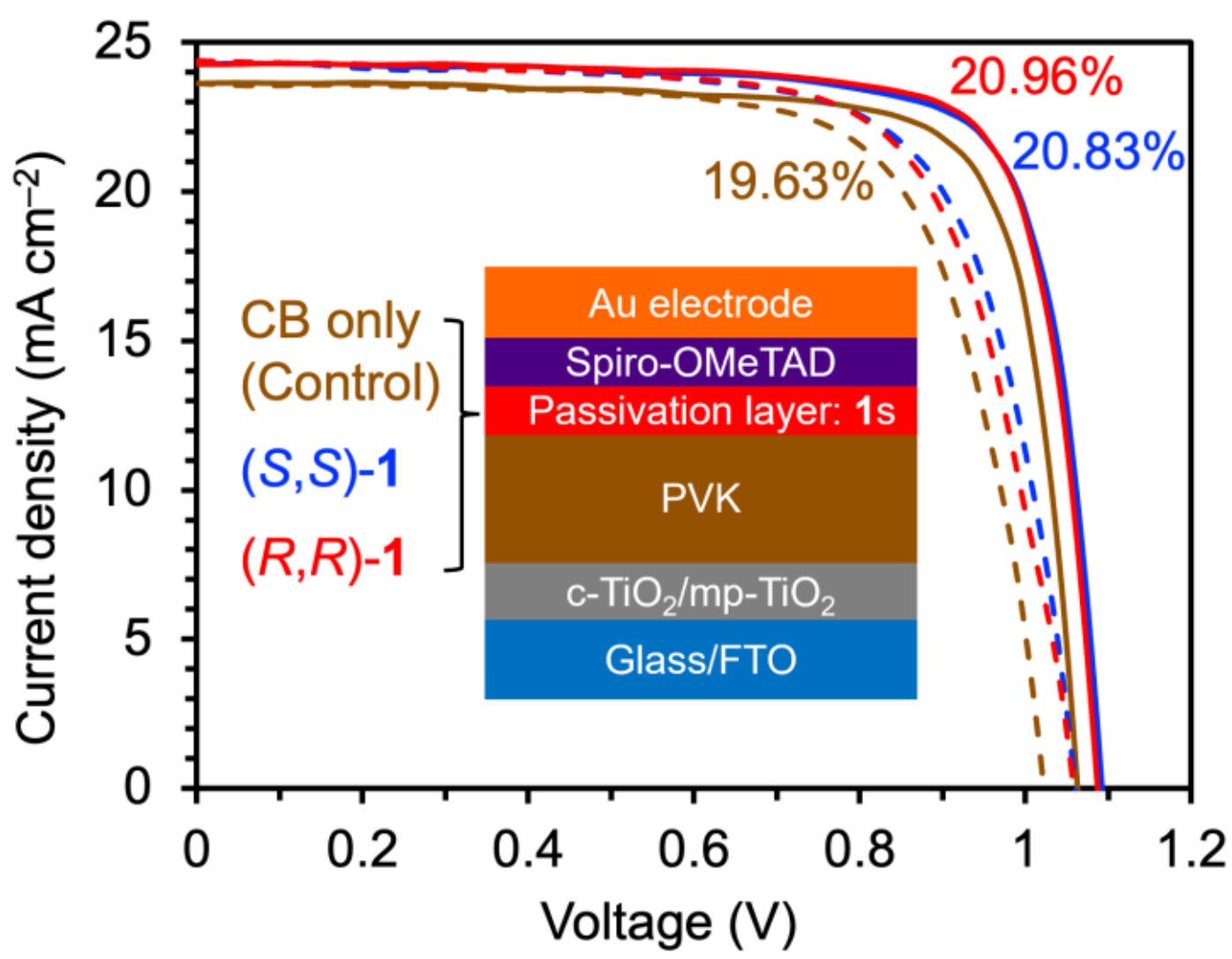

Figure S11. Best-performing *J*-*V* curves of control (brown), (*S*,*S*)-**1**-treated (blue) and (*R*,*R*)-**1**-treated (red) PSCs (FTO/cTiO$_2$/mpTiO$_2$/PVK ($FA_{0.85}MA_{0.1}Cs_{0.05}PbI_3$) w/wo **1** treatment/doped spiro-OMeTAD/Au). The dotted and solid lines are forward and reverse scans, respectively.

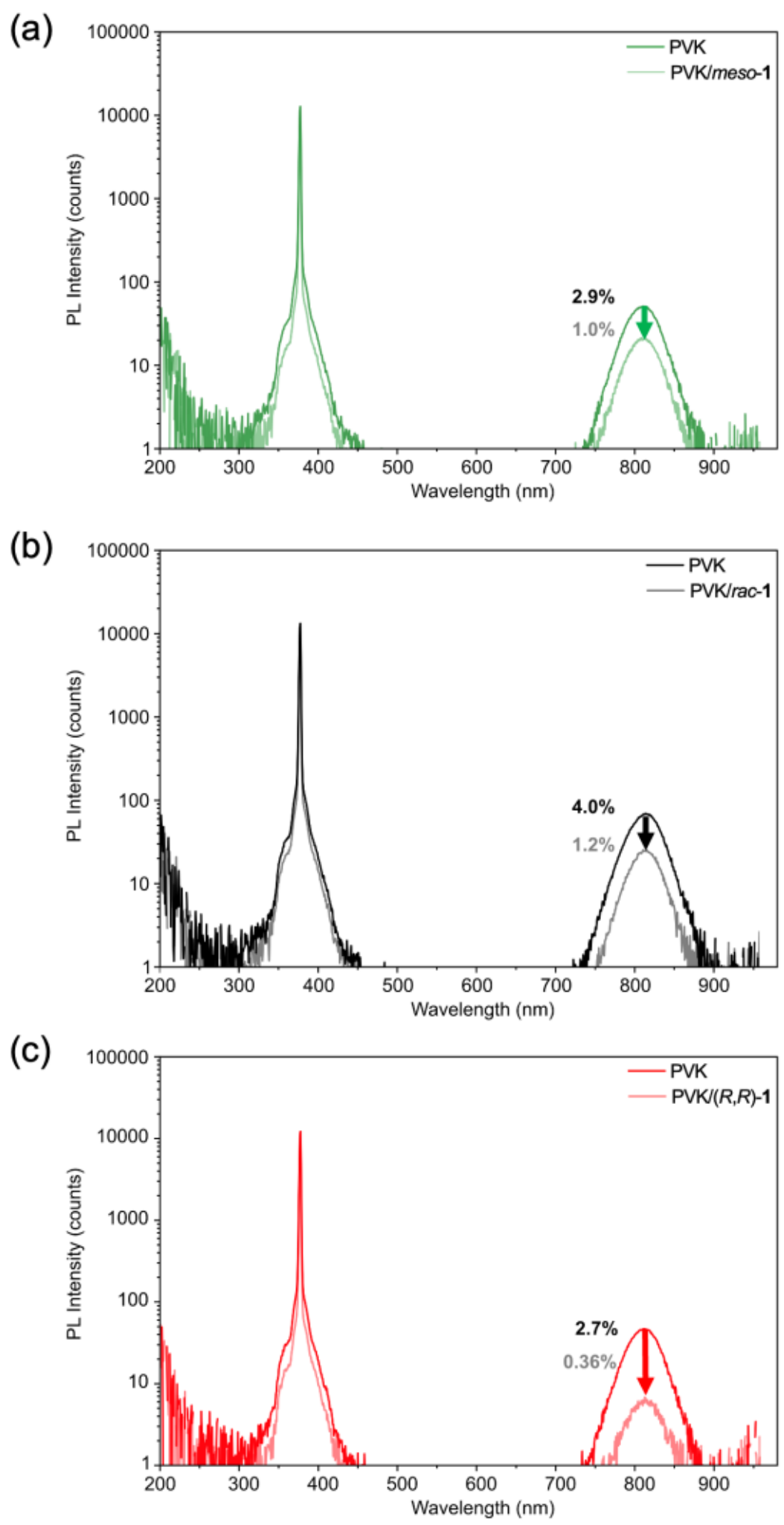

Figure S12. Photoluminescence quantum yield (PLQY) of the perovskite film before/after (a) *meso*-**1**, (b) *rac*-**1** or (c) (*R*,*R*)-**1** treatments.

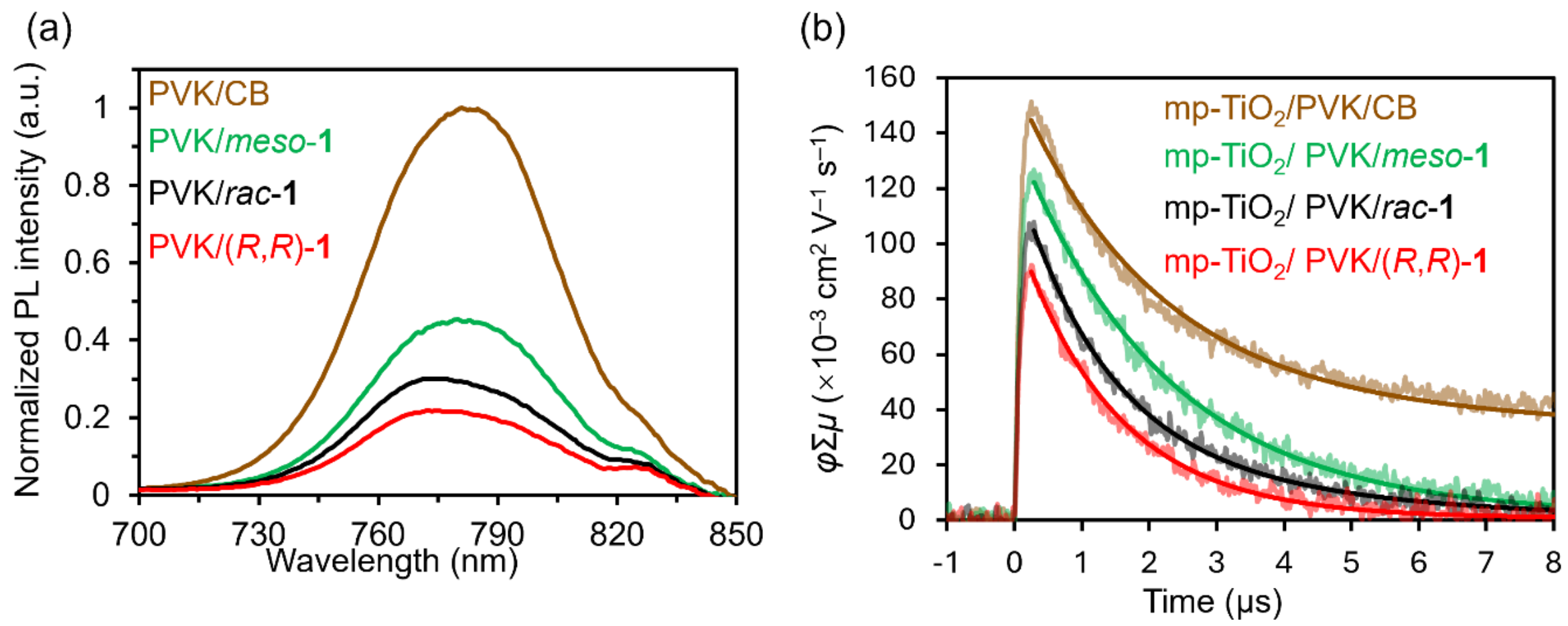

Figure S13. (a) PL spectra (λ_{ex} = 500 nm). (b) TRMC transients (λ_{ex} = 500 nm). ETL is mpTiO$_2$. The solid thick lines are double-exponential fitting curves. The PVK composition is $MA_{0.13}FA_{0.87}PbI_{2.61}Br_{0.39}$.

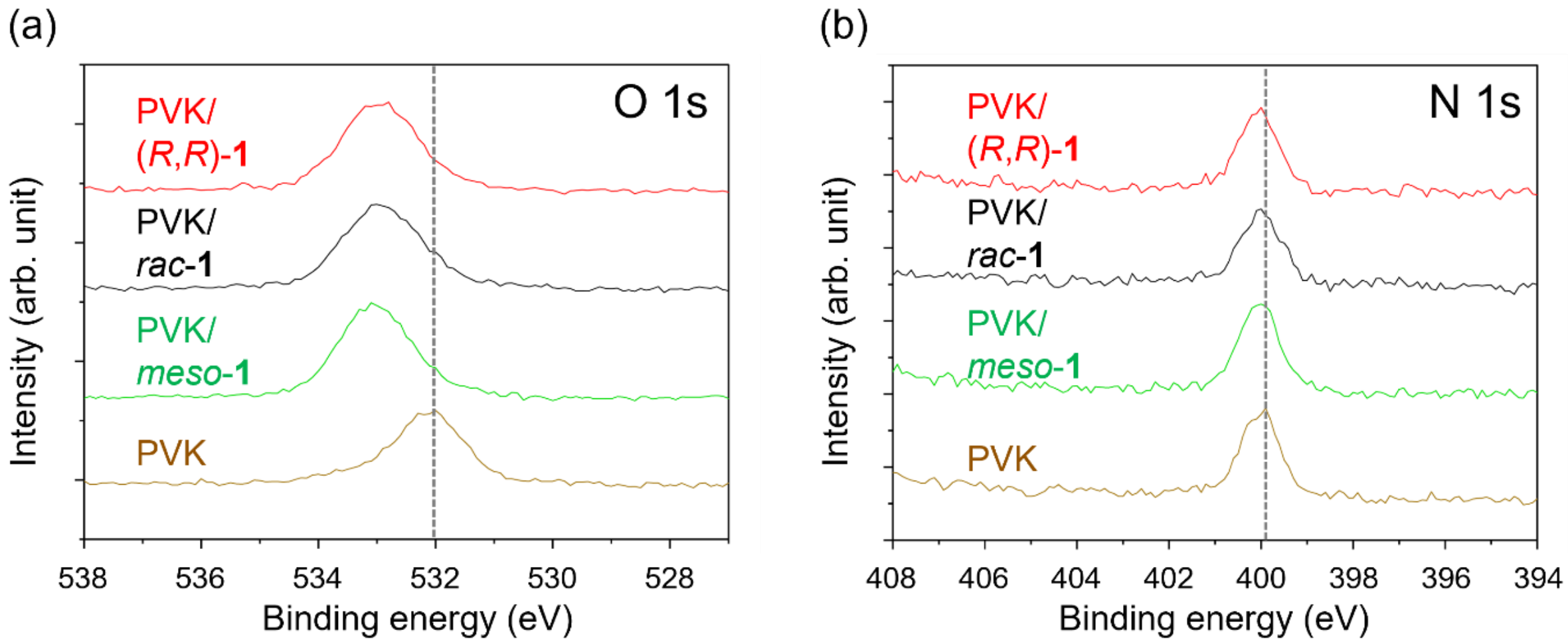

Figure S14. X-ray photoelectron spectra at (a) O 1s and (b) N 1s with/without *meso*-**1**, *rac*-**1** or (*R*,*R*)-**1** passivation.

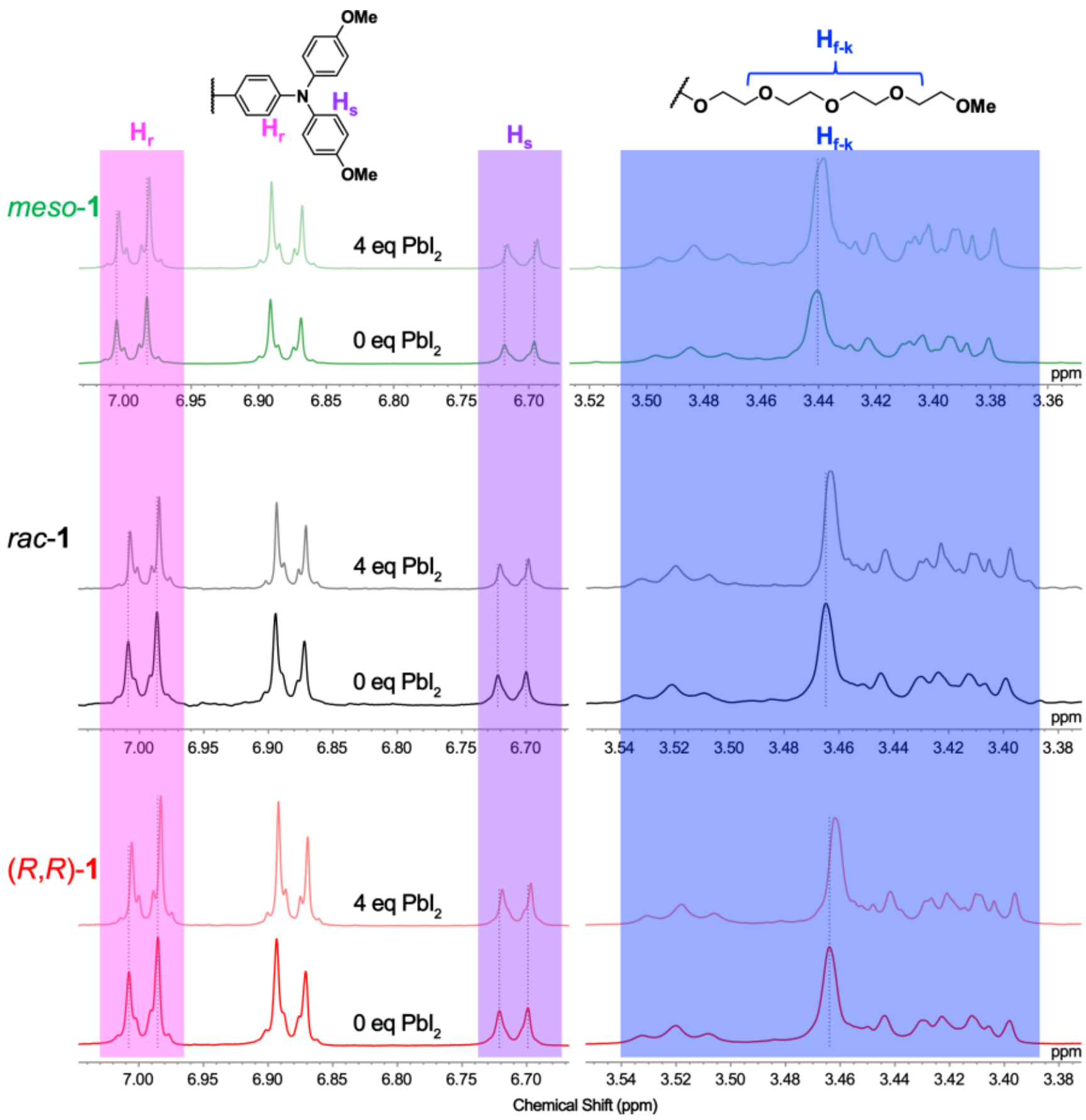

Figure S15. ^{1}H NMR spectra (400 MHz) of *meso*-**1**, *rac*-**1**, and (*R*,*R*)-**1** with/without PbI_2 in DMSO-d_6 at 25 °C.

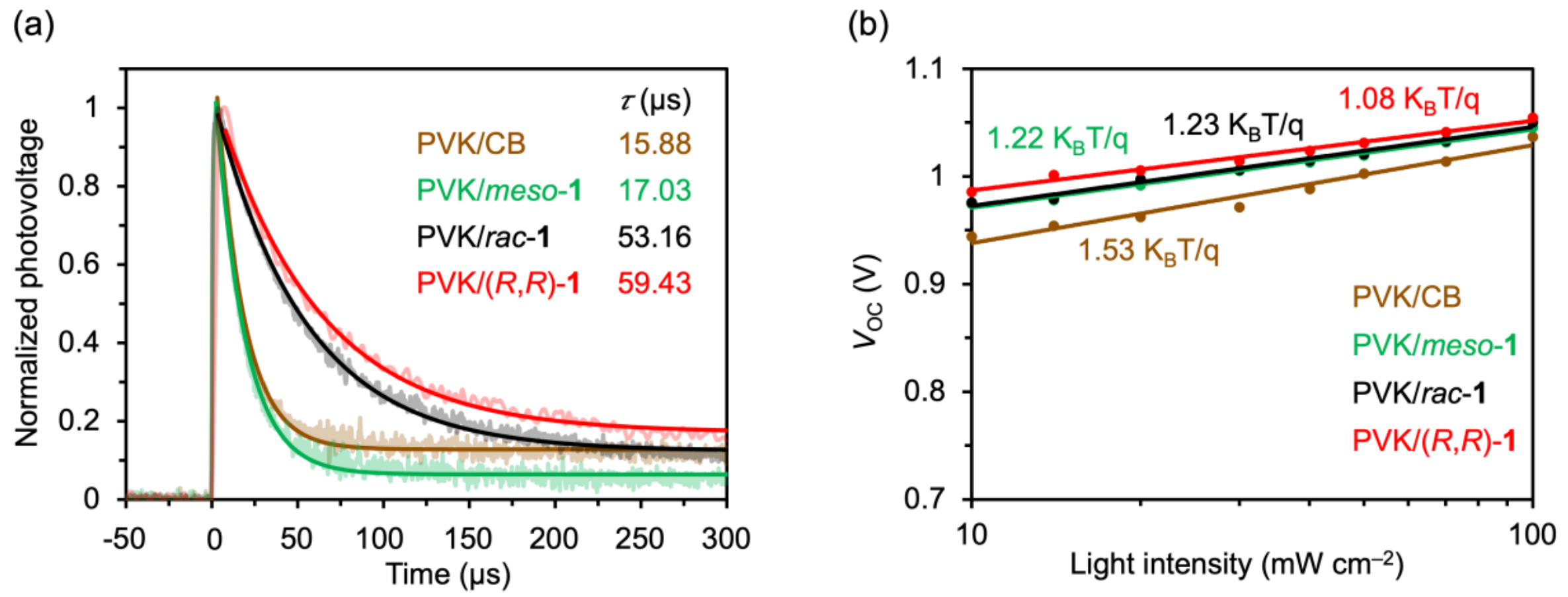

Figure S16. (a) Transient photovoltage (TPV) decays and (b) light-intensity-dependent V_{OC} measurements of PSCs with chlorobenzene, *meso*-**1**, *rac*-**1**, and (*R*,*R*)-**1** treatments.

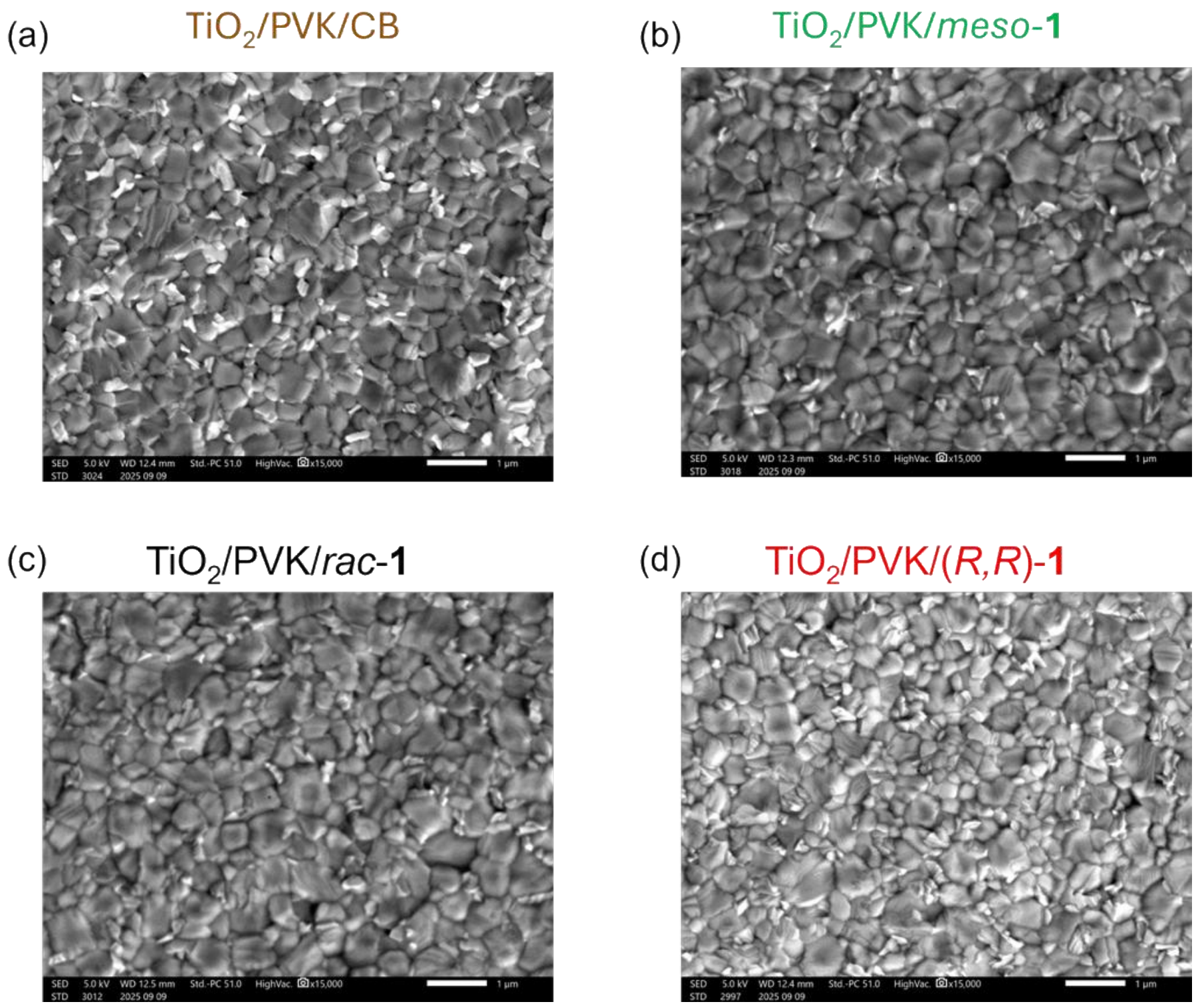

Figure S17. SEM images of (a) CB-treated PVK, (b) *meso*-**1**-treated PVK, (c) *rac*-**1**-treated PVK and (*R*,*R*)-**1**-treated PVK. The scale bar is 1 μm.

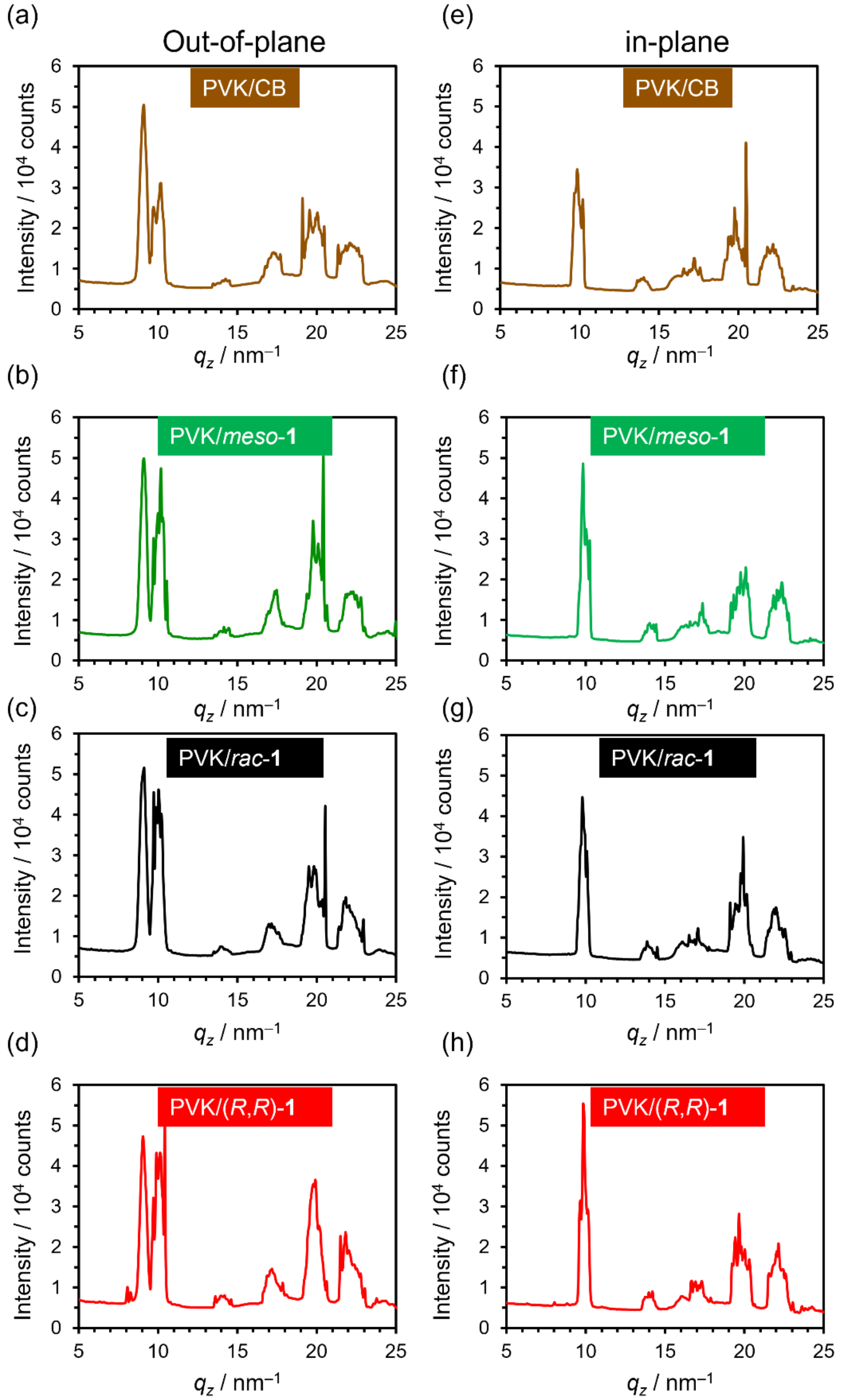

Figure S18. 2D-GIXRD profiles of (a) CB-treated PVK, (b) *meso*-**1**-treated PVK, (c) *rac*-**1**-treated PVK and (d) (*R*,*R*)-**1**-treated PVK in out-of-plane (OOP) direction. 2D-GIXRD profiles of (e) CB-treated PVK, (f) *meso*-**1**-treated PVK, (g) *rac*-**1**-treated PVK and (h) (*R*,*R*)-**1** treated PVK in in-plane (IP) direction.

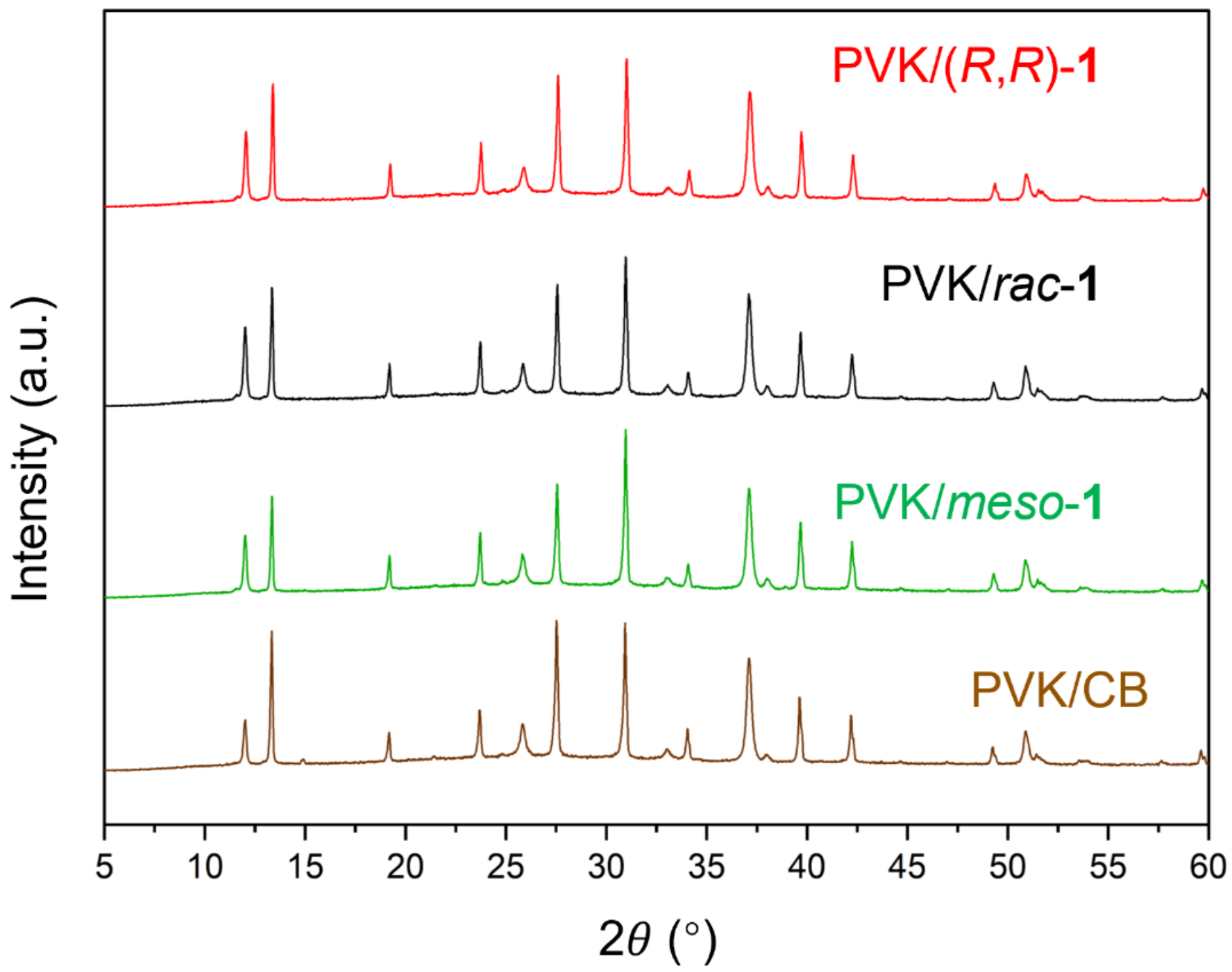

Figure S19. Power XRD profiles of PVK with/without *meso*-**1**, *rac*-**1** or (*R*,*R*)-**1** passivation.

8. Analytical Data

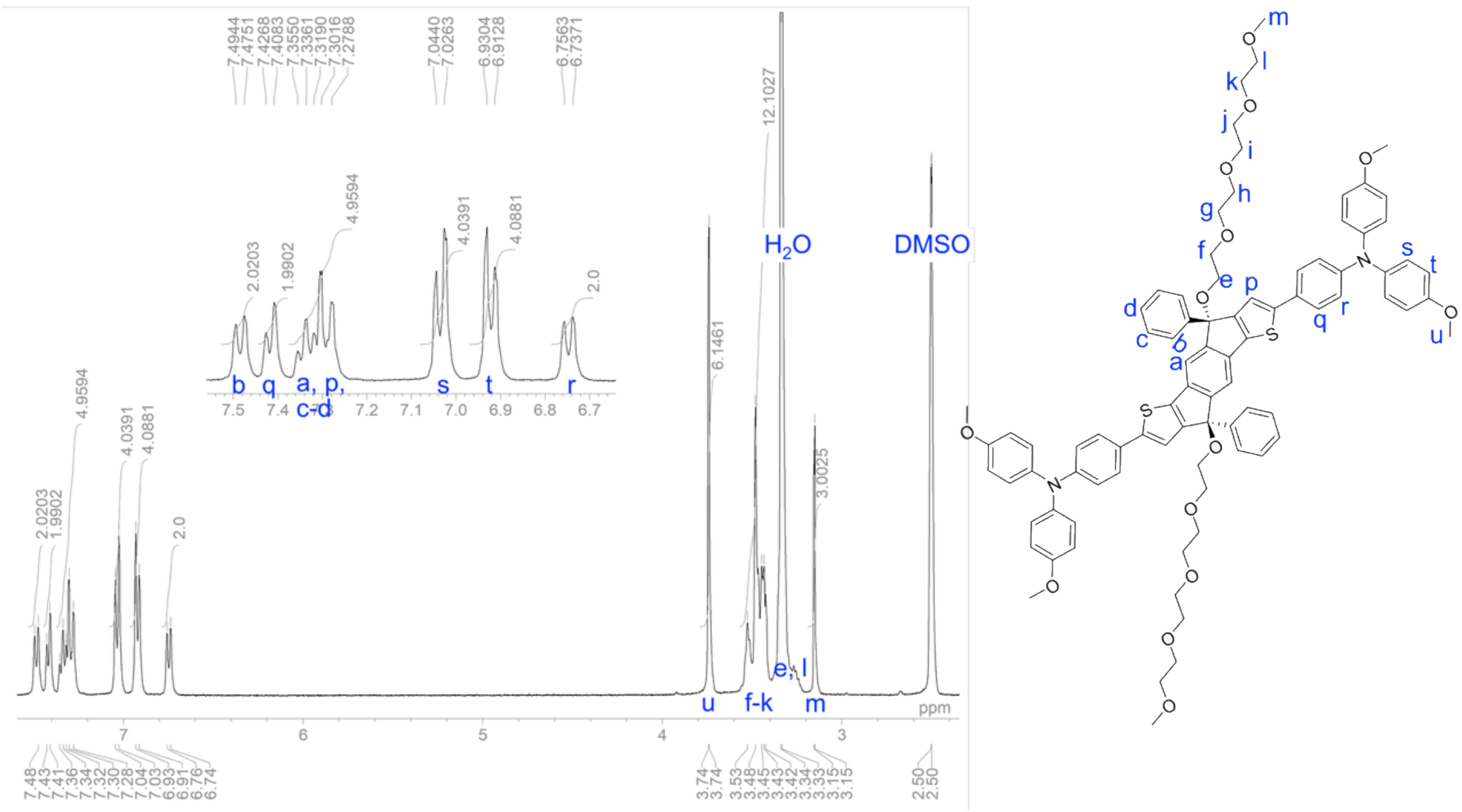

Figure S20. ^{1}H NMR spectrum (400 MHz) of *meso*-**1** in DMSO-d$_6$ at 25 °C.

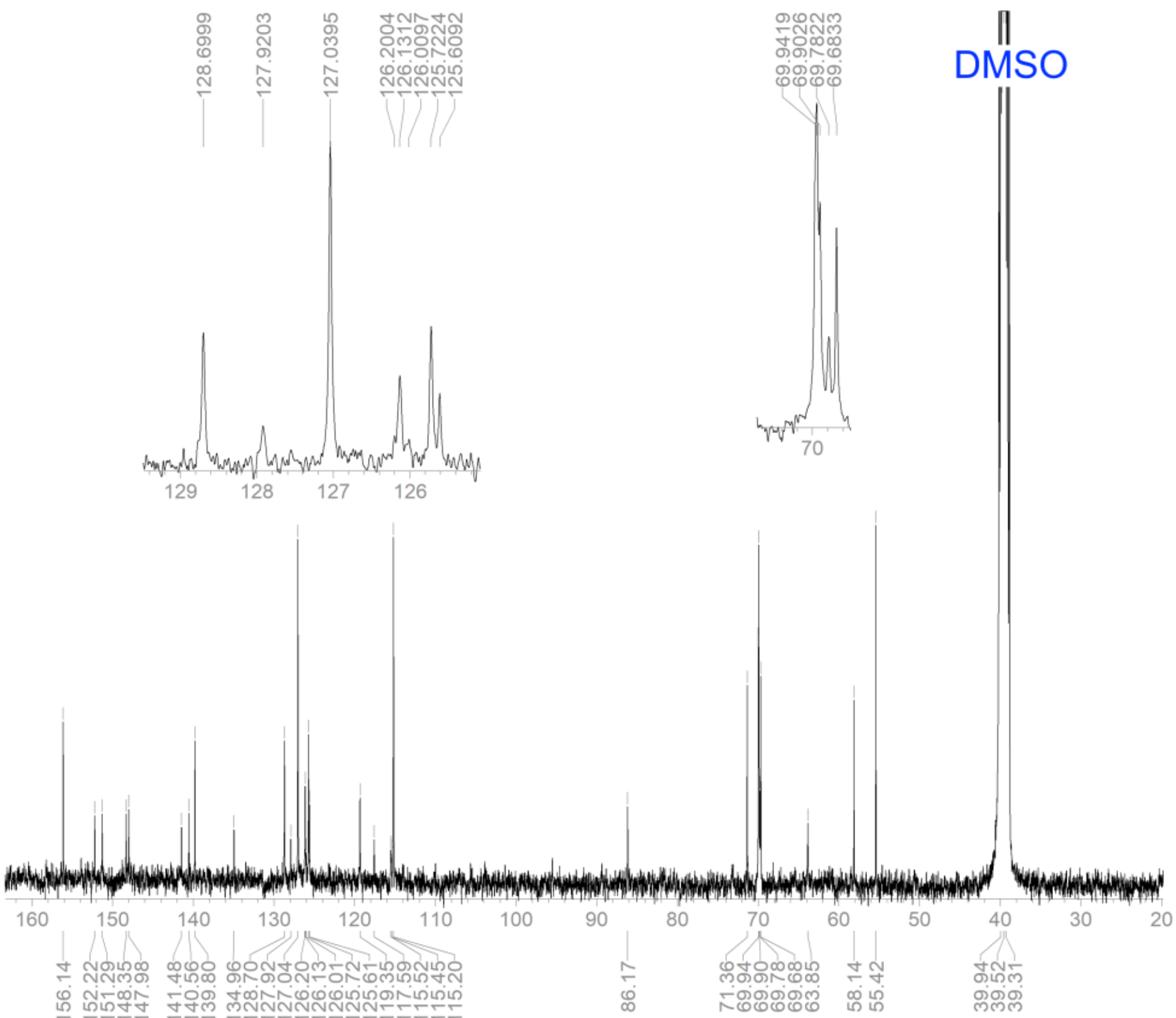

Figure S21. ^{13}C NMR spectrum (100 MHz) of *meso*-**1** in DMSO-d$_6$ at 25 °C.

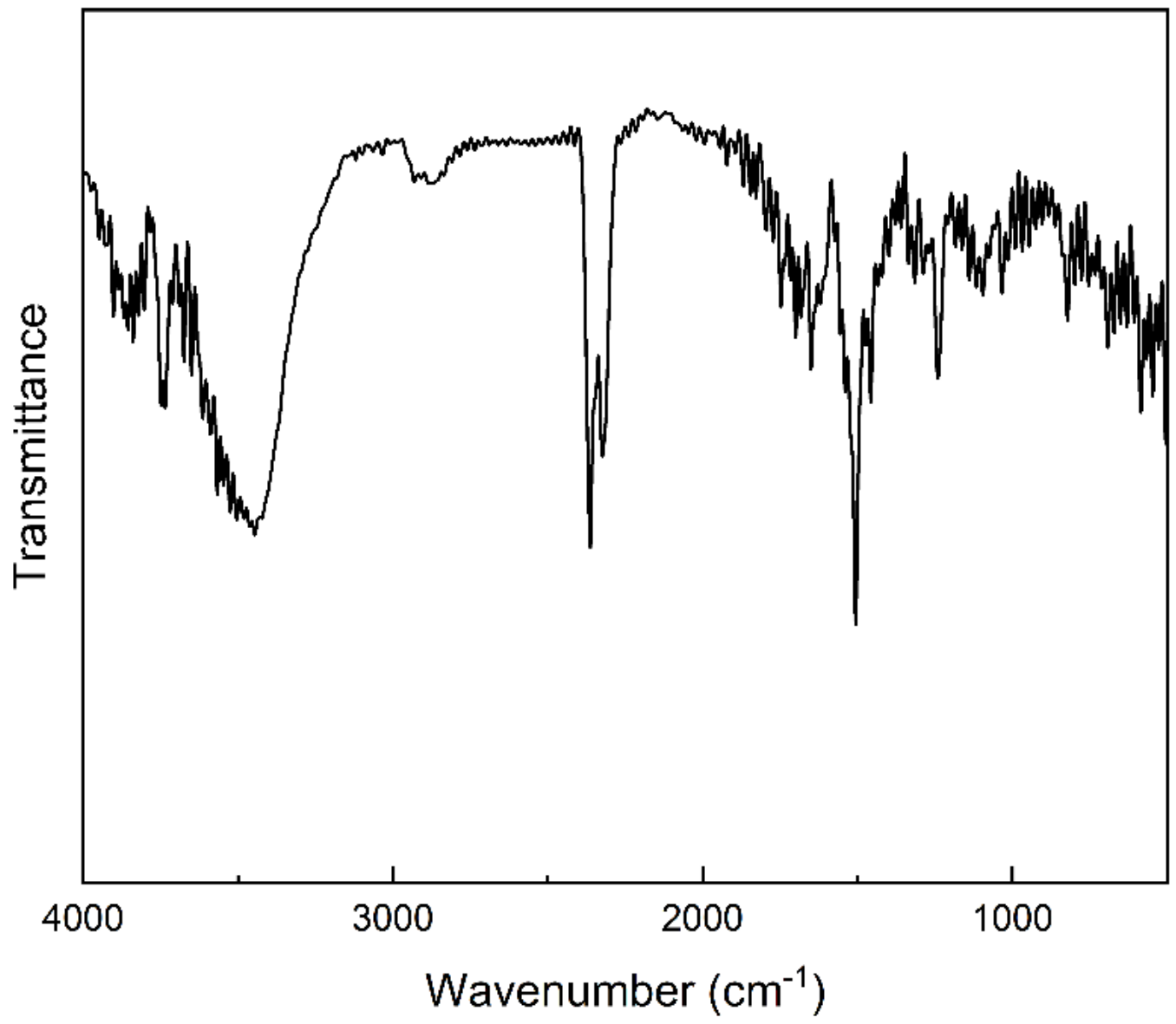

Figure S22. FT-IR spectrum of *meso*-**1** at 25 °C (KBr).

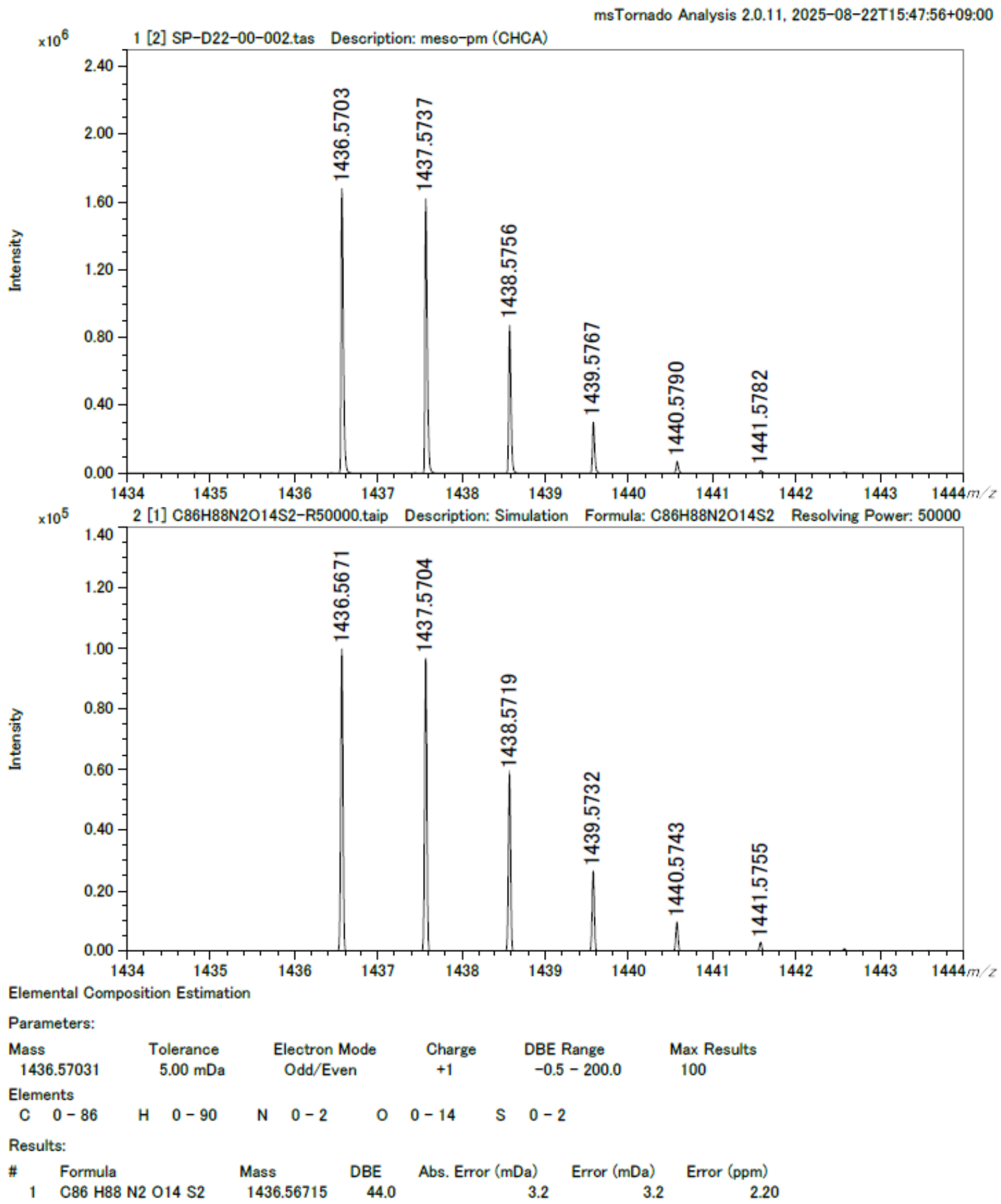

Figure S23. Observed (upper) and simulated (lower) high-resolution MALDI-TOF mass spectra of *meso*-**1**.

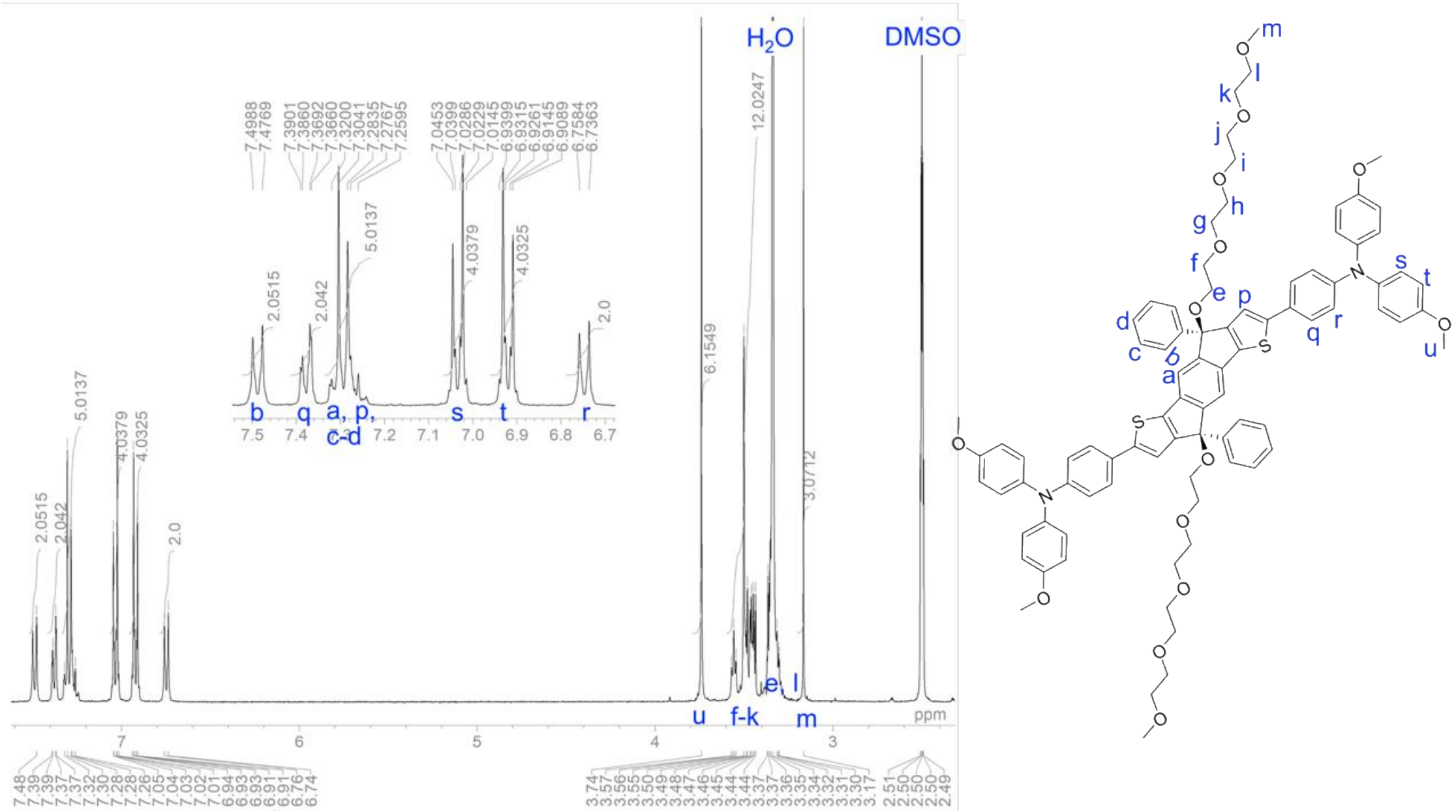

Figure S24. ^{1}H NMR spectrum (400 MHz) of *rac*-**1** in DMSO-d$_6$ at 25 °C.

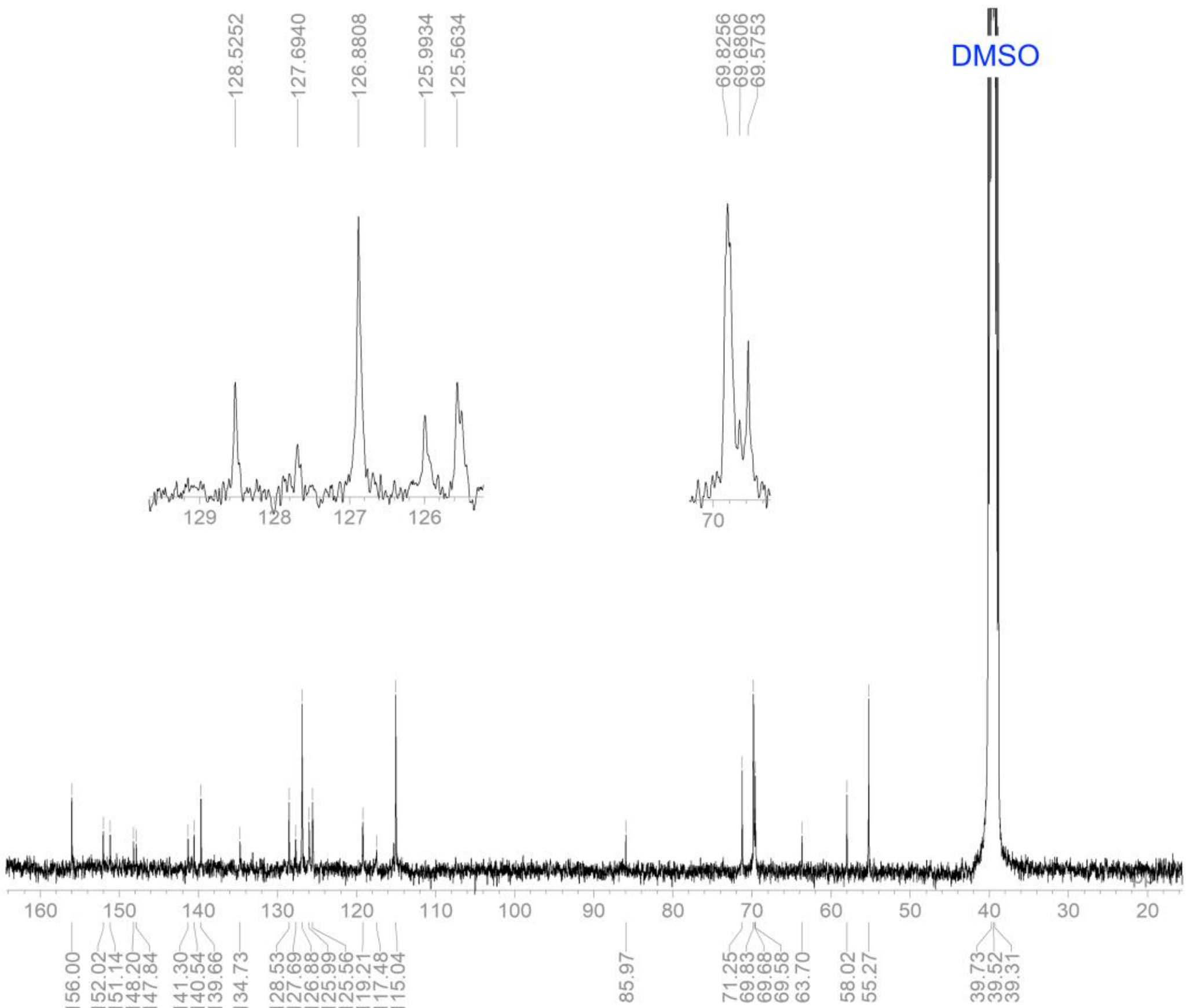

Figure S25. ^{13}C NMR spectrum (100 MHz) of *rac*-**1** in DMSO-d$_6$ at 25 °C.

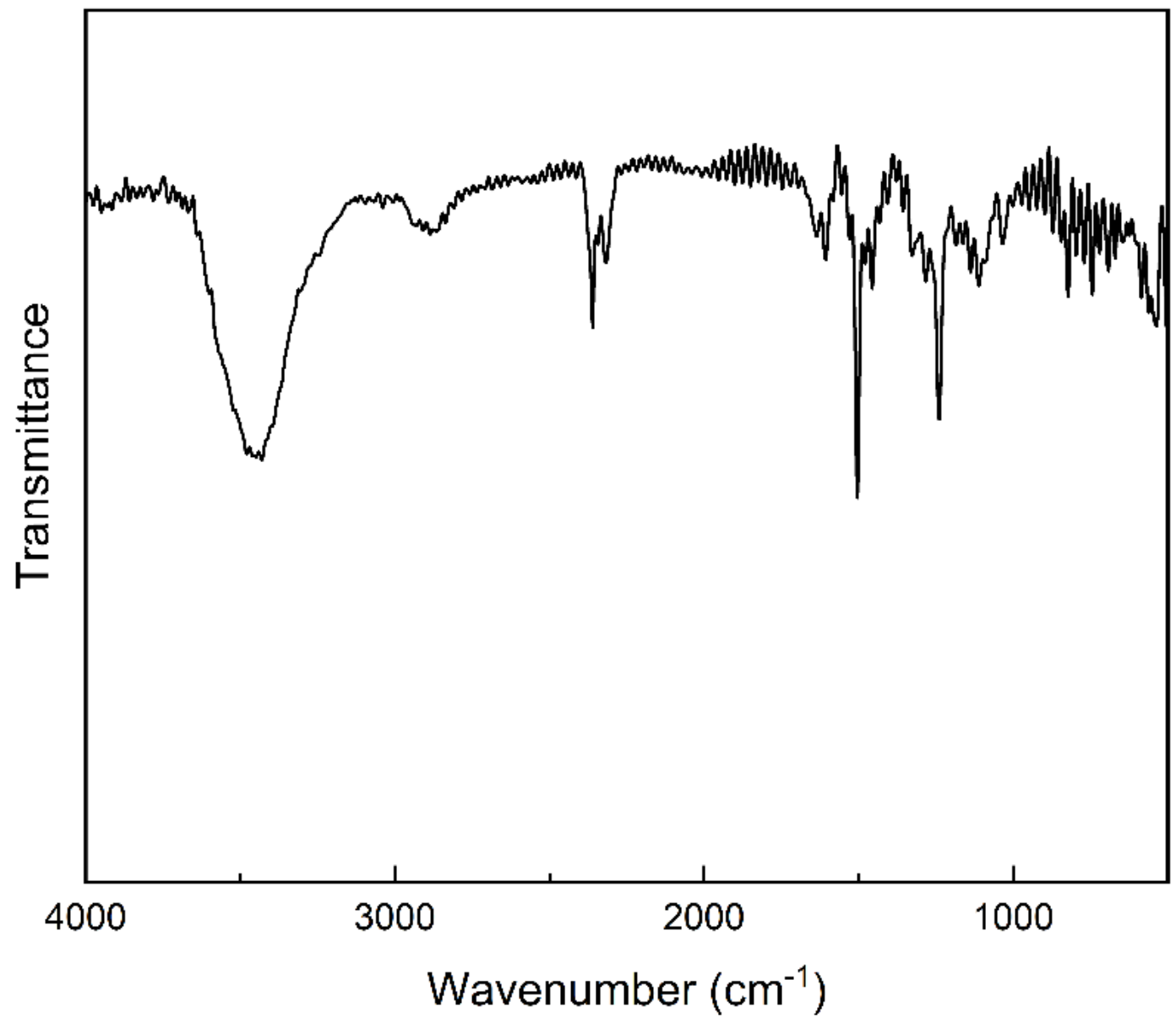

Figure S26. FT-IR spectrum of *rac*-**1** at 25 °C (KBr).

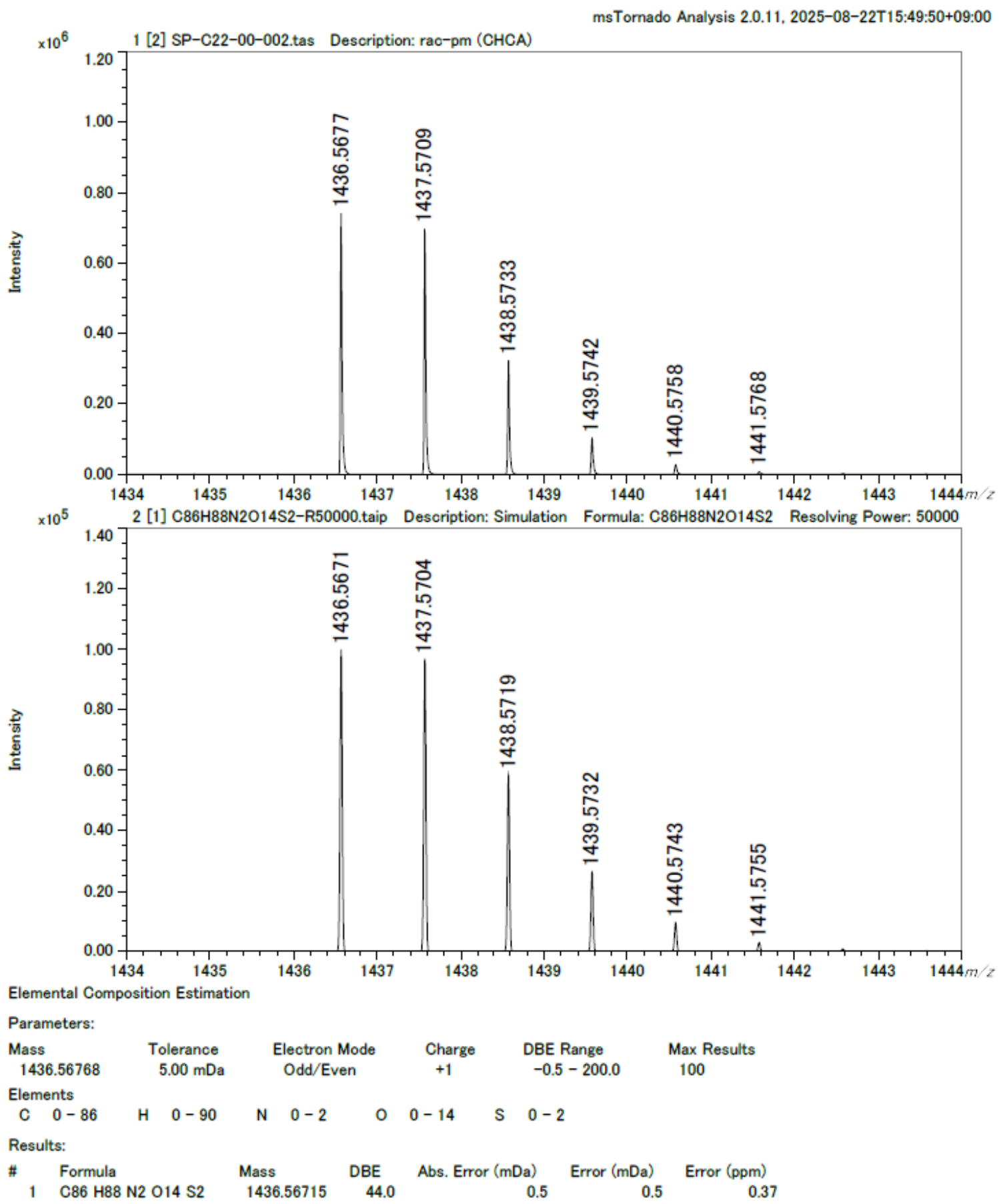

Figure S27. Observed (upper) and simulated (lower) high-resolution MALDI-TOF mass spectra of *rac*-**1**.

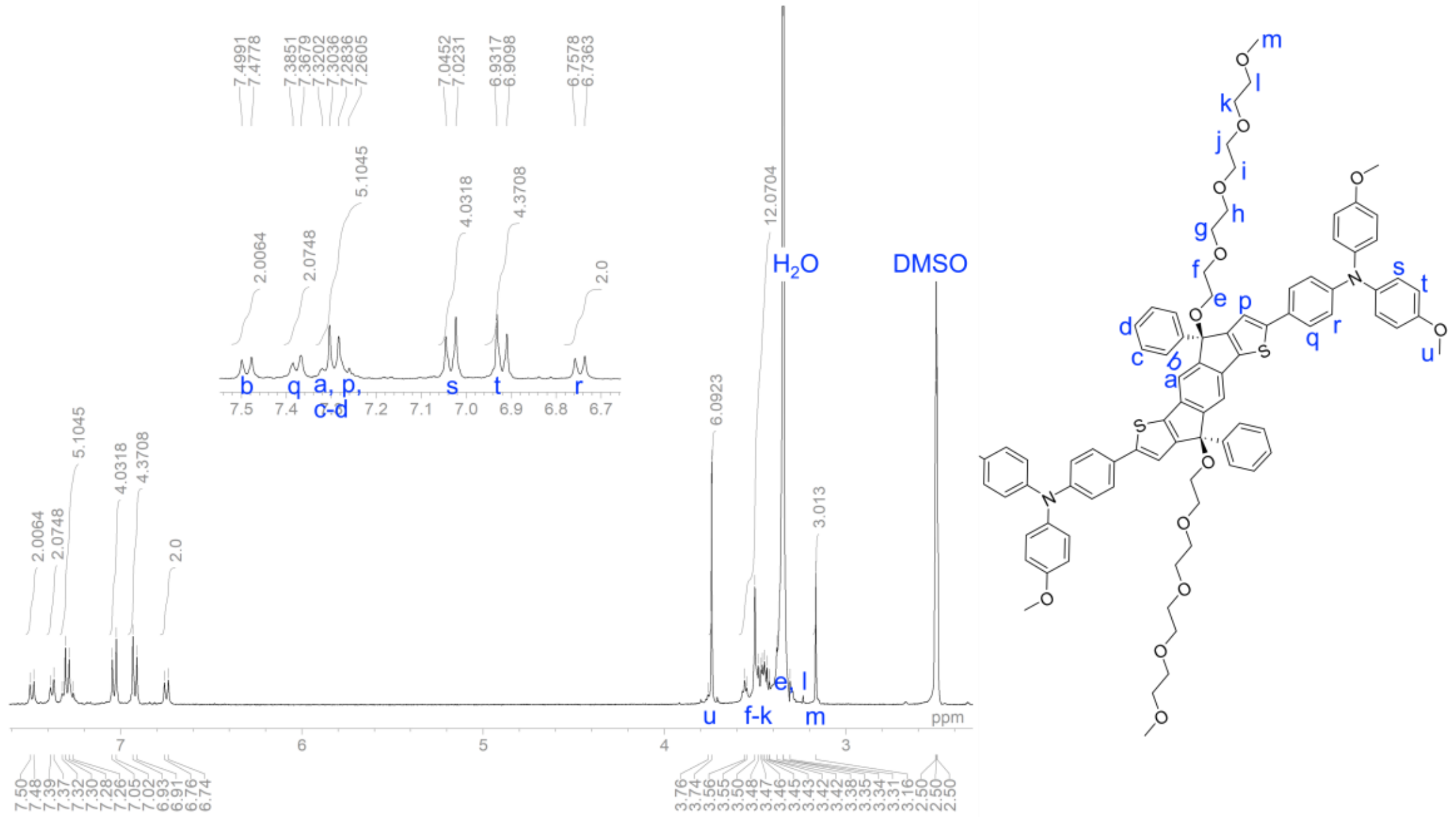

Figure S28. ^{1}H NMR spectrum (400 MHz) of (*S*,*S*)-**1** in DMSO-d$_6$ at 25 °C.

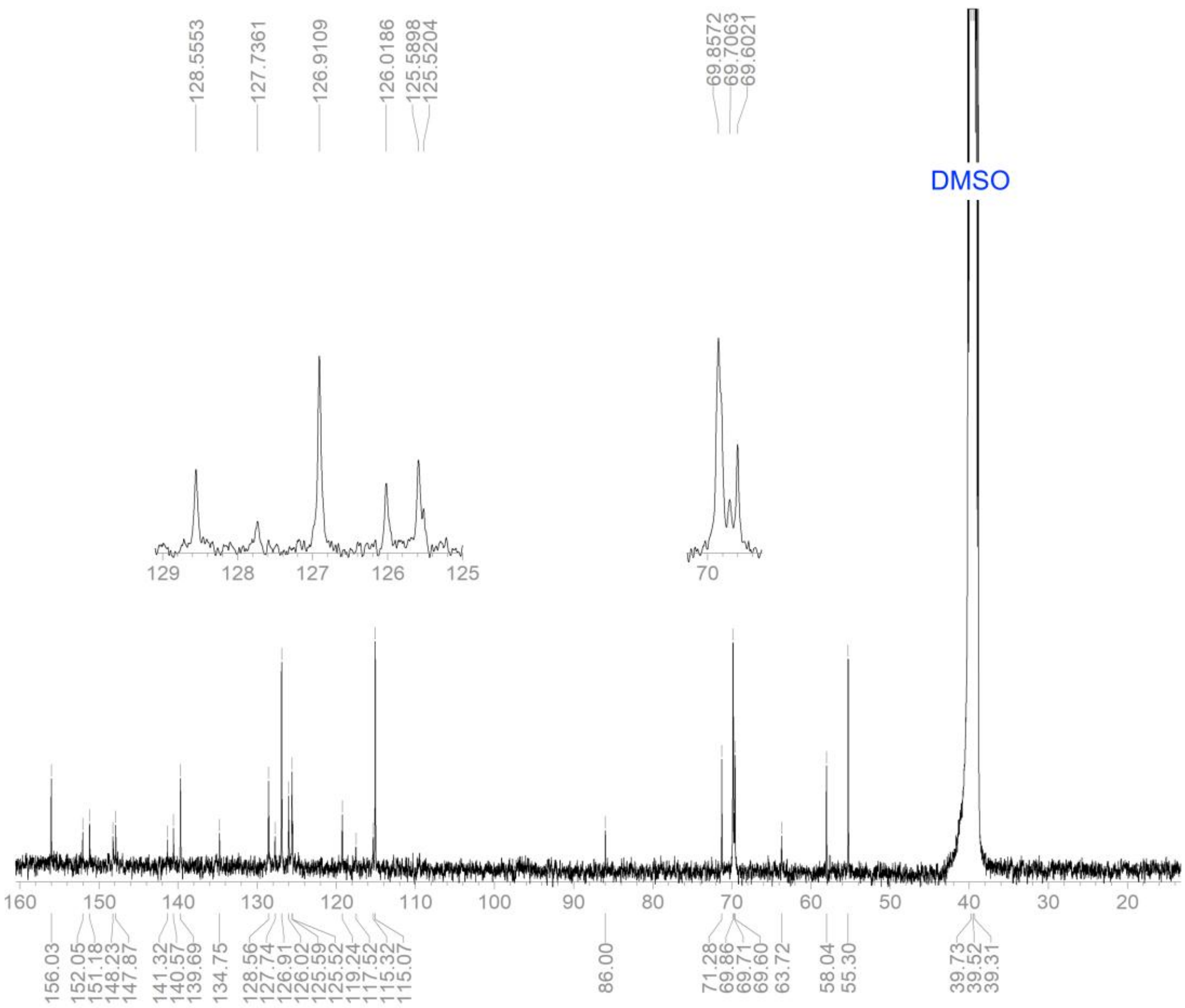

Figure S29. ^{13}C NMR spectrum (100 MHz) of (*S*,*S*)-**1** in DMSO-d$_6$ at 25 °C.

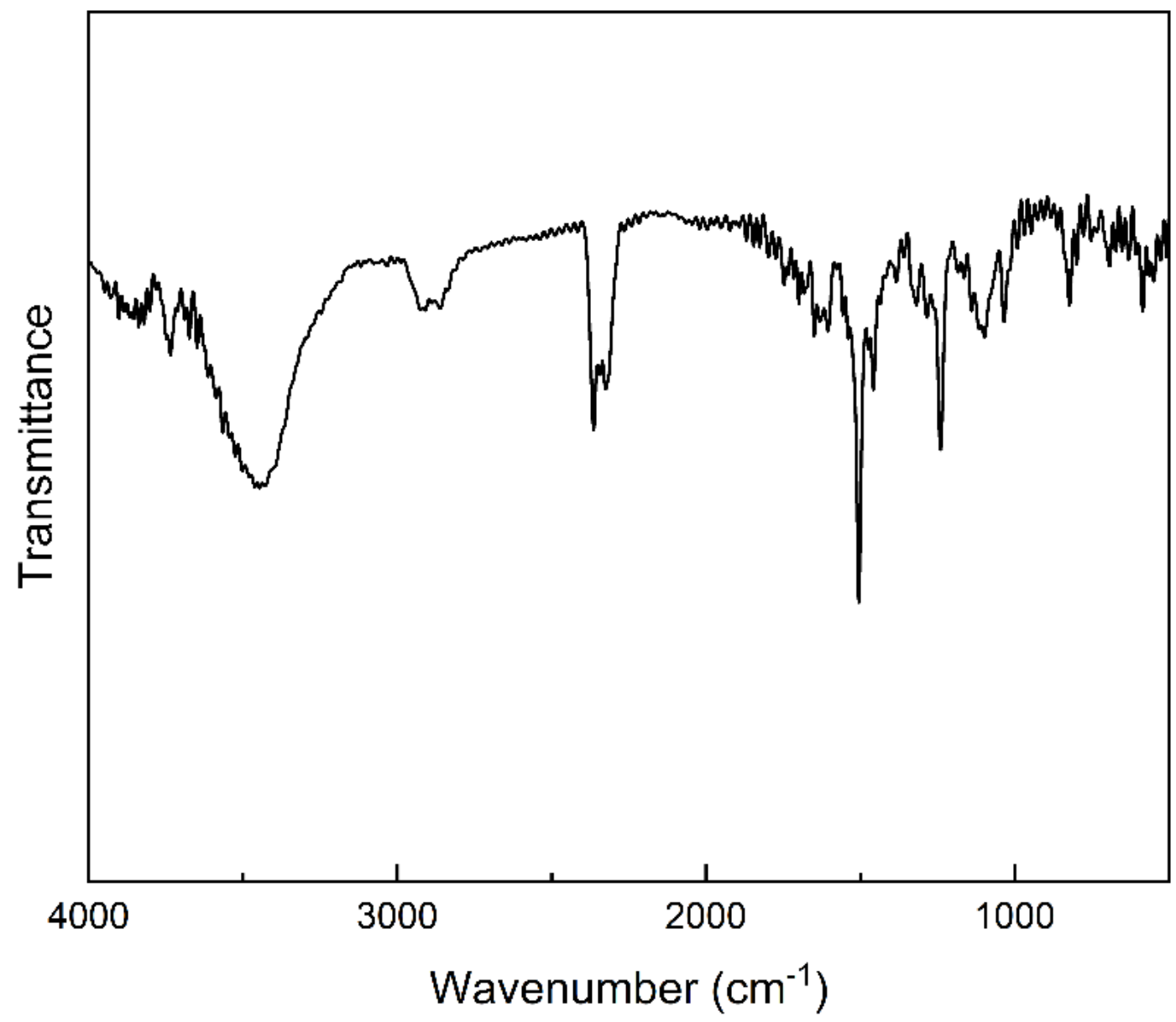

Figure S30. FT-IR spectrum of (*S*,*S*)-**1** at 25 °C (KBr).

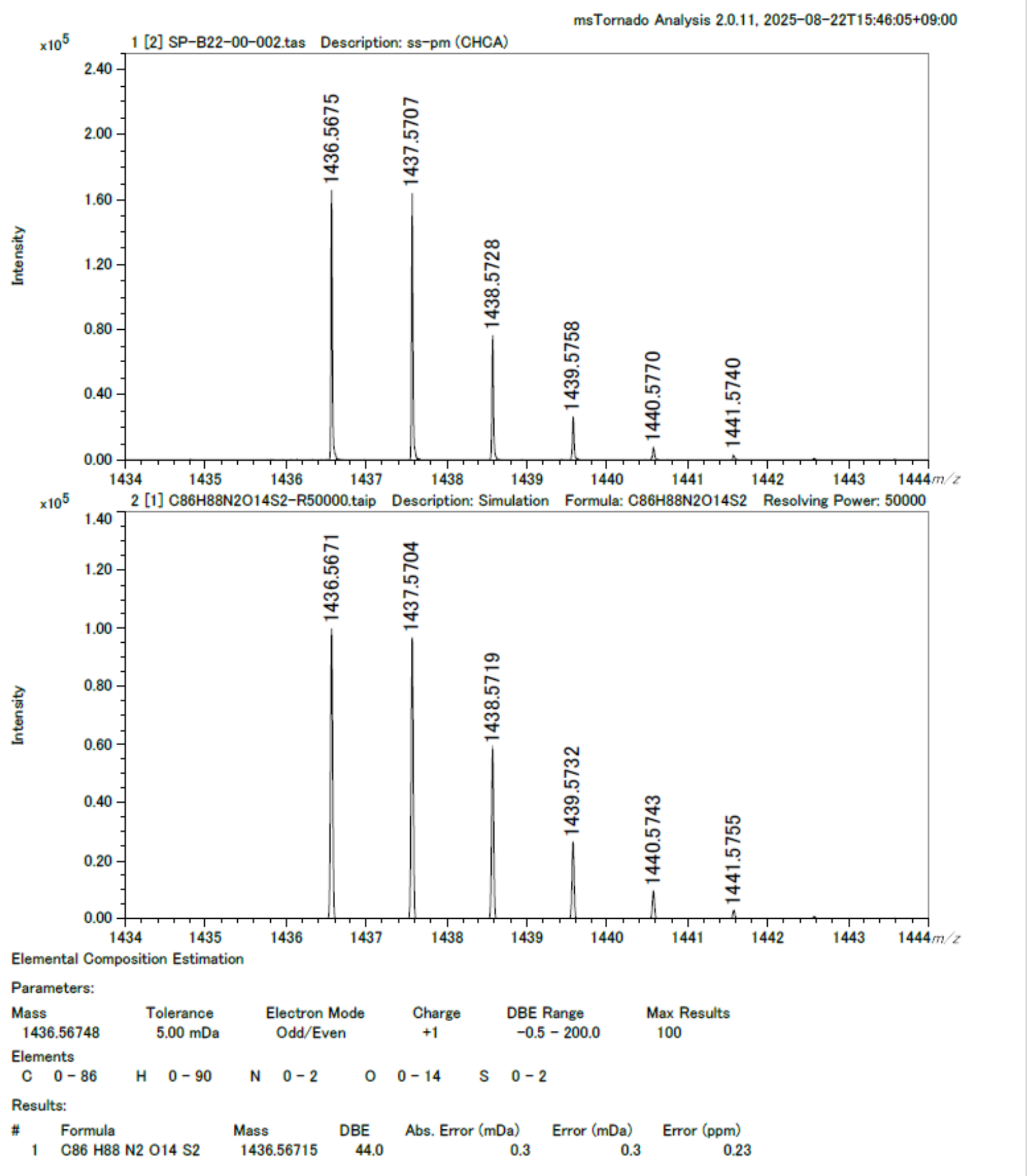

Figure S31. Observed (upper) and simulated (lower) high-resolution MALDI-TOF mass spectra of (*S*,*S*)-**1**.

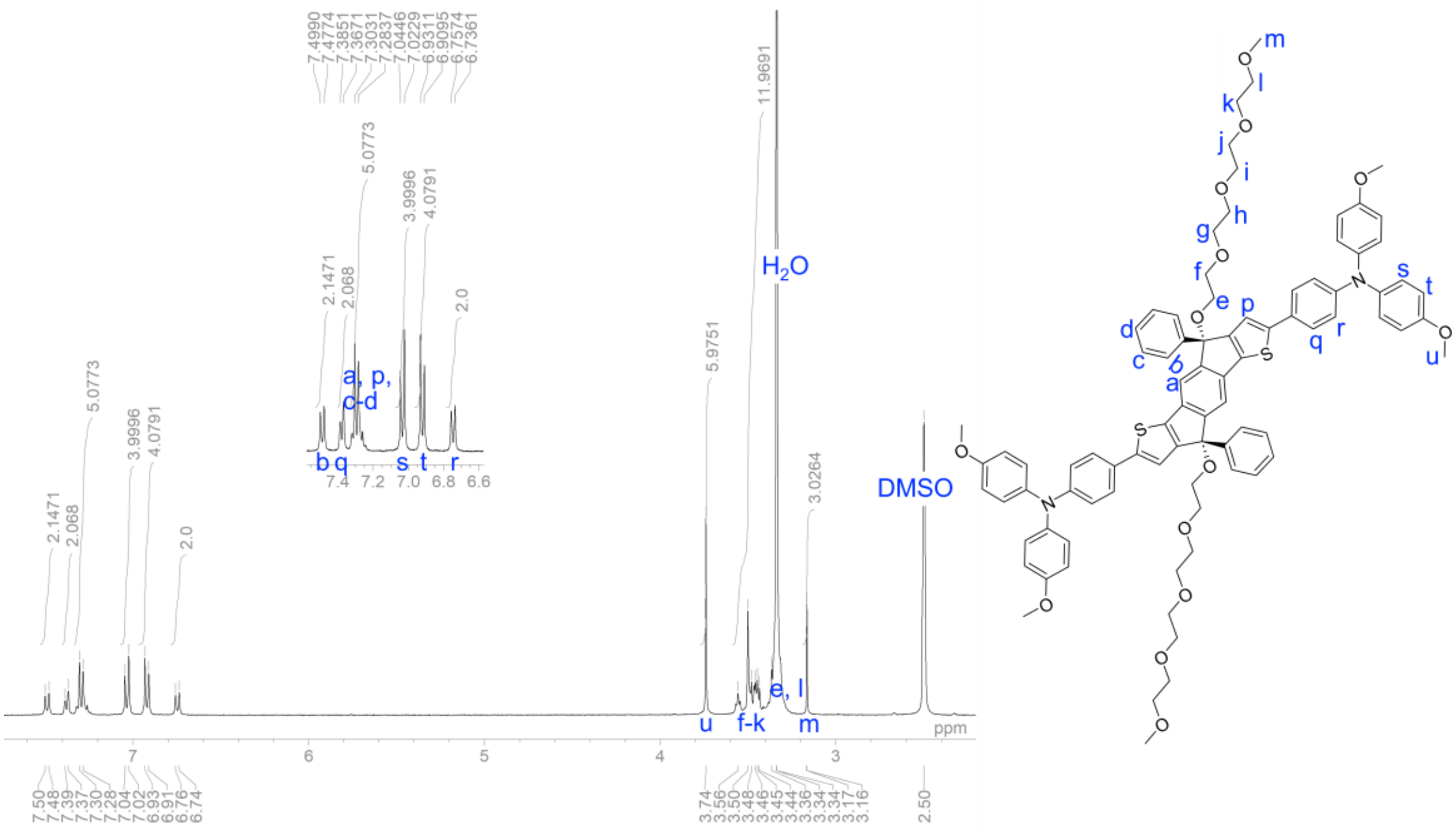

Figure S32. ^{1}H NMR spectrum (400 MHz) of (*R*,*R*)-**1** in DMSO-d$_6$ at 25 °C.

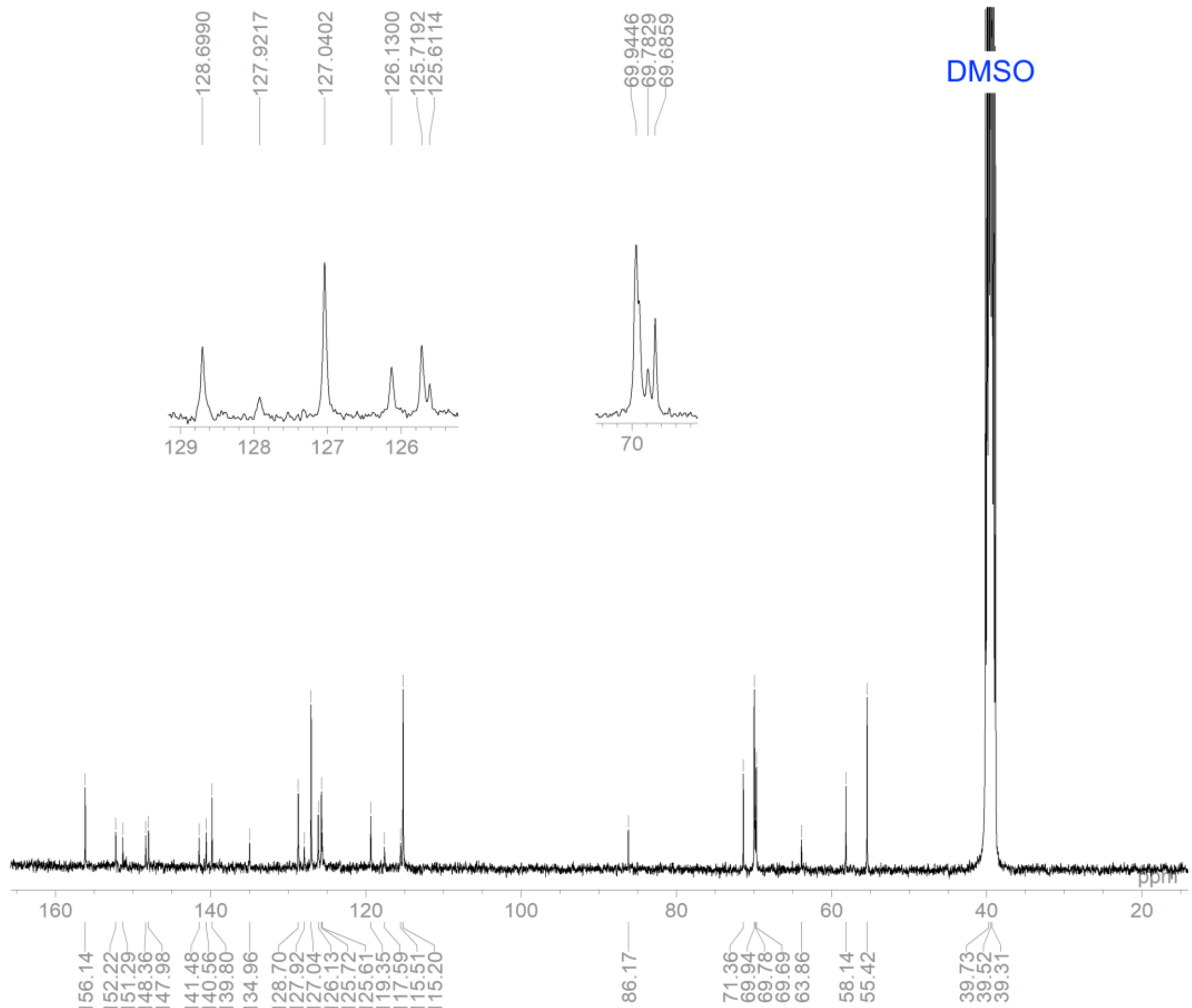

Figure S33. ^{13}C NMR spectrum (100 MHz) of (*R*,*R*)-**1** in DMSO-d$_6$ at 25 °C.

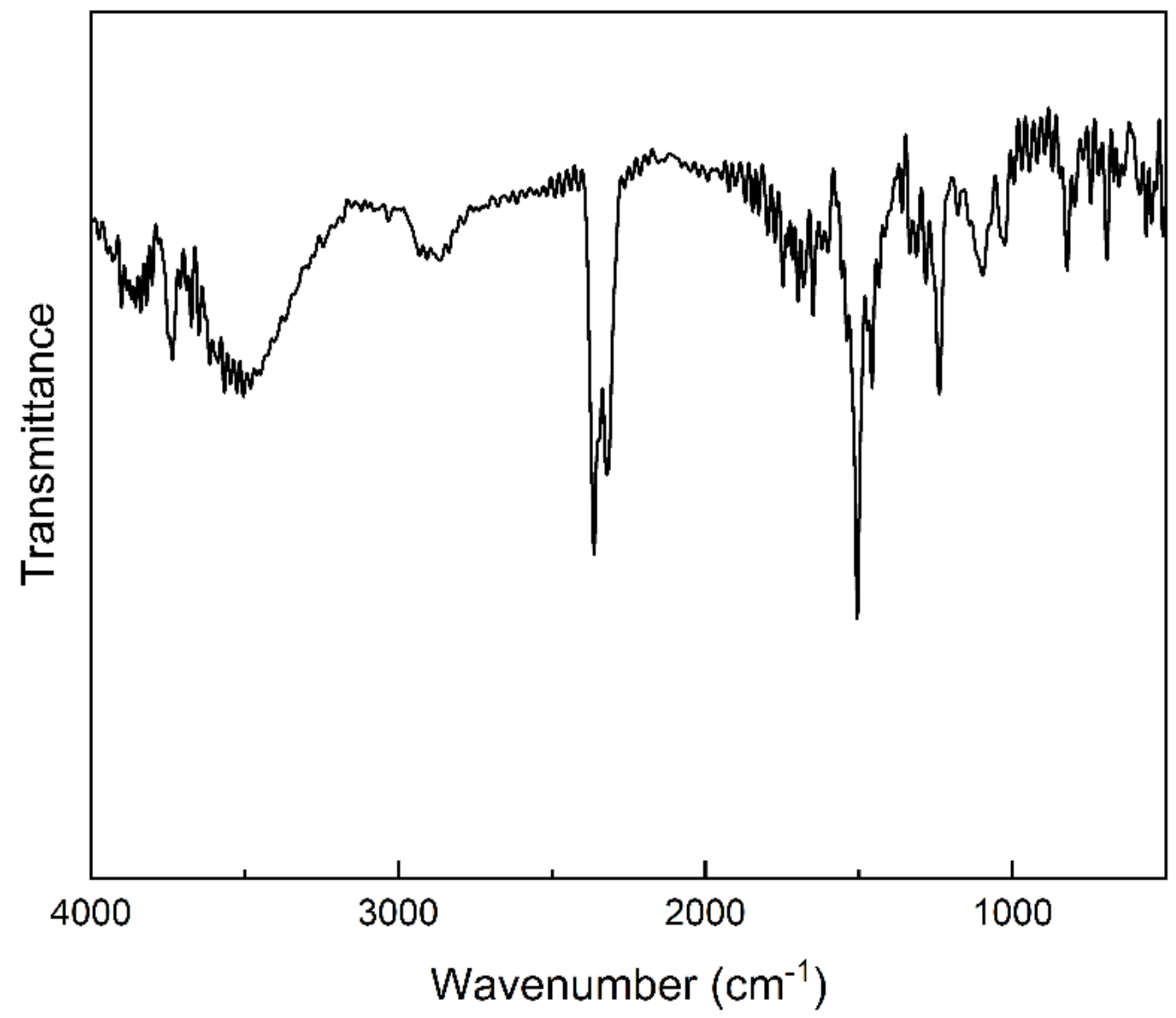

Figure S34. FT-IR spectrum of (*R*,*R*)-**1** at 25 °C (KBr).

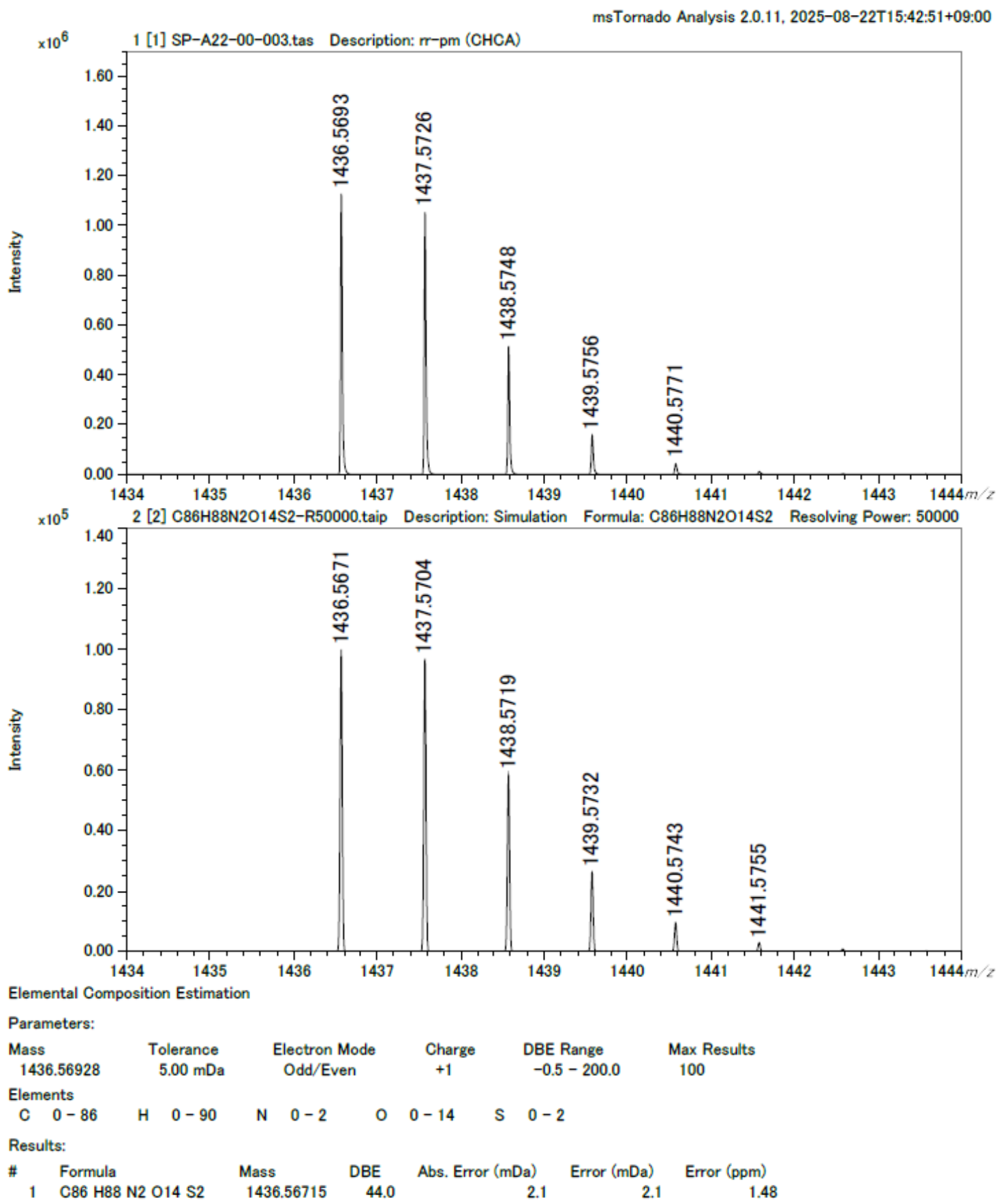

Figure S35. Observed (upper) and simulated (lower) high-resolution MALDI-TOF mass spectra of (*R*,*R*)-**1**.

9. Supporting Tables

Table S1. Summary of PSCs resistance [a]

Active layers	R_s [Ω·cm^2]	R_{sh} [Ω·cm^2]	*n* of devices
Control[b]	2.38 ± 0.38	2514 ± 1208	8
meso-**1**	2.26 ± 0.43	2622 ± 1861	7
rac-**1**	1.95 ± 0.60	3309 ± 1072	9
(*R*,*R*)-**1**	1.86 ± 0.30	3449 ± 1087	8

[a] FTO/cTiO_2/mpTiO_2/PVK/(passivation)/doped Spiro-OMeTAD/Au. The average R_s and R_{sh} values were calculated over the multiple devices. [b] A control sample. Note that neat CB treatment was applied.

Table S2. Summary of PSC performances[a]

Active layers	J_{SC} [mA cm^{-2}]	V_{OC} [V]	FF [%]	PCE [%]	*n* of devices
Control[b]	23.23 ± 0.76 (23.62)	1.060 ± 0.01 (1.061)	74.7 ± 3 (78.3)	18.41 ± 1.02 (19.63)	5
(*R*,*R*)-**1**	23.46 ± 0.98 (24.27)	1.068 ± 0.01 (1.085)	76.2 ± 3 (79.6)	19.10 ± 1.09 (20.96)	5
(*S*,*S*)-**1**	23.95 ± 0.47 (24.28)	1.068 ± 0.01 (1.090)	74.7 ± 2 (78.7)	19.11 ± 0.82 (20.83)	8

[a] FTO/cTiO_2/mpTiO_2/PVK/(passivation)/doped Spiro-OMeTAD/Au. The values in parentheses are values of best-performing cell in the reverse scan. The average J_{SC}, V_{OC}, FF and PCE values were calculated over the reverse scans of the multiple devices. [b] A control sample. Note that neat CB treatment was applied.

Table S3. Initial performance parameters of the stability-tracked devices.[a]

Active layers	J_{SC} [mA cm^{-2}]	V_{OC} [V]	FF [%]	PCE [%]
Control[b]	23.45	1.048	75.0	18.42
meso-**1**	23.30	1.058	75.3	18.57
rac-**1**	23.80	1.036	76.2	18.80
(*R*,*R*)-**1**	23.20	1.055	75.3	18.40

[a] Stability tracking was conducted on devices that showed longer operational lifetimes. The values were collected from the reverse scans of the devices. [b] A control sample. Note that neat CB treatment was applied.

Table S4. Summary of TRPL analyses.[a]

Samples	τ_1 / ns (intensity / %)	τ_2 / ns (intensity / %)	T_{ave} / ns
PVK/CB	11.9 (1.7)	1044.6 (98.3)	1027.2
PVK/*meso*-**1**	10.6 (4.6)	729.3 (95.4)	695.9
PVK/*rac*-**1**	19.3 (6.6)	531.5 (93.4)	497.9
PVK/(*R*,*R*)-**1**	23.2 (15.4)	411.9 (84.6)	351.9

[a]Fitted by double exponential functions: $f\ (t) = A_1\, exp(-\frac{t}{\tau_1}) + A_2\, exp(-\frac{t}{\tau_2})$. Average lifetime = $(A_1\tau_1 + A_2\tau_2)\ (A_1 + A_2)^{-1}$.

Table S5. Cartesian coordinates of the optimized geometry of *meso*-**1** (a singlet state in vacuum) at the B3LYP/6-31G(d,p) level. Total energy is -4058.39446118 hartree.

C	-5.27707	-0.38595	-0.18493
C	-4.54689	-1.55294	-0.32714
C	-3.14703	-1.33597	-0.31012
C	-2.80370	-0.01380	-0.13223
S	-4.19564	1.00751	-0.01812
C	5.29937	0.38540	0.03609
C	4.56954	1.54885	0.20210
C	3.17585	1.32519	0.16302
C	2.82844	0.00932	-0.02583
S	4.22169	-1.00555	-0.18138
C	-1.36412	0.16269	-0.05344
C	-0.56049	1.30000	0.11116
C	0.81761	1.12627	0.13864
C	1.38673	-0.16204	-0.03657
C	0.58347	-1.29741	-0.20287
C	-0.79091	-1.12044	-0.19777
C	1.92731	2.18720	0.32989
C	-1.88318	-2.19562	-0.38784
C	-1.79594	-3.28140	0.69540
C	1.89333	3.33726	-0.68681
C	-1.01731	-4.42445	0.46946
C	-0.87757	-5.39714	1.45982
C	-1.50848	-5.23914	2.69427
C	-2.28150	-4.10132	2.92923
C	-2.42619	-3.13114	1.93719
C	1.27668	4.54567	-0.33392
C	1.28528	5.63165	-1.20914
C	1.91835	5.53021	-2.44873
C	2.54228	4.33397	-2.80442
C	2.52863	3.24523	-1.93166

C	3.15525	3.43003	2.03325
O	1.99790	2.68822	1.67629
C	-2.68389	-3.54659	-2.23386
O	-1.64236	-2.74795	-1.69063
C	12.62854	-1.97942	0.36024
C	11.58804	-1.44837	-0.40866
C	11.16046	-2.16263	-1.54215
C	11.74802	-3.37269	-1.88060
C	12.79742	-3.89412	-1.10913
C	13.24061	-3.18569	0.01274
C	7.37356	-0.99981	0.33771
C	6.75130	0.21839	0.00720
C	7.59098	1.29090	-0.35325
C	8.97167	1.15788	-0.37659
C	9.58474	-0.06374	-0.03840
C	8.75474	-1.14181	0.31922
C	11.83138	0.89280	0.28707
C	12.94388	1.21938	-0.50787
C	13.77809	2.27250	-0.16219
C	13.51288	3.04440	0.97865
C	12.40346	2.73463	1.77286
C	11.58173	1.65845	1.43102
N	10.98885	-0.20205	-0.06360
O	13.31443	-5.08547	-1.53569
C	14.37506	-5.65849	-0.78919
O	14.38863	4.06518	1.22347
C	14.16611	4.87675	2.36476
C	-12.60317	1.79845	0.76185
C	-11.56776	1.49695	-0.12832
C	-11.14858	2.49038	-1.03094
C	-11.73940	3.74535	-1.02771
C	-12.78375	4.03727	-0.13734
C	-13.21854	3.05210	0.75573
C	-7.35272	0.87365	0.46100
C	-6.72869	-0.21680	-0.17337
C	-7.56761	-1.15899	-0.80094
C	-8.94833	-1.02632	-0.78955
C	-9.56339	0.06529	-0.14641
C	-8.73374	1.01474	0.47864
C	-11.80952	-0.94511	-0.10098
C	-12.91343	-1.04484	-0.96542
C	-13.74896	-2.15144	-0.92369
C	-13.49357	-3.20156	-0.02926
C	-12.39268	-3.11739	0.83028

C	-11.56967	-1.98964	0.79803
N	-10.96623	0.20413	-0.13550
O	-13.30443	5.29762	-0.22450
C	-14.36159	5.64631	0.65423
O	-14.36956	-4.24940	-0.07623
C	-14.15855	-5.33732	0.80880
H	-5.01925	-2.52527	-0.39706
H	5.03275	2.51294	0.37204
H	-1.00687	2.28610	0.19150
H	1.02110	-2.28194	-0.33345
H	-0.52878	-4.54656	-0.49132
H	-0.27391	-6.27921	1.26516
H	-1.39982	-5.99597	3.46569
H	-2.77768	-3.96779	3.88637
H	-3.03272	-2.25197	2.12919
H	0.78674	4.62267	0.63096
H	0.79664	6.55831	-0.92095
H	1.92510	6.37515	-3.13119
H	3.03930	4.24342	-3.76609
H	3.01566	2.31989	-2.22082
H	3.71429	2.91585	2.82284
H	3.81430	3.67792	1.20747
H	2.81597	4.38631	2.45630
H	-2.28360	-3.98267	-3.15220
H	-2.98201	-4.35819	-1.55666
H	-3.56686	-2.94548	-2.48352
H	12.96993	-1.44018	1.23772
H	10.35955	-1.76121	-2.15421
H	11.42062	-3.92797	-2.75335
H	14.04709	-3.56328	0.62987
H	6.76630	-1.84487	0.64966
H	7.14969	2.23615	-0.65373
H	9.58755	1.99897	-0.67510
H	9.20079	-2.09014	0.59730
H	13.15049	0.63556	-1.39876
H	14.63831	2.52772	-0.77221
H	12.17567	3.30782	2.66359
H	10.73105	1.41559	2.05929
H	14.63275	-6.58950	-1.29621
H	15.25645	-5.00432	-0.76767
H	14.07165	-5.88214	0.24186
H	14.96501	5.61972	2.36745
H	13.19705	5.39018	2.31541
H	14.21383	4.29366	3.29368

H	-12.93807	1.04181	1.46374
H	-10.35192	2.27018	-1.73388
H	-11.41893	4.51569	-1.72128
H	-14.02113	3.24776	1.45669
H	-6.74690	1.61041	0.98109
H	-7.12554	-1.99235	-1.33828
H	-9.56279	-1.76135	-1.29736
H	-9.18040	1.85865	0.99223
H	-13.11246	-0.24268	-1.66838
H	-14.60282	-2.23273	-1.58815
H	-12.17315	-3.90877	1.53693
H	-10.72588	-1.92463	1.47717
H	-14.62325	6.67850	0.41704
H	-15.24188	5.00789	0.50379
H	-14.05226	5.58581	1.70575
H	-14.95658	-6.05237	0.60385
H	-13.18844	-5.82053	0.63424
H	-14.21719	-5.02374	1.85918

Table S6. Cartesian coordinates of the optimized geometry of (*S*,*S*)-**1** (a singlet state in vacuum) at the B3LYP/6-31G(d,p) level. Total energy is -4058.39293193 hartree.

C	-4.75859	-1.39747	1.00870
C	-3.88318	-2.46164	0.87989
C	-2.56295	-2.05754	0.56257
C	-2.41975	-0.69049	0.47150
S	-3.91574	0.13707	0.73681
C	5.43028	0.84025	-0.90016
C	4.54514	1.90117	-0.82919
C	3.21373	1.48338	-0.61335
C	3.06650	0.12065	-0.51820
S	4.57813	-0.70185	-0.70150
C	-1.04339	-0.30976	0.20675
C	-0.42458	0.93783	0.04236
C	0.93813	0.95944	-0.22718
C	1.68086	-0.24829	-0.29048
C	1.06461	-1.49354	-0.11235
C	-0.29986	-1.50852	0.12896
C	1.86103	2.17663	-0.47351
C	-1.21139	-2.73849	0.33430
C	-0.73578	-3.59985	1.51387
C	1.86869	3.21162	0.66057
C	0.16523	-4.64884	1.28623
C	0.65466	-5.40945	2.34854

C	0.25622	-5.12911	3.65604
C	-0.63696	-4.08309	3.89190
C	-1.13093	-3.32578	2.82943
C	1.00446	4.31311	0.59175
C	0.98386	5.26677	1.60941
C	1.83349	5.13946	2.70916
C	2.70329	4.05084	2.78149
C	2.71984	3.09333	1.76659
C	2.61172	3.73024	-2.19922
O	1.63299	2.80566	-1.74640
C	-2.11547	-4.48138	-1.08670
O	-1.13684	-3.46915	-0.89884
C	13.03244	-0.38420	-1.29367
C	11.83056	-0.15308	-1.97043
C	11.38537	-1.11621	-2.89348
C	12.11492	-2.27468	-3.11634
C	13.32443	-2.49457	-2.44030
C	13.78427	-1.53724	-1.52987
C	7.72440	-0.16132	-0.68124
C	6.87581	0.87440	-1.11454
C	7.47886	1.96610	-1.77020
C	8.84951	2.02425	-1.97631
C	9.69039	0.98518	-1.53399
C	9.09755	-0.11089	-0.88127
C	11.77413	2.28712	-1.70956
C	12.71320	2.61189	-2.70406
C	13.40043	3.81631	-2.66449
C	13.15405	4.74198	-1.63974
C	12.21514	4.43389	-0.64925
C	11.54439	3.20949	-0.68309
N	11.08460	1.04031	-1.74547
O	13.97083	-3.66074	-2.74174
C	15.19747	-3.93333	-2.08486
O	13.87497	5.90198	-1.69932
C	13.66374	6.87213	-0.68697
C	-12.04004	-0.07766	3.15719
C	-11.14970	-0.39572	2.12686
C	-11.07514	0.46304	1.01583
C	-11.85674	1.60709	0.95105
C	-12.75422	1.91456	1.98477
C	-12.84702	1.06009	3.08860
C	-6.85494	-0.33382	1.89506
C	-6.18856	-1.42635	1.30910
C	-6.96084	-2.56968	1.02310

C	-8.31672	-2.62335	1.31099
C	-8.97318	-1.52636	1.90186
C	-8.21133	-0.37819	2.18759
C	-10.96981	-2.80196	2.57597
C	-12.18335	-3.20305	1.99086
C	-12.79893	-4.38542	2.37482
C	-12.20853	-5.21277	3.34172
C	-10.99621	-4.82821	3.92502
C	-10.39452	-3.62515	3.54974
N	-10.35210	-1.57589	2.19123
O	-13.48551	3.05661	1.81689
C	-14.40779	3.41519	2.83279
O	-12.88977	-6.35935	3.63982
C	-12.33739	-7.23140	4.61231
H	-4.18944	-3.48620	1.05290
H	4.86138	2.93480	-0.89668
H	-0.99480	1.85581	0.14343
H	1.63788	-2.41446	-0.14593
H	0.47222	-4.86656	0.26886
H	1.34863	-6.22207	2.15259
H	0.63680	-5.72100	4.48339
H	-0.95502	-3.85639	4.90562
H	-1.82858	-2.51752	3.02372
H	0.34626	4.41072	-0.26506
H	0.30274	6.11057	1.54170
H	1.81773	5.88125	3.50240
H	3.37028	3.94182	3.63200
H	3.39936	2.25034	1.83520
H	2.35451	3.95529	-3.23723
H	2.59084	4.66164	-1.62121
H	3.62654	3.31147	-2.17032
H	-1.83213	-5.01067	-1.99953
H	-2.13823	-5.19742	-0.25425
H	-3.11762	-4.05602	-1.22104
H	13.38934	0.34929	-0.57821
H	10.45885	-0.94922	-3.43279
H	11.77483	-3.02107	-3.82660
H	14.71302	-1.67933	-0.99037
H	7.30600	-1.00920	-0.14620
H	6.85545	2.76629	-2.15685
H	9.27880	2.87111	-2.50020
H	9.72313	-0.91889	-0.51868
H	12.90209	1.90746	-3.50736
H	14.12664	4.07089	-3.42939

H	12.00736	5.12507	0.15881
H	10.82773	2.97150	0.09608
H	15.53759	-4.89667	-2.46769
H	15.95508	-3.17019	-2.30598
H	15.06897	-4.00348	-0.99686
H	14.32522	7.70635	-0.92528
H	12.62525	7.22791	-0.67561
H	13.91844	6.48371	0.30775
H	-12.10922	-0.73098	4.02074
H	-10.39541	0.22650	0.20388
H	-11.80220	2.27351	0.09660
H	-13.52883	1.27156	3.90358
H	-6.29398	0.55854	2.15839
H	-6.49522	-3.41900	0.53267
H	-8.88411	-3.51303	1.06144
H	-8.68789	0.47645	2.65445
H	-12.64192	-2.57519	1.23407
H	-13.73612	-4.69891	1.92693
H	-10.51900	-5.44222	4.67940
H	-9.46172	-3.32606	4.01653
H	-14.87754	4.34208	2.50058
H	-15.18110	2.64789	2.96832
H	-13.90615	3.59000	3.79344
H	-13.02840	-8.07188	4.69140
H	-11.35019	-7.60406	4.30956
H	-12.24978	-6.74400	5.59203

Table S7. Cartesian coordinates of the optimized geometry of (*R*,*R*)-**1** (a singlet state in vacuum) at the B3LYP/6-31G(d,p) level. Total energy is -4058.38540971 hartree.

C	-4.75859	-1.39747	-1.00870
C	-3.88318	-2.46164	-0.87989
C	-2.56295	-2.05754	-0.56257
C	-2.41975	-0.69049	-0.47150
S	-3.91574	0.13707	-0.73681
C	5.43028	0.84025	0.90016
C	4.54514	1.90117	0.82919
C	3.21373	1.48338	0.61335
C	3.06650	0.12065	0.51820
S	4.57813	-0.70185	0.70150
C	-1.04339	-0.30976	-0.20675
C	-0.42458	0.93783	-0.04236
C	0.93813	0.95944	0.22718
C	1.68086	-0.24829	0.29048

C	1.06461	-1.49354	0.11235
C	-0.29986	-1.50852	-0.12896
C	1.86103	2.17663	0.47351
C	-1.21139	-2.73849	-0.33430
C	-0.73578	-3.59985	-1.51387
C	1.86869	3.21162	-0.66057
C	0.16523	-4.64884	-1.28623
C	0.65466	-5.40945	-2.34854
C	0.25622	-5.12911	-3.65604
C	-0.63696	-4.08309	-3.89190
C	-1.13093	-3.32578	-2.82943
C	1.00446	4.31311	-0.59175
C	0.98386	5.26677	-1.60941
C	1.83349	5.13946	-2.70916
C	2.70329	4.05084	-2.78149
C	2.71984	3.09333	-1.76659
C	2.61172	3.73024	2.19922
O	1.63299	2.80566	1.74640
C	-2.11547	-4.48138	1.08670
O	-1.13684	-3.46915	0.89884
C	13.03244	-0.38420	1.29367
C	11.83056	-0.15308	1.97043
C	11.38537	-1.11621	2.89348
C	12.11492	-2.27468	3.11634
C	13.32443	-2.49457	2.44030
C	13.78427	-1.53724	1.52987
C	7.72440	-0.16132	0.68124
C	6.87581	0.87440	1.11454
C	7.47886	1.96610	1.77020
C	8.84951	2.02425	1.97631
C	9.69039	0.98518	1.53399
C	9.09755	-0.11089	0.88127
C	11.77413	2.28712	1.70956
C	12.71320	2.61189	2.70406
C	13.40043	3.81631	2.66449
C	13.15405	4.74198	1.63974
C	12.21514	4.43389	0.64925
C	11.54439	3.20949	0.68309
N	11.08460	1.04031	1.74547
O	13.97083	-3.66074	2.74174
C	15.19747	-3.93333	2.08486
O	13.87497	5.90198	1.69932
C	13.66374	6.87213	0.68697
C	-12.04004	-0.07766	-3.15719

C	-11.14970	-0.39572	-2.12686
C	-11.07514	0.46304	-1.01583
C	-11.85674	1.60709	-0.95105
C	-12.75422	1.91456	-1.98477
C	-12.84702	1.06009	-3.08860
C	-6.85494	-0.33382	-1.89506
C	-6.18856	-1.42635	-1.30910
C	-6.96084	-2.56968	-1.02310
C	-8.31672	-2.62335	-1.31099
C	-8.97318	-1.52636	-1.90186
C	-8.21133	-0.37819	-2.18759
C	-10.96981	-2.80196	-2.57597
C	-12.18335	-3.20305	-1.99086
C	-12.79893	-4.38542	-2.37482
C	-12.20853	-5.21277	-3.34172
C	-10.99621	-4.82821	-3.92502
C	-10.39452	-3.62515	-3.54974
N	-10.35210	-1.57589	-2.19123
O	-13.48551	3.05661	-1.81689
C	-14.40779	3.41519	-2.83279
O	-12.88977	-6.35935	-3.63982
C	-12.33739	-7.23140	-4.61231
H	-4.18944	-3.48620	-1.05290
H	4.86138	2.93480	0.89668
H	-0.99480	1.85581	-0.14343
H	1.63788	-2.41446	0.14593
H	0.47222	-4.86656	-0.26886
H	1.34863	-6.22207	-2.15259
H	0.63680	-5.72100	-4.48339
H	-0.95502	-3.85639	-4.90562
H	-1.82858	-2.51752	-3.02372
H	0.34626	4.41072	0.26506
H	0.30274	6.11057	-1.54170
H	1.81773	5.88125	-3.50240
H	3.37028	3.94182	-3.63200
H	3.39936	2.25034	-1.83520
H	2.35451	3.95529	3.23723
H	2.59084	4.66164	1.62121
H	3.62654	3.31147	2.17032
H	-1.83213	-5.01067	1.99953
H	-2.13823	-5.19742	0.25425
H	-3.11762	-4.05602	1.22104
H	13.38934	0.34929	0.57821
H	10.45885	-0.94922	3.43279

H	11.77483	-3.02107	3.82660
H	14.71302	-1.67933	0.99037
H	7.30600	-1.00920	0.14620
H	6.85545	2.76629	2.15685
H	9.27880	2.87111	2.50020
H	9.72313	-0.91889	0.51868
H	12.90209	1.90746	3.50736
H	14.12664	4.07089	3.42939
H	12.00736	5.12507	-0.15881
H	10.82773	2.97150	-0.09608
H	15.53759	-4.89667	2.46769
H	15.95508	-3.17019	2.30598
H	15.06897	-4.00348	0.99686
H	14.32522	7.70635	0.92528
H	12.62525	7.22791	0.67561
H	13.91844	6.48371	-0.30775
H	-12.10922	-0.73098	-4.02074
H	-10.39541	0.22650	-0.20388
H	-11.80220	2.27351	-0.09660
H	-13.52883	1.27156	-3.90358
H	-6.29398	0.55854	-2.15839
H	-6.49522	-3.41900	-0.53267
H	-8.88411	-3.51303	-1.06144
H	-8.68789	0.47645	-2.65445
H	-12.64192	-2.57519	-1.23407
H	-13.73612	-4.69891	-1.92693
H	-10.51900	-5.44222	-4.67940
H	-9.46172	-3.32606	-4.01653
H	-14.87754	4.34208	-2.50058
H	-15.18110	2.64789	-2.96832
H	-13.90615	3.59000	-3.79344
H	-13.02840	-8.07188	-4.69140
H	-11.35019	-7.60406	-4.30956
H	-12.24978	-6.74400	-5.59203

10. Supporting References

S1. S. Li, F. Ishiwari, S. Zorn, K. Murotani, M. Pylnev, K. Taniguchi and A. Saeki, Chiral bifacial indacenodithiophene-based π-conjugated polymers with chirality-induced spin selectivity *Chem. Commun.*, **2024**, *60*, 10870-10873.

S2. S. Li, F. Ishiwari, S. X. Li, Y. Yakiyama, A. Saeki, Chiral Bifacial Non-Fullerene Acceptors with Chirality-Induced Spin Selectivity: A Homochiral Strategy to Improve Organic Solar Cell Performance *Angew. Chem. Int. Ed.* **2025**, e202518505.

S3. M. J. Frisch, G. W. Trucks, H. B. Schlegel, G. E. Scuseria, M. A. Robb, J. R. Cheeseman, G. Scalmani, V. Barone, G. A. Petersson, H. Nakatsuji, X. Li, M. Caricato, A. V. Marenich, J. Bloino, B. G. Janesko, R. Gomperts, B. Mennucci, H. P. Hratchian, J. V. Ortiz, A. F. Izmaylov, J. L. Sonnenberg, D. Williams-Young, F. Ding, F. Lipparini, F. Egidi, J. Goings, B. Peng, A. Petrone, T. Henderson, D. Ranasinghe, V. G. Zakrzewski, J. Gao, N. Rega, G. Zheng, W. Liang, M. Hada, M. Ehara, K. Toyota, R. Fukuda, J. Hasegawa, M. Ishida, T. Nakajima, Y. Honda, O. Kitao, H. Nakai, T. Vreven, K. Throssell, J. A. Montgomery, Jr., J. E. Peralta, F. Ogliaro, M. J. Bearpark, J. J. Heyd, E. N. Brothers, K. N. Kudin, V. N. Staroverov, T. A. Keith, R. Kobayashi, J. Normand, K. Raghavachari, A. P. Rendell, J. C. Burant, S. S. Iyengar, J. Tomasi, M. Cossi, J. M. Millam, M. Klene, C. Adamo, R. Cammi, J. W. Ochterski, R. L. Martin, K. Morokuma, O. Farkas, J. B. Foresman and D. J. Fox, Gaussian, Inc., Gaussian 16, Revision B.01, Wallingford CT, **2016**.

S4. T. Lu, F. Chen, Multiwfn: A Multifunctional Wavefunction Analyzer, *J. Comput. Chem.* **2012**, *33*, 580–592.
